# Variation in Health Care Access and Quality Among US States and High-Income Countries With Universal Health Insurance Coverage

**DOI:** 10.1001/jamanetworkopen.2021.14730

**Published:** 2021-06-28

**Authors:** Marcia R. Weaver, Vishnu Nandakumar, Jonah Joffe, Ryan M. Barber, Nancy Fullman, Arjun Singh, Gianna W. Sparks, Jamal Yearwood, Rafael Lozano, Christopher J. L. Murray, Diana Ngo

**Affiliations:** 1 Departments of Health Metrics Sciences and Global Health, Institute for Health Metrics and Evaluation, University of Washington, Seattle; 2 Institute for Health Metrics and Evaluation, University of Washington, Seattle; 3 Department of Economics, Occidental College, Los Angeles, California

## Abstract

**Importance:**

Based on mortality estimates for 32 causes of death that are amenable to health care, the US health care system did not perform as well as other high-income countries, scoring 88.7 out of 100 on the 2016 age-standardized Healthcare Access and Quality (HAQ) index.

**Objective:**

To compare US age-specific HAQ scores with those of high-income countries with universal health insurance coverage and compare scores among US states with varying insurance coverage.

**Design, Setting, and Participants:**

This cross-sectional study used 2016 Global Burden of Diseases, Injuries, and Risk Factor study results for cause-specific mortality with adjustments for behavioral and environmental risks to estimate the age-specific HAQ indices. The US national age-specific HAQ scores were compared with high-income peers (Canada, western Europe, high-income Asia Pacific countries, and Australasia) in 1990, 2000, 2010, and 2016, and the 2016 scores among US states were also analyzed. The Public Use Microdata Sample of the American Community Survey was used to estimate insurance coverage and the median income per person by age and state. Age-specific HAQ scores for each state in 2010 and 2016 were regressed based on models with age fixed effects and age interaction with insurance coverage, median income, and year. Data were analyzed from April to July 2018 and July to September 2020.

**Main Outcomes and Measures:**

The age-specific HAQ indices were the outcome measures.

**Results:**

In 1990, US age-specific HAQ scores were similar to peers but increased less from 1990 to 2016 than peer locations for ages 15 years or older. For example, for ages 50 to 54 years, US scores increased from 77.1 to 82.1 while high-income Asia Pacific scores increased from 71.6 to 88.2. In 2016, several states had scores comparable with peers, with large differences in performance across states. For ages 15 years or older, the age-specific HAQ scores were 85 or greater for all ages in 3 states (Connecticut, Massachusetts, and Minnesota) and 75 or less for at least 1 age category in 6 states. In regression analysis estimates with state-fixed effects, insurance coverage coefficients for ages 20 to 24 years were 0.059 (99% CI, 0.006-0.111); 45 to 49 years, 0.088 (99% CI, 0.009-0.167); and 50 to 54 years, 0.101 (99% CI, 0.013-0.189). A 10% increase in insurance coverage was associated with point increases in HAQ scores among the ages of 20 to 24 years (0.59 [99% CI, 0.06-1.11]), 45 to 49 years (0.88 [99% CI, 0.09-1.67]), and 50 to 54 years (1.01 [99% CI, 0.13-1.89]).

**Conclusions and Relevance:**

In this cross-sectional study, the US age-specific HAQ scores for ages 15 to 64 years were low relative to high-income peer locations with universal health insurance coverage. Among US states, insurance coverage was associated with higher HAQ scores for some ages. Further research with causal models and additional explanations is warranted.

## Key Points

**Question:**

Does personal health care access and quality vary across ages among high-income countries and US states, and does any observed variation associate with insurance coverage?

**Findings:**

In this cross-sectional study of age-specific Healthcare Access and Quality (HAQ) across the US, Canada, and 3 high-income regions with universal health insurance coverage (western Europe, high-income Asia Pacific, and Australasia), 2016 US national scores were lower than high-income peers with universal health insurance coverage among individuals of working ages between 15 and 64 years. Across US states in 2010 and 2016, age-specific HAQ scores were associated with insurance coverage for some working-age categories.

**Meaning:**

These findings suggest that personal HAQ is associated with insurance coverage.

## Teaser

This cross-sectional study compares age-specific health care access and quality scores for the US with high-income countries with universal health insurance coverage and compares scores among US states with varying insurance coverage.

## Introduction

Despite the contributions of the US to biology and medical science,^1,2^ the US health care system does not perform as well as most high-income countries according to various measures.^3,4,5,6^ The Healthcare Access and Quality (HAQ) index, created by Global Burden of Diseases, Injuries, and Risk Factors Study (GBD) collaborators, is based on amenable mortality, defined as deaths that should not occur in the presence of timely and effective care.^3^ According to the age-standardized HAQ index, the US ranked 29 of 195 countries in 2016 with a score of 88.7 (95% uncertainty interval [UI], 88.0-89.4).^6^ The US health care system serves some populations better than others. When comparing populations across states, the 2016 age-standardized HAQ scores ranged from a high in Minnesota of 92.3 (95% UI, 90.6-93.6) to a low in Mississippi of 81.5 (95% UI, 78.6-84.2).^6^ Health insurance coverage varies by age and state because state governments can expand benefits and eligibility for programs above the minimum federal requirements,^7,8,9,10^ with the exception of federally provided Medicare for individual aged 65 or older or individuals who are disabled and eligible for Social Security benefits or have end-stage kidney disease. In this study, our objective was to compare US age-specific HAQ scores with those of high-income countries with universal health insurance coverage and compare scores among US states with varying insurance coverage.

## Methods

### Reporting Guidelines

This cross-sectional study did not require ethical review by the University of Washington Human Subjects Division or informed consent because it used GBD results and US Census Bureau public-use data. This study followed the Strengthening the Reporting of Observational Studies in Epidemiology (STROBE) reporting guidelines.

### HAQ Index

Health care system performance is commonly measured at the population level by mortality among children ages 0 to 5 years and maternal mortality because these mortality rates are low in well-functioning health care systems. Beginning with Rutstein et al^11^ in 1976, researchers have sought additional measures that include more age categories, more causes, and a broader range of health services. Nolte and McKee’s^12^ list of amenable causes among ages 0 to 74 years is the most widely used and the basis of the HAQ index. The HAQ index combines mortality data from 32 amenable causes (eTable 1 in the Supplement).^5,6^ Examples of amenable causes are neonatal disorders, maternal disorders, and 4 vaccine preventable diseases, for which high-quality care affects mortality among children ages 0 to 5 years and maternal mortality; colon, rectum, and breast cancers, for which high-quality care includes screening and early detection as well as treatment; ischemic heart disease, cerebrovascular disease, and diabetes, for which high-quality care includes management of high systolic blood pressure or fasting plasma glucose in primary care; and appendicitis, for which high-quality care includes emergency surgical care.

The 2016 HAQ index was calculated with risk-standardized, 2016 GBD mortality rates for 24 amenable causes, in which risk-standardization controls for mortality differences across locations and years attributable to environmental and behavioral risk factors as opposed to personal health care.^5,6^ Risk standardization was based on 2016 GBD comparative risk assessment results.^13^ It also used 2016 GBD mortality-to-incidence ratios for 8 amenable neoplasms to more robustly reflect differences in access and quality of cancer care (eTable 1 in the Supplement).^6^ The GBD analysis provides mortality^14^ and incidence^15^ estimates by cause and age category, which are 5-year intervals covering ages 5 to 74 years, and 4 narrower categories for ages 0 to 4 years.

We created age-specific HAQ indices in 1990, 2000, 2010, and 2016 for this analysis that began with age-specific, risk-standardized mortality rates and mortality incidence ratios. The following 2 steps were performed separately for each age category. The cause-specific measures were log transformed and rescaled on a 0 to 100 scale, using the worst (first percentile) and best (99th percentile) mortality results within each age category observed across all countries from 1990 to 2016 to set the minimum and maximum, respectively. With secular improvements in health care access and quality, the worst mortality results for each cause and age category are generally from earlier years and the best are generally from later years.

Next, to combine the cause-specific scales for each age category, we calculated a weighted mean using the cause weights from the age-standardized 2016 HAQ index. These cause weights are estimated using principal components analysis from the age-standardized cause-specific measures across all health care systems globally (ie, 1 measure for each cause, location, and year). The cause weights reflect patterns of mortality across health care systems. In contrast, an index based on total amenable mortality^16^ would be weighted by the distribution of mortality in each location, for example, disproportionately weighting ischemic heart disease in the US. By using the same cause weights across all locations, the HAQ index isolates a measure of health care system performance.

However, some causes are not amenable or relevant for all age categories; for example, the amenable range for diarrheal diseases is 0 to 14 years, and maternal conditions are not relevant for girls ages 0 to 9 years. For each age category, the cause weights are rescaled to sum to 1 (eTable 2 in the Supplement).

The GBD results for mortality rates are 1000 draws from the posterior distribution of the cause-specific mortality rates for each age, sex, location, and year.^14^ We used 1000 draws from the GBD mortality results and 1000 draws from the GBD incidence results for neoplasms^15^ to construct the age-specific HAQ index and report the mean of 1000 draws for each age-specific HAQ index.

### American Community Survey

To estimate insurance coverage and the median income per person by age category and state, we used the American Community Survey (ACS) Public Use Microdata Sample (PUMS).^17^ The ACS is an annual survey of a sample of residential addresses selected from every county, and the surveys beginning in 2008 provide the most precise, available estimates of health insurance coverage at the state level and population subgroups at the national level.^17^ Respondents were asked about insurance coverage at the time of the interview for each member of their household or group quarters.^17^ The PUMS data covered more than one-third of the overall sample and represented 1% of the US population. To match the 2016 GBD results, we analyzed annual PUMS data for 2010, with samples of 2 769 241 people in households and 64 677 people in group quarters, and for 2016 with samples of 2 778 447 people in households and 124 644 people in group quarters. Sample person weights were applied to observations to ensure representativeness.

We defined total insurance coverage as coverage from 1 or more of the 6 sources in the ACS and calculated the percentage of the population with insurance coverage in the same 5-year intervals covering ages 5 to 74 years, and 2 categories for children aged 0 to 11 months and 1 to 4 years. ACS data were not disaggregated for children younger than 1 year. A state’s age-specific median income per person represented household income per person for people in households and personal income for individuals in group quarters.

### Statistical Analysis

We first estimated the age-specific HAQ index using national data on the US and high-income peer countries with universal health insurance coverage in 1990, 2000, 2010, and 2016 to identify relative levels, trends, and differences. Results are reported for the US, 3 high-income GBD regions with universal health insurance coverage (ie, western Europe, high-income Asia Pacific, and Australasia), and Canada. The Organization for Economic Cooperation and Development reports that 91.2% of the population in the US had insurance coverage in 2016, and 100% had it in the 3 GBD regions and Canada.^18^ The quality of death registration data in these locations is also high.^19^ We did not include the high-income Latin American region because available evidence showed that less than 100% of the population had insurance coverage in 2016, and countries did not report coverage by age. eTable 3 in the Supplement provides a list of countries in high-income GBD regions, the number of years with complete death registration data, data quality, and the percentage of the population with insurance.

The mean scores for each US state and the District of Columbia in 2016 were calculated to describe differences in age-specific HAQ scores by state. Details on national cause-specific mortality scores and the range of state scores for each amenable cause are reported in the eAppendix, eFigure 1, and eFigure 2 in the Supplement.

A multivariable regression analysis was conducted to analyze the association between the age-specific HAQ scores and insurance coverage at the state level in 2010 and 2016. The unit of analysis was the state-age-year with 1632 observations (51 states, 16 age categories, and 2 years). The dependent variable was the state’s mean age-specific HAQ score, and independent variables were the age-specific insurance coverage, age-specific median income per person, fixed effects for each age category, and year. Coefficients for insurance coverage with median income per person and year were estimated for each age category. Median income per person controlled for the financial resources available, which was similar to limiting the national comparison to high-income countries.

Pooled ordinary least-squares analyses were reported without and with state-fixed effects. Analyses without state-fixed effects estimated the association between HAQ scores and insurance coverage across states within age categories. If unmeasured state characteristics are associated with age-specific insurance coverage, insurance coefficients may be biased and their standard errors may be underestimated. Analysis with state-fixed effects controlled for unmeasured state characteristics. Results are presented with 99% CIs and based on a 1% level of significance to address multiple testing. We compared the outcome associated with a 1 SD change in total insurance coverage or median income per capita (eTable 4 in the Supplement). Sensitivity analyses were conducted with 2 state-level covariates that measure health care system infrastructure: hospital beds per 1000 population and physicians per 1000 population (eTable 5 and eTable 6 in the Supplement). A counterfactual analysis was conducted to calculate the increase in HAQ scores with universal insurance coverage for every state and each age category using the regression results.

The age-specific HAQ indices were constructed and national-level analyses were performed with Python statistical software version 3.0. (Python). The state-level analyses were performed with R statistical software version 3.6.3. (R Project for Statistical Computing).

## Results

### International and National Age-Specific HAQ Scores From 1990 to 2016

**Figure 1.**
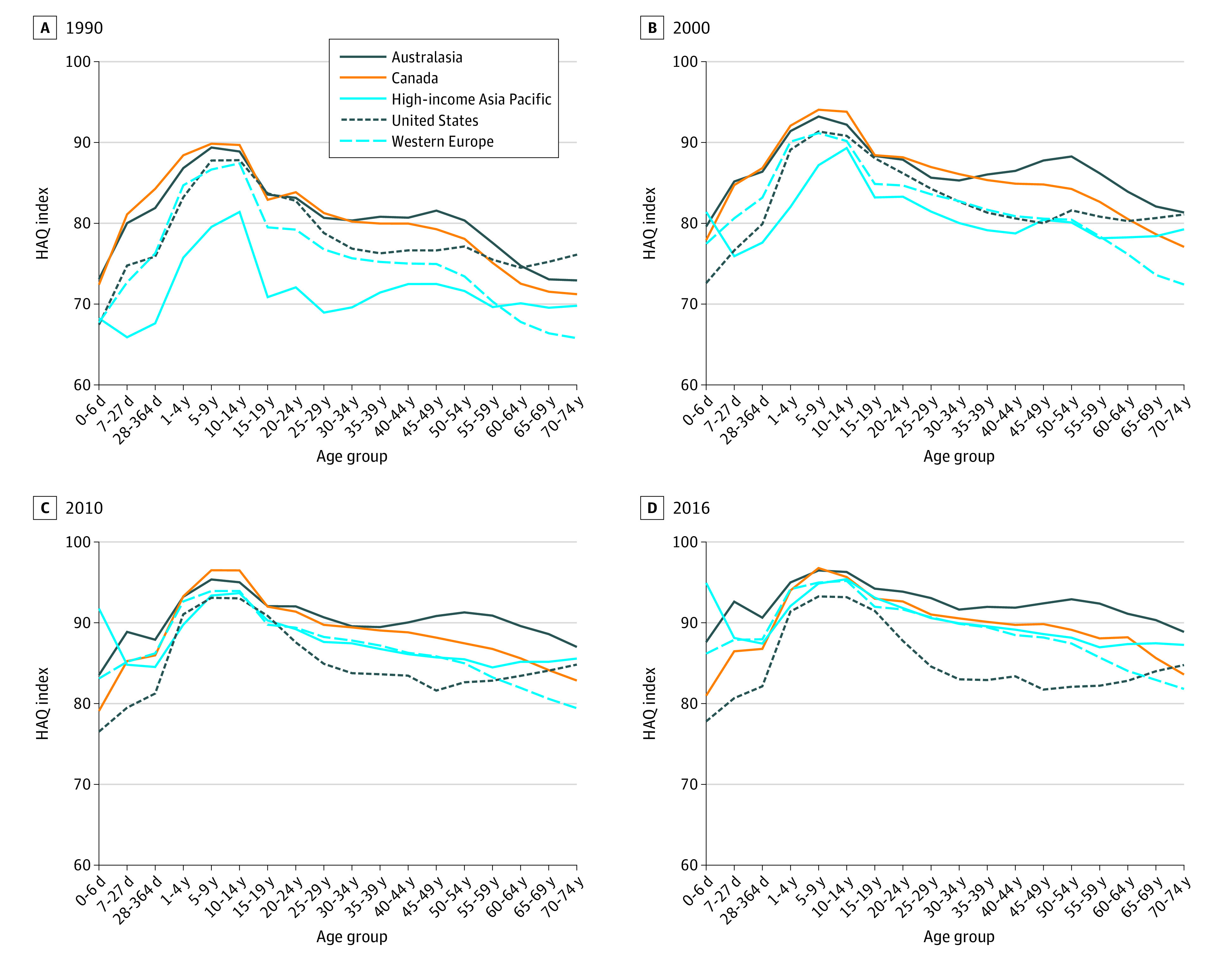
Age-Specific Healthcare Access and Quality (HAQ) Index for US and High-Income Peers With Universal Health Insurance in Selected Years

In 1990, the age pattern in the age-specific HAQ indices was similar for the US and high-income peers with universal health insurance coverage (Figure 1). Scores were low among children younger than 1 year, high for ages 5 to 9 years and 10 to 14 years and decreased with age beginning at ages 15 to 19 years. For example, for ages 20 to 24 years, the US score (82.8 points) was in the middle among high-income Asia Pacific (72.1 points), western Europe (79.2 points), Australasia (83.1 points), and Canada (83.8 points). Although the range was wider for ages 50 to 54 years, the US score (77.1 points) was in the middle among high-income Asia Pacific (71.6 points), western Europe (73.4 points), Canada (78.1 points), and Australasia (80.3 points). The US score was the lowest observed was ages 0 to 6 days (67.4 points) and highest observed for ages 15 to 19 years (83.7 points), 65 to 69 years (76.1 points), and 70 to 74 years (75.2 points).

No location had an HAQ score of 100 because the HAQ index was a weighted mean, and no location consistently had the lowest cause-specific mortality result for every cause. The regional results were population-weighted means across countries. Although 1 country may have had a relatively high HAQ score, the regional mean was lower.

Age-specific HAQ scores increased over time, but the US scores increased less for ages 5 years or older than high-income peer locations from 1990 to 2010 and did not change appreciably from 2010 to 2016. For example, for ages 20 to 24 years, HAQ scores increased 5.0 points (87.8 points) in the US from 1990 to 2016 compared with 19.7 points (91.8 points) in high-income Asia Pacific, 12.4 points (91.6 points) in western Europe, 10.7 points (93.8 points) in Australasia, and 8.8 points (92.6 points) in Canada. For ages 50 to 54 years, the HAQ scores increased 4.9 points (82.1 points) in the US, compared with 16.5 points (88.2 points) in high-income Asia Pacific, 14.0 points (87.4 points) in western Europe, 11.0 points (89.1 points) in Canada, and 12.6 points (92.9 points) in Australasia. Thus, the US ranked the lowest among its peers in every age category in 2016, with the exception ages 65 to 69 years and 70 to 74 years.

### Age-Specific HAQ Scores by US State in 2016

**Figure 2.**
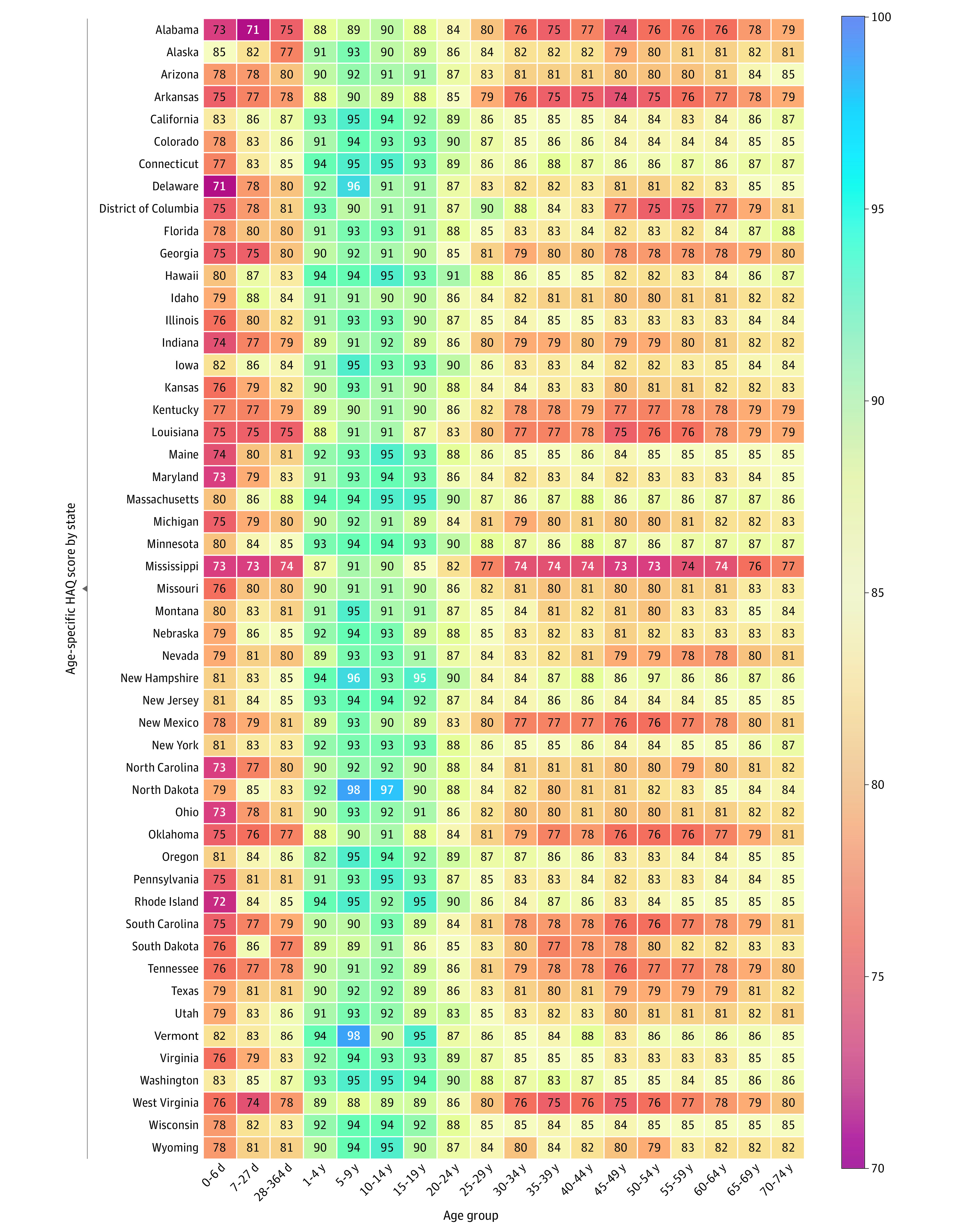
Age-Specific Healthcare Access and Quality (HAQ) Scores for US States in 2016

Although the US national age-specific HAQ scores were less than those at peer locations in 2016, HAQ scores in some states were comparable with peer locations. For example, the age-specific HAQ scores were 85 or greater for individuals ages 15 years or older in 3 states (ie, Connecticut, Massachusetts, and Minnesota), and 84 or greater in 8 states (Figure 2). In contrast, the age-specific HAQ scores were 75 or less for at least 1 age category between the ages of 15 to 64 years in 6 states (ie, Alabama, Arkansas, District of Columbia, Louisiana, Mississippi, and West Virginia). The ranges in age-specific HAQ scores across states were large; for example, it was 9.6 points for ages 15 to 19 years and 13.3 points for ages 60 to 64 years (eFigure 2 in the Supplement).

### Association Between Health Insurance Coverage and Age-Specific HAQ Index in the US 2010 and 2016

In the multivariable analysis of state-level data, the age-specific HAQ score was positively associated with insurance coverage for most working ages between 15 and 64 years (Table 1). For example, in pooled estimates without state-fixed effects, the coefficients were large and statistically significant for working ages, with 2 exceptions at ages 25 to 29 years and 55 to 59 years. A 10% absolute increase in insurance coverage was associated with an increase of 1.43 (99% CI, 0.48-2.37) points in HAQ score for ages 20 to 24 years, 2.95 (99% CI, 1.51-4.39) points for ages 45 to 49 years, and 2.51 points (99% CI, 0.90-4.11) for ages 50 to 54 years. In pooled estimates with state-fixed effects, the coefficients were smaller and statistically significant for 3 categories of working-age individuals: ages 20 to 24 years (0.059 [99% CI, 0.006-0.111]), 45 to 49 years (0.088 [99% CI, 0.009-0.167]), and 50 to 54 years (0.101 [99% CI, 0.013-0.189]). For these groups, a 10% absolute increase in insurance coverage was associated with an increase in HAQ scores of 0.59 (99% CI, 0.06-1.11) points for ages 20 to 24 years, 0.88 (99% CI, 0.09-1.67) points for ages 45 to 49 years, and 1.01 (99% CI, 0.13-1.89) points for ages 50 to 54 years.

**Table 1.** Association Between HAQ Scores and Total Health Insurance Coverage by Age Category in the United States, 2010 and 2016^a^

Age category, y	Age-specific total insurance coverage		Age-specific median income per capita (thousands), $		Year	
	Coefficient for a 1% change in insurance coverage (99% CI)	*P* value	Coefficient for a $1000 increase in median income per capita (99% CI)	*P* value	Coefficient for the change from 2010 to 2016 (99% CI)	*P* value
**Model with age fixed effects** ^b^						
0-11 mo	−0.049 (−0.359 to 0.261)	.68	0.535 (0.347 to 0.724)	<.001	−0.808 (−2.130 to 0.514)	.12
1-4	0.094 (−0.132 to 0.321)	.28	0.425 (0.221 to 0.628)	<.001	−1.230 (−2.613 to 0.153)	.02
5-9	−0.015 (−0.236 to 0.207)	.86	0.421 (0.190 to 0.652)	<.001	−0.212 (−1.537 to 1.112)	.68
10-14	−0.132 (−0.351 to 0.088)	.12	0.453 (0.236 to 0.671)	<.001	−0.302 (−1.695 to 1.091)	.58
15-19	0.188 (0.048 to 0.328)	.001	0.317 (0.098 to 0.536)	<.001	−1.044 (−2.594 to 0.506)	.08
20-24	0.143 (0.048 to 0.237)	<.001	0.233 (−0.008 to 0.474)	.01	−3.246 (−5.144 to −1.348)	<.001
25-29	0.104 (−0.016 to 0.225)	.02	0.352 (0.171 to 0.533)	<.001	−3.256 (−4.920 to −1.591)	<.001
30-34	0.197 (0.076 to 0.318)	<.001	0.225 (0.096 to 0.354)	<.001	−3.830 (−5.346 to −2.313)	<.001
35-39	0.250 (0.125 to 0.376)	<.001	0.155 (0.032 to 0.277)	.001	−2.968 (−4.382 to −1.554)	<.001
40-44	0.264 (0.128 to 0.400)	<.001	0.236 (0.063 to 0.408)	<.001	−2.474 (−3.860 to −1.088)	<.001
45-49	0.295 (0.151 to 0.439)	<.001	0.219 (0.049 to 0.389)	.001	−3.103 (−4.567 to −1.640)	<.001
50-54	0.251 (0.090 to 0.411)	<.001	0.369 (0.204 to 0.535)	<.001	−3.565 (−5.001 to −2.130)	<.001
55-59	0.125 (−0.060 to 0.310)	.08	0.479 (0.329 to 0.628)	<.001	−2.493 (−3.891 to −1.094)	<.001
60-64	0.226 (0.017 to 0.435)	.01	0.401 (0.255 to 0.546)	<.001	−2.655 (−4.069 to −1.240)	<.001
65-69	0.372 (−0.351 to 1.094)	.18	0.451 (0.295 to 0.606)	<.001	−2.186 (−3.643 to −0.728)	<.001
70-74	−0.401 (−1.396 to 0.595)	.30	0.273 (0.105 to 0.440)	<.001	−1.258 (−2.779 to 0.263)	.03
**Model with age and state fixed-effects** ^c^						
0-11 mo	−0.008 (−0.173 to 0.157)	.90	0.089 (−0.018 to 0.196)	.03	0.580 (−0.121 to 1.282)	.03
1-4	0.080 (−0.042 to 0.201)	.09	−0.085 (−0.201 to 0.032)	.06	0.404 (−0.337 to 1.145)	.16
5-9	0.021 (−0.098 to 0.140)	.65	−0.235 (−0.367 to −0.103)	<.001	0.991 (0.291 to 1.692)	<.001
10-14	−0.069 (−0.187 to 0.05)	.13	−0.160 (−0.283 to −0.037)	.001	1.025 (0.282 to 1.768)	<.001
15-19	0.034 (−0.045 to 0.113)	.27	−0.154 (−0.273 to −0.034)	.001	1.193 (0.352 to 2.034)	<.001
20-24	0.059 (0.006 to 0.111)	.004	−0.241 (−0.373 to −0.109)	<.001	−0.072 (−1.127 to 0.984)	.86
25-29	0.005 (−0.060 to 0.070)	.84	0.160 (0.060 to 0.260)	<.001	−1.205 (−2.104 to −0.305)	.001
30-34	0.004 (−0.063 to 0.070)	.90	0.214 (0.143 to 0.284)	<.001	−1.92 (−2.737 to −1.104)	<.001
35-39	0.026 (−0.044 to 0.095)	.34	0.167 (0.100 to 0.233)	<.001	−1.402 (−2.160 to −0.645)	<.001
40-44	0.057 (−0.017 to 0.132)	.05	0.184 (0.089 to 0.279)	<.001	−0.984 (−1.727 to −0.242)	.001
45-49	0.088 (0.009 to 0.167)	.004	0.112 (0.019 to 0.205)	.002	−1.208 (−2.002 to −0.414)	<.001
50-54	0.101 (0.013 to 0.189)	.003	0.117 (0.025 to 0.209)	.001	−1.78 (−2.553 to −1.007)	<.001
55-59	0.039 (−0.063 to 0.140)	.33	0.154 (0.071 to 0.237)	<.001	−1.192 (−1.937 to −0.446)	<.001
60-64	0.113 (−0.002 to 0.228)	.01	0.095 (0.013 to 0.176)	.003	−1.302 (−2.058 to −0.546)	<.001
65-69	0.123 (−0.265 to 0.511)	.41	0.071 (−0.020 to 0.161)	.04	−0.290 (−1.074 to 0.494)	.34
70-74	−0.635 (−1.164 to −0.106)	.002	0.020 (−0.074 to 0.114)	.59	0.113 (−0.699 to 0.924)	.72

Abbreviation: HAQ, Healthcare Access and Quality.

^a^ Results of multivariable regression analysis of age-specific HAQ scores as the dependent variable, and age-specific health insurance coverage, age-specific median income per capita and year. All regression models have age fixed-effects and age interaction for insurance coverage, median income per capita, and year. To address multiple testing, results presented with 99% CI.

^b^ The mean square error is 6.05 and square root of mean square error is 2.46.

^c^ The mean square error is 1.63 and square root of mean square error is 1.28.

**Figure 3.**
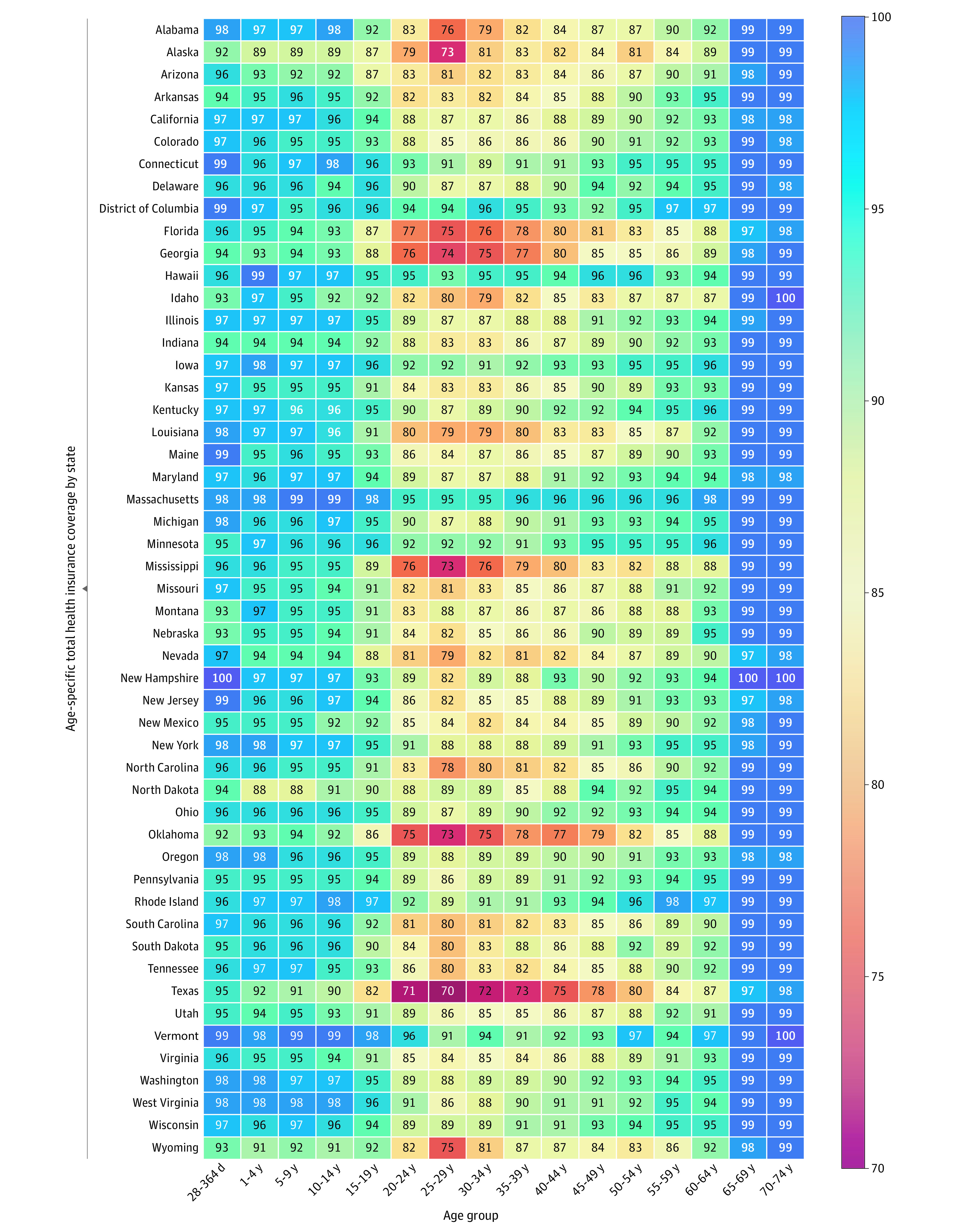
Total Health Insurance Coverage by Age Category for US States in 2016 Abbreviation: HAQ, healthcare and quality index.

The increases in HAQ scores associated with universal health insurance coverage were calculated as the product of the insurance gap and the regression coefficients (Figure 3). In 2016, using pooled estimates without state-fixed effects, universal health insurance coverage would increase HAQ scores by at least 3 points for ages 40 to 44 in 30 of 50 states and the District of Columbia (Table 2; eTable 7 in the Supplement). Using pooled estimates with state-fixed effects, HAQ scores would increase by at least 1 point for ages 20 to 24 years in 13 states, ages 45 to 49 years in 21 states, ages 50 to 54 years in 23 states, and ages 60 to 64 years in 9 states.

**Table 2.** Summary of Counterfactual Estimates of the Increase in HAQ Score in US States With Universal Health Insurance Coverage, 2010 and 2016^a^

Age category, y	Model with age fixed effects				Model with age and state fixed effects			
	Largest increase in HAQ points among states	States with increase in HAQ points, No.			Largest increase in HAQ points among states	States with increase in HAQ points, No.		
		>1.0	>3.0	>5.0		>1.0	>2.0	>3.0
**2016**								
0-11 mo	0	0	0	0	0.0	0	0	0
1-4	1.1	2	0	0	0.9	0	0	0
5-9	0.0	0	0	0	0.2	0	0	0
10-14	−0.1	0	0	0	−0.1	0	0	0
15-19	3.4	33	1	0	0.6	0	0	0
20-24	4.1	46	5	0	1.7	13	0	0
25-29	3.0	44	1	0	0.1	0	0	0
30-34	5.5	48	21	1	0.1	0	0	0
35-39	6.6	50	29	5	0.7	0	0	0
40-44	6.5	50	30	5	1.4	6	0	0
45-49	6.3	50	26	4	1.9	21	0	0
50-54	4.8	47	14	0	1.9	23	0	0
55-59	2.0	22	0	0	0.6	0	0	0
60-64	2.9	42	0	0	1.5	9	0	0
65-69	0.9	0	0	0	0.3	0	0	0
70-74	0	0	0	0	0.0	0	0	0
**2010**								
0-11 mo	0.0	0	0	0	0.0	0	0	0
1-4	1.6	5	0	0	1.3	2	0	0
5-9	0.0	0	0	0	0.4	0	0	0
10-14	−0.2	0	0	0	−0.1	0	0	0
15-19	5.1	49	18	1	0.9	0	0	0
20-24	6.4	51	46	14	2.6	47	17	0
25-29	4.4	51	25	0	0.2	0	0	0
30-34	7.5	51	47	20	0.1	0	0	0
35-39	8.4	51	47	30	0.9	0	0	0
40-44	8.2	51	47	22	1.8	27	0	0
45-49	8.4	51	47	25	2.5	44	11	0
50-54	6.3	51	38	9	2.5	47	10	0
55-59	2.6	47	0	0	0.8	0	0	0
60-64	4.5	50	11	0	2.3	34	1	0
65-69	2.0	6	0	0	0.7	0	0	0
70-74	0.0	0	0	0	0.0	0	0	0

Abbreviation: HAQ, Healthcare Access and Quality.

^a^ The potential increase in HAQ scores is calculated with the estimated coefficients for total health insurance coverage and gap between the state’s coverage and 100% coverage.

## Discussion

This cross-sectional study found that when health care system performance was measured at the population level with an age-specific HAQ index, the US national scores increased from 1990 to 2010 across age categories. However, the increase was less than those of high-income peers with universal health insurance coverage and did not change between 2010 and 2016. Using state-level age-specific HAQ scores, we identified several states with scores in 2016 that were comparable with high-income peers and large differences in performance across states. Analyzing these state-level results, we found that the age-specific HAQ scores were associated with insurance coverage in some working-age categories.

Our comparison of national results for the age-specific HAQ indices is consistent with previous research on amenable mortality in the US and adds insights into the US performance during working ages. The absence of an increase in HAQ scores between 2010 and 2016 is consistent with the Commonwealth Fund’s report^16^ that the total mortality rate from amenable causes declined at a slower rate from 2010 and 2011 to 2012 and 2013 than from 2004 and 2005 to 2010 and 2011 and increased from 83.7 per 100 000 population in 2012 and 2013 to 84.3 in 2013 and 2014. Nolte and McKee^20^ reported that the age-standardized amenable mortality rate for ages 0 to 64 years was high in the US relative to France, Germany, and the United Kingdom from 1999 to 2006 and 2007. Mortality rates for ages 65 to 74 years were similar in the US, Germany, and the United Kingdom in 2006 and 2007 and higher than in France.

In the international comparisons, the US health care system performed poorly for working ages in recent years, and this finding was consistent with lower insurance coverage for these age groups in the US. In the comparison among states, total insurance coverage and the median income per person were associated with increased HAQ scores for working-aged individuals; increases in coverage and income were associated with offsetting a negative time trend between 2010 and 2016. However, total insurance coverage may be confounded with other state-level insurance policies, such as the benefits package, continuity of coverage, and cost-sharing, or state-level employment policies, such as paid leave for medical care. The association between the HAQ scores and total insurance coverage suggests that further research on the effects of other state-level insurance and employment policies is warranted.

Other potential reasons why health care system performance would differ across age categories or states in the US were addressed in our estimates. For example, environmental, occupational, and behavioral risk factors differed across ages, states, and years, and we controlled for these risks in our analysis by calculating the age-specific HAQ index with risk-standardized mortality rates. Also, health infrastructure, such as hospitals or physicians per 1000 population, differed across states and years but not ages. In estimates that included these state-level variables, the coefficient estimates for insurance coverage were similar or larger than estimates without state-level variables. These state-level variables did not improve model fit in our sample with 2 years of data and estimates with state fixed effects.

Other possible factors that could contribute to health care system performance differences, which we did not address in this study, are extended office hours or location of health facilities close to populations with inflexible work hours or without sick leave. However, an analysis of these factors would require local-level data, and measures of insurance coverage by 5-year age categories are unavailable at the local level.

To our knowledge, this is the first study to explore the association between health insurance coverage and US HAQ scores across multiple age categories at the state level. Although our analysis focuses on health care system performance, it may have implications for research on the effects of insurance coverage on health. Although the effects of health insurance on health are unclear, a review by Levy and Metzler^21^ concluded that health insurance improves health for populations such as infants, children, and people with AIDS or high blood pressure. A review by Dor and Umapathi^22^ recommended further research on which patient populations would benefit from insurance coverage. There is evidence that insurance coverage reduces mortality for infants,^23^ young children,^24^ people with HIV,^25,26^ hospital patients admitted from an emergency department,^27^ and people with end-stage kidney disease.^28^ Health insurance may be more likely to affect amenable causes and benefit populations without access to high-quality care for those causes. Future researchers could test the outcomes associated with insurance coverage in the US with appropriately weighted mortality or disability-adjusted life-years for amenable causes in age categories with the largest ranges in HAQ scores.

### Limitations

This study had limitations. Amenable mortality, as defined by Nolte and McGee^3^ ends at age 74 years, which was adopted for the HAQ index. Since 2003, advances in health care for older individuals may have extended amenable mortality to ages 75 years or older for some causes. Similarly, amenable mortality combines results for both sexes, which limits the understanding of potential sex-specific differences in access and quality; future research could use GBD mortality results for each sex to calculate sex-specific and age-specific HAQ indices.

The HAQ index was calculated with only the principal component analysis (PCA) weights from the age-standardized HAQ. In the future, it would be possible to estimate the age-specific HAQ indices with both PCA and arithmetic mean to determine whether the method of combining the cause-specific mortality rates altered the comparison among US states and between the US and peer locations. Furthermore, the international comparison was not included, and socioeconomic characteristics such as education, racial and ethnic discrimination, and immigration status, were not controlled for in the regression analysis. Our estimates controlled for age-specific median income per person but did not compare HAQ scores between income categories across states. Chen et al^29^ show differences in mortality among children aged 2 to 12 months across US regions among disadvantaged groups, but not among advantaged groups. The ACS sample size is designed for state-level analyses or national-level subgroup analyses but not for state-level subgroup analyses. Although statistically significant associations were found, our cross-sectional study was limited by its design and did not determine causation.

## Conclusions

In this cross-sectional study, the 2016 US age-specific HAQ scores for working individuals ages 15 to 64 years were low compared with high-income global peers with universal health insurance coverage. Among US states, insurance coverage was associated with higher HAQ scores for some working ages between 15 and 64 years. Further research with causal models and additional explanations is warranted.

## Notes

Supplement. **eTable 1.** Amenable Causes in the 2016 GBD HAQ Index, and Risk Factors Associated With Them **eTable 2.** Age Category for Each GBD Cause of Death Included in the HAQ Index, and Rescaled Cause Weights for Each Age Category **eTable 3.** High-Income Countries by GBD Super-Region, Region, and Health Insurance Coverage **eAppendix.** Cause-Specific Mortality Scores by Age Category in the US in 2016 **eFigure 1.** Age-Specific HAQ Index and Cause-Specific Mortality Scores for the US in 2016 **eFigure 2.** Range in Age-Specific HAQ Index and Cause-Specific Mortality Scores Across US States in 2016 **eTable 4.** Comparison of Estimated Coefficients Multiplied by SD for Age-Specific Total Insurance Coverage and Median Income Per Capita by Age Category in the United States, 2016 **eTable 5.** Association Between HAQ Scores and Total Health Insurance Coverage by Age Category in the United States, 2010 and 2016; Sensitivity Analysis With State-Level Variable for Hospital Beds Per 1000 Population **eTable 6.** Association Between HAQ Scores and Total Health Insurance Coverage by Age Category in the United States, 2016; Sensitivity Analysis With State-Level Variable for Physicians Per 1000 Population **eTable 7.** Detailed Counterfactual Estimates of the Increase in HAQ Score With Universal Health Insurance Coverage by State and Age Category, 2010 and 2016
