# Error in Figure 3

**DOI:** 10.1001/jamanetworkopen.2021.25050

**Published:** 2021-08-06

In the Original Investigation titled “Variation in Health Care Access and Quality Among US States and High-Income Countries With Universal Health Insurance Coverage,”^1^ published June 28, 2021, there was an error in a column label of Figure 3. The label has been updated from 28 to 364 days to 0 to 11 months. This article has been corrected.^1^

## References

[1] Weaver MR, Nandakumar V, Joffe J, . Variation in health care access and quality among US states and high-income countries with universal health insurance coverage. JAMA Netw Open. 2021;4(6):e2114730. doi:10.1001/jamanetworkopen.2021.14730 34181011 PMC9434824

