# Supplementary material for: Variation in Health Care Access and Quality Among US States and High-Income Countries With Universal Health Insurance Coverage
Source: JAMA Netw Open. 2021 Jun 28;4(6):e2114730. doi: 10.1001/jamanetworkopen.2021.14730 (PMC9434824; doi:10.1001/jamanetworkopen.2021.14730)
Supplement: Supplement. — eTable 1. Amenable Causes in the 2016 GBD HAQ Index, and Risk Factors Associated With Them eTable 2. Age Category for Each GBD Cause of Death Included in the HAQ Index, and Rescaled Cause Weights for Each Age Category eTable 3. High-Income Countries by GBD Super-Region, Region, and Health Insurance Coverage eAppendix. Cause-Specific Mortality Scores by Age Category in the US in 2016 eFigure 1. Age-Specific HAQ Index and Cause-Specific Mortality Scores for the US in 2016 eFigure 2. Range in Age-Specific HAQ Index and Cause-Specific Mortality Scores Across US States in 2016 eTable 4. Comparison of Estimated Coefficients Multiplied by SD for Age-Specific Total Insurance Coverage and Median Income Per Capita by Age Category in the United States, 2016 eTable 5. Association Between HAQ Scores and Total Health Insurance Coverage by Age Category in the United States, 2010 and 2016; Sensitivity Analysis With State-Level Variable for Hospital Beds Per 1000 Population eTable 6. Association Between HAQ Scores and Total Health Insurance Coverage by Age Category in the United States, 2016; Sensitivity Analysis With State-Level Variable for Physicians Per 1000 Population eTable 7. Detailed Counterfactual Estimates of the Increase in HAQ Score With Universal Health Insurance Coverage by State and Age Category, 2010 and 2016 [file jamanetwopen-e2114730-s001.pdf]

Supplemental Online Content

Weaver MR, Nandakumar V, Joffe J, et al. Variation in health care access and quality among US states and high-income countries with universal health insurance coverage. *JAMA Netw Open*. 2021;4(6):2114730. doi:10.1001/jamanetworkopen.2021.14730

eTable 1. Amenable Causes in the 2016 GBD HAQ Index, and Risk Factors Associated With Them

eTable 2. Age Category for Each GBD Cause of Death Included in the HAQ Index, and Rescaled Cause Weights for Each Age Category

eTable 3. High-Income Countries by GBD Super-Region, Region, and Health Insurance Coverage

eAppendix. Cause-Specific Mortality Scores by Age Category in the US in 2016

eFigure 1. Age-Specific HAQ Index and Cause-Specific Mortality Scores for the US in 2016

eFigure 2. Range in Age-Specific HAQ Index and Cause-Specific Mortality Scores Across US States in 2016

eTable 4. Comparison of Estimated Coefficients Multiplied by SD for Age-Specific Total Insurance Coverage and Median Income Per Capita by Age Category in the United States, 2016

eTable 5. Association Between HAQ Scores and Total Health Insurance Coverage by Age Category in the United States, 2010 and 2016; Sensitivity Analysis With State-Level Variable for Hospital Beds Per 1,000 Population

eTable 6. Association Between HAQ Scores and Total Health Insurance Coverage by Age Category in the United States, 2016; Sensitivity Analysis With State-Level Variable for Physicians Per 1000 Population

eTable 7. Detailed Counterfactual Estimates of the Increase in HAQ Score With Universal Health Insurance Coverage by State and Age Category, 2010 and 2016

This supplemental material has been provided by the authors to give readers additional information about their work.

© 2021 Weaver MR et al. *JAMA Network Open*.

eTable 1. Amenable Causes in the 2016 GBD HAQ Index, and Risk Factors Associated With Them

	Number of risk factors	Risk factors*
Tuberculosis	3	high systolic blood pressure,* smoking, alcohol use
Diarrhoeal diseases	10	non-exclusive breastfeeding, discontinued breastfeeding, child underweight, child wasting, child stunting, vitamin A deficiency, zinc deficiency, unsafe water source, unsafe sanitation, no access to handwashing facility
Lower respiratory infections	13	ambient particulate matter pollution, household air polution from solid fuels, non-exclusive breastfeeding, child underweight, child wasting, child stunting, short gestation for birthweight, low birthweight for gestation, zinc deficiency, second-hand smoke, alcohol use, no access to handwashing facility
Upper respiratory infections	2	short gestation for birthweight, low birthweight for gestation
Diphtheria	0	
Whooping cough	0	
Tetanus	0	
Measles	4	child underweight, child wasting, child stunting, vitamin A deficiency
Maternal disorders	1	interpersonal violence
Neonatal disorders	2	short gestation for birthweight, low birthweight for gestation
Colon and rectum cancer	10	Mortality incidence ratios for neoplasms were not risk adjusted.
Non-melanoma skin cancer	0	Mortality incidence ratios for neoplasms were not risk adjusted.
Breast cancer	4	Mortality incidence ratios for neoplasms were not risk adjusted.
Cervical cancer	1	Mortality incidence ratios for neoplasms were not risk adjusted.
Uterine cancer		Mortality incidence ratios for neoplasms were not risk adjusted.
Testicular cancer	0	Mortality incidence ratios for neoplasms were not risk adjusted.
Hodgkin's lymphoma	0	Mortality incidence ratios for neoplasms were not risk adjusted.
Leukaemia		Mortality incidence ratios for neoplasms were not risk adjusted.
Rheumatic heart disease	2	high systolic blood pressure*
Ischaemic heart disease	21	ambient particulate matter pollution, household air pollution from solid fuels, smoking, second-hand smoke, alcohol use, diet low in fruits, diet low in vegetables, diet low in legumes, diet low in whole grains, diet low in nuts and seeds, diet high in processed meats, diet low in fiber, diet low in seafood omega 3 fatty acids, diet low in polysaturated fatty acids, diet high in trans fatty acids, low physical activity, high

© 2021 Weaver MR et al. *JAMA Network Open*.

	Number of risk factors	Risk factors*
		fasting plasma glucose,* high total cholesterol,* high systolic blood pressure,* high body-mass index (adult), impaired kidney function
Cerebrovascular disease	13	ambient particulate matter pollution, household air pollution from solid fuels, smoking, second-hand smoke, alcohol use, diet low in fruits, diet low in vegetables, diet low in whole grains, low physical activity, high fasting plasma glucose,* high total cholesterol,* high systolic blood pressure,* high body-mass index (adult)
Hypertensive heart disease	2	alcohol use, high body-mass index (adult)
Chronic respiratory diseases		ambient particulate matter pollution, household air pollution from solid fuels, occupational exposure to asbestos, occupational exposure to silica, smoking, second hand smoke, high body-mass index (adult), high body-mass index (child)
Peptic ulcer disease	0	
Appendicitis	0	
Inguinal, femoral, and abdominal hernia	0	
Gallbladder and biliary diseases	0	
Epilepsy	1	alcohol use
Diabetes	14	ambient particulate matter pollution, household air pollution from solid fuels, smoking, secondhand smoke, alcohol use, diet low in fruits, diet low in whole grains, diet low in nuts and seeds, diet high in red meat, diet high in processed meat, diet high in sugar-sweetened beverages, low physical activity, high fasting plasma glucose,* high body-mass index (adult)
Chronic kidney disease	5	high fasting plasma glucose,* high systolic blood pressure,* high body-mass index (adult), sugar-sweetened beverages, salt
Congenital heart anomalies	0	
Adverse effects of medical treatment	0	

*Mortality rates were not adjusted for three amenable, metabolic risk factors: high fasting plasma glucose, high systolic blood pressure, and high total cholesterol.

© 2021 Weaver MR et al. *JAMA Network Open*.

eTable 2. Age Category for Each GBD Cause of Death Included in the HAQ Index, and Rescaled Cause Weights for Each Age Category

					**Rescaled weights**																	
	**GBD cause**	**Amen-able age range**	**Age range with GBD results**	**GBD 2016 HAQ cause weight**	**0 to 6 days**	**1 to 4 weeks**	**2 to 11 months**	**1 to 4 years**	**5 to 9 years**	**10 to 14 years**	**15 to 19 years**	**20 to 24 years**	**25 to 29 years**	**30 to 34 years**	**35 to 39 years**	**40 to 44 years**	**45 to 49 years**	**50 to 54 years**	**55 to 59 years**	**60 to 64 years**	**65 to 69 years**	**70 to 74 years**
1	Tuberculosis	0-74	0.1-95	0.0435			0.0921	0.0596	0.0627	0.0582	0.0559	0.0559	0.0559	0.0559	0.0559	0.0559	0.0610	0.0623	0.0674	0.0693	0.0693	0.0698
2	Diarrhoeal diseases	0-14	0-95	0.0475	0.1904	0.1904	0.1006	0.0651	0.0684	0.0635												
3	Lower respiratory infections	0-74	0-95	0.0333	0.1335	0.1335	0.0705	0.0456	0.0480	0.0445	0.0428	0.0428	0.0428	0.0428	0.0428	0.0428	0.0467	0.0477	0.0516	0.0531	0.0531	0.0534
4	Upper respiratory infections	0-74	0-95	0.0172	0.0689	0.0689	0.0364	0.0236	0.0248	0.0230	0.0221	0.0221	0.0221	0.0221	0.0221	0.0221	0.0241	0.0246	0.0267	0.0274	0.0274	0.0276
5	Diphtheria	0-74	0.1-55	0.0173			0.0366	0.0237	0.0249	0.0231	0.0222	0.0222	0.0222	0.0222	0.0222	0.0222	0.0242	0.0248	0.0268			
6	Whooping cough	0-14	0.1-55	0.0498			0.1055	0.0682	0.0717	0.0666												
7	Tetanus	0-74	0-95	0.0452	0.1812	0.1812	0.0957	0.0619	0.0651	0.0605	0.0581	0.0581	0.0581	0.0581	0.0581	0.0581	0.0633	0.0647	0.0701	0.0720	0.0720	0.0725
8	Measles	1-14	0.1-55	0.0556				0.0761	0.0801	0.0744												
9	Maternal disorders	0-74	10.0-50	0.0534						0.0714	0.0686	0.0686	0.0686	0.0686	0.0686	0.0686	0.0748	0.0765				
10	Neonatal disorders	0-74	0-1	0.0359	0.1439	0.1439	0.0760	0.0492														
11	Non-melanoma skin cancer	0-74	15-95	0.0233							0.0299	0.0299	0.0299	0.0299	0.0299	0.0299	0.0327	0.0334	0.0361	0.0371	0.0371	0.0374
12	Breast cancer	0-74	15-95	0.0397							0.0510	0.0510	0.0510	0.0510	0.0510	0.0510	0.0556	0.0568	0.0616	0.0632	0.0632	0.0637
13	Cervical cancer	0-74	15-95	0.0290							0.0373	0.0373	0.0373	0.0373	0.0373	0.0373	0.0406	0.0415	0.0450	0.0462	0.0462	0.0465
14	Uterine cancer	0-44	15-95	0.0442							0.0568	0.0568	0.0568	0.0568	0.0568	0.0568						
15	Colon and rectum cancer	0-74	15-95	0.0388							0.0498	0.0498	0.0498	0.0498	0.0498	0.0498	0.0544	0.0556	0.0602	0.0618	0.0618	0.0623
16	Testicular cancer	0-74	15-95	0.0415							0.0533	0.0533	0.0533	0.0533	0.0533	0.0533	0.0582	0.0594	0.0643	0.0661	0.0661	0.0666
17	Hodgkin lymphoma	0-74	0-95	0.0330			0.0699	0.0452	0.0475	0.0441	0.0424	0.0424	0.0424	0.0424	0.0424	0.0424	0.0463	0.0473	0.0512	0.0526	0.0526	0.0530
18	Leukaemia	0-44	0-95	0.0208			0.0441	0.0285	0.0300	0.0278	0.0267	0.0267	0.0267	0.0267	0.0267	0.0267						
19	Rheumatic heart disease	0-74	1.0-95	0.0278				0.0381	0.0400	0.0372	0.0357	0.0357	0.0357	0.0357	0.0357	0.0357	0.0390	0.0398	0.0431	0.0443	0.0443	0.0446
20	Ischaemic heart disease	0-74	0.1-95	0.0012			0.0025	0.0016	0.0017	0.0016	0.0015	0.0015	0.0015	0.0015	0.0015	0.0015	0.0017	0.0017	0.0019	0.0019	0.0019	0.0019
21	Cerebrovascular disease	0-74	0.0-95	0.0194	0.0778	0.0778	0.0411	0.0266	0.0279	0.0259	0.0249	0.0249	0.0249	0.0249	0.0249	0.0249	0.0272	0.0278	0.0301	0.0309	0.0309	0.0311
22	Hypertensive heart disease	0-74	0.1-95	0.0202			0.0428	0.0277	0.0291	0.0270	0.0259	0.0259	0.0259	0.0259	0.0259	0.0259	0.0283	0.0289	0.0313	0.0322	0.0322	0.0324
23	Chronic respiratory diseases	1-14	0.0-95	0.0328				0.0449	0.0472	0.0439												
24	Peptic ulcer disease	0-74	1.0-95	0.0324				0.0444	0.0467	0.0433	0.0416	0.0416	0.0416	0.0416	0.0416	0.0416	0.0454	0.0464	0.0502	0.0516	0.0516	0.0520
25	Appendicitis	0-74	1.0-95	0.0423				0.0579	0.0609	0.0566	0.0543	0.0543	0.0543	0.0543	0.0543	0.0543	0.0593	0.0606	0.0656	0.0674	0.0674	0.0679
26	Inguinal, femoral, and abdominal	0-74	1.0-95	0.0372				0.0509	0.0536	0.0498	0.0478	0.0478	0.0478	0.0478	0.0478	0.0478	0.0521	0.0533	0.0577	0.0593	0.0593	0.0597
27	Gallbladder and biliary diseases	0-74	1.0-95	0.0300				0.0411	0.0432	0.0401	0.0385	0.0385	0.0385	0.0385	0.0385	0.0385	0.0420	0.0430	0.0465	0.0478	0.0478	0.0481
28	Epilepsy	0-74	0.1-95	0.0241			0.0510	0.0330	0.0347	0.0322	0.0310	0.0310	0.0310	0.0310	0.0310	0.0310	0.0338	0.0345	0.0374	0.0384	0.0384	0.0387
29	Diabetes mellitus	0-49	0-95	0.0151	0.0605	0.0605	0.0320	0.0207	0.0217	0.0202	0.0194	0.0194	0.0194	0.0194	0.0194	0.0194	0.0212					
30	Chronic kidney disease	0-74	0.1-95	0.0127			0.0269	0.0174	0.0183	0.0170	0.0163	0.0163	0.0163	0.0163	0.0163	0.0163	0.0178	0.0182	0.0197	0.0202	0.0202	0.0204
31	Congenital heart anomalies	0-74	0-65	0.0045	0.0180	0.0180	0.0095	0.0062	0.0065	0.0060	0.0058	0.0058	0.0058	0.0058	0.0058	0.0058	0.0063	0.0064	0.0070	0.0072	0.0072	
32	Adverse effects of medical	0-74	0-95	0.0314	0.1259	0.1259	0.0665	0.0430	0.0452	0.0420	0.0403	0.0403	0.0403	0.0403	0.0403	0.0403	0.0440	0.0450	0.0487	0.0500	0.0500	0.0504
	Variance explained			1.0001	1.0000	1.0000	1.0000	1.0000	1.0000	1.0000	1.0000	1.0000	1.0000	1.0000	1.0000	1.0000	1.0000	1.0000	1.0000	1.0000	1.0000	1.0000

© 2021 Weaver MR et al. *JAMA Network Open*.

eTable 3. High-Income Countries by GBD Super-Region, Region, and Health Insurance Coverage

Location	ISO code	Region	Number of years from 1990 to 2016 with complete death registration[1]	Data quality rating from 2010-2016[2]	Percentage of population with health insurance coverage in 2016[3]
Australia	AUS	Australasia	25	5	100
New Zealand	NZL	Australasia	25	5	100
Brunei	BRN	High-income Asia Pacific	24	4	--
Japan	JPN	High-income Asia Pacific	25	4	100
Singapore	SGP	High-income Asia Pacific	25	5	"universal"[4]
South Korea	KOR	High-income Asia Pacific	18	4	100
Canada	CAN	High-income North America	27	5	100
Greenland	GRL	High-income North America	22	5	100[5]
United States	USA	High-income North America	26	5	91.2
Argentina	ARG	High-income Latin America	25	4	--
Chile	CHL	High-income Latin America	25	4	93.1
Uruguay	URY	High-income Latin America	24	4	--
Andorra	AND	Western Europe	0	0	--
Austria	AUT	Western Europe	25	5	99.9
Belgium	BEL	Western Europe	25	4	99
Cyprus	CYP	Western Europe	21	4	--
Denmark	DNK	Western Europe	25	4	100
England	ENG	Western Europe	24	5	100
Finland	FIN	Western Europe	26	5	100
France	FRA	Western Europe	25	4	99.9
Germany	DEU	Western Europe	25	4	100
Greece	GRC	Western Europe	23	4	100
Iceland	ISL	Western Europe	26	5	100
Ireland	IRL	Western Europe	25	5	100
Israel	ISR	Western Europe	23	4	100
Italy	ITA	Western Europe	24	5	100
Luxembourg	LUX	Western Europe	25	4	--
Malta	MLT	Western Europe	25	5	99.9

© 2021 Weaver MR et al. *JAMA Network Open*.

Location	ISO code	Region	Number of years from 1990 to 2016 with complete death registration[1]	Data quality rating from 2010-2016[2]	Percentage of population with health insurance coverage in 2016[3]
Netherlands	NLD	Western Europe	25	4	100
Northern Ireland	NIR	Western Europe	24	5	100
Norway	NOR	Western Europe	25	4	100
Portugal	PRT	Western Europe	25	4	100
Scotland	SCT	Western Europe	24	5	100
Spain	ESP	Western Europe	25	5	99.9[6]
Sweden	SWE	Western Europe	25	4	100
Switzerland	CHE	Western Europe	25	5	100
Wales	WLS	Western Europe	24	5	100

Footnotes to Table S3:

1 GBD 2016 Mortality Collaborators. Global, regional, and national under-5 mortality, adult mortality, age-specific mortality, and life expectancy, 1970-2016: a systematic analysis for the Global Burden of Disease Study 2016. Lancet 2017; 390: 1084–150.

2 Ideally, mortality data by cause would be computed from a complete series of vital registration data with a high percentage of well-certified deaths, which for vital registration data means that the cause of death is well defined. Every country in the high income super-region had vital registration data from 2010 to 2016, with the exception of Andorra. For GBD 2016, the quality of data for each 5-year period from 1980 to 2009, and 2010 to 2016 has a star rating where 5 stars mean 85-100% are well-certified, 4 stars mean 65-85% well-certified, 3 stars mean 45-64% well certified, 2 stars mean 25-44% well certified, 1 star means 0-24% well certified, and 0 mean no data available.

GBD 2016 Causes of Death Collaborators. Global, regional, and national age-sex specific mortality for 264 causes of death, 1980-2016: a systematic analysis for the Global Burden of Disease Study 2016. Lancet 2017; 390: 1151–210.

3 Organization for Economic Cooperation and Development. Statistics; Social Protection: Total public and primary private health insurance. https://stats.oecd.org/# (accessed June 12, 2020).

4 Earn LC. International Health Care System Profiles: Singapore. New York: Commonwealth Fund, 2020. https://www.commonwealthfund.org/international-health-policy-center/countries/singapore (accessed June 12, 2020).

5 Pedersen ML, Rolskov A, Jacobsen JL, Lynge AR. Frequent use of primary health care service in Greenland: an opportunity for undiagnosed disease case-finding. Int J Circumpolar Health 2012; 71: 18431.

© 2021 Weaver MR et al. *JAMA Network Open*.

6 Organization for Economic Cooperation and Development statistic is for 2017.

© 2021 Weaver MR et al. *JAMA Network Open*.

eAppendix. Cause-Specific Mortality Scores by Age Category in the US in 2016

Examining the individual causes in the age-specific HAQ index, the US scores in 2016 are relatively high for some causes such as tetanus, tuberculosis, and Hodgkin lymphoma across all ages (Figure S1). The US performs poorly for many causes however, scoring below 70 for at least one cause in every age category, where the scores are rescaled to the observed global minimum and maximum within each age category, as described above. For example, for children zero to six days, the US performs poorly on neonatal disorders and adverse effects of medical treatment, scoring 61 and 57, respectively. For children 28 to 364 days, the lowest scores are 54 for cerebrovascular disease, and 59 for chronic kidney disease.

The number of causes used to construct the HAQ index differs across age categories. Cause-specific mortality scores are not included in the age-specific HAQ index when GBD results are not estimated or the cause is not amenable for an age category, as described above. For example, Diabetes mellitus is not considered amenable for age 50 or more years. The age-specific HAQ index is a weighted average of nine causes for zero to 27 days, 25 causes for 10-14 years, 27 causes for 15-44 years, and 21 causes for 70-74 years. With the cause weights normalized to sum to one, the weights are larger for age categories with fewer causes. A low cause-specific mortality score will reduce the HAQ score for ages 0-27 day by more than the same score for ages 15-44 years.

Three age patterns are evident across amenable causes. Some scores are lowest for young adults, such as cervical cancer, leukemia, and diabetes mellitus in ages 20 to 39 years. Other scores are low through most working ages, such as ischemic heart disease in ages 25 to 64 years. Finally, some scores decline with age, such as lower respiratory infections and rheumatic heart disease beginning at 10 to 14 years, and chronic kidney disease beginning at 15 to 19 years.

Scores for young adults in the US are below 80 for two or more of the three age categories from 15-29 years for nine causes: lower respiratory tract infections, maternal disorders, cervical cancer, leukemia, ischemic heart disease, hypertensive heart disease, diabetes mellitus, chronic kidney disease, and congenital heart anomalies.

The difference between the highest and lowest HAQ scores differ across age categories and causes. The extremes in the age-specific HAQ scores occur in amenable early childhood; the largest difference is 17.4 points for children 7 to 27 days, and the smallest is 7.5 points for children 1 to 4 years (Figure S2). Across amenable causes, the largest difference in scores is 53 points for ischemic heart disease among people 30 to 34 years, meaning that the risk-adjusted mortality rate for one state could be close to the global minimum, and for another state could be below the mid-point on the scale. Six causes have differences of 40 points or more for working age adults, such as leukemia for 35 to 44 years, and cerebrovascular disease for 30 to 34 and 50 to 54 years.

© 2021 Weaver MR et al. *JAMA Network Open*.

eFigure 1. Age-Specific HAQ Index and Cause-Specific Mortality Scores for the US in 2016

Heat map presents the national results for the age-specific HAQ index by age category for 2016 and national scores for each amenable cause. Cause-specific mortality scores are not included in the age-specific HAQ index when GBD results are not estimated or when the cause is not amenable for an age category. For example, GBD does not estimate the mortality rate for maternal condition for ages 0-9 years. Diabetes mellitus is not considered amenable for ages 50 or more years.

© 2021 Weaver MR et al. *JAMA Network Open*.

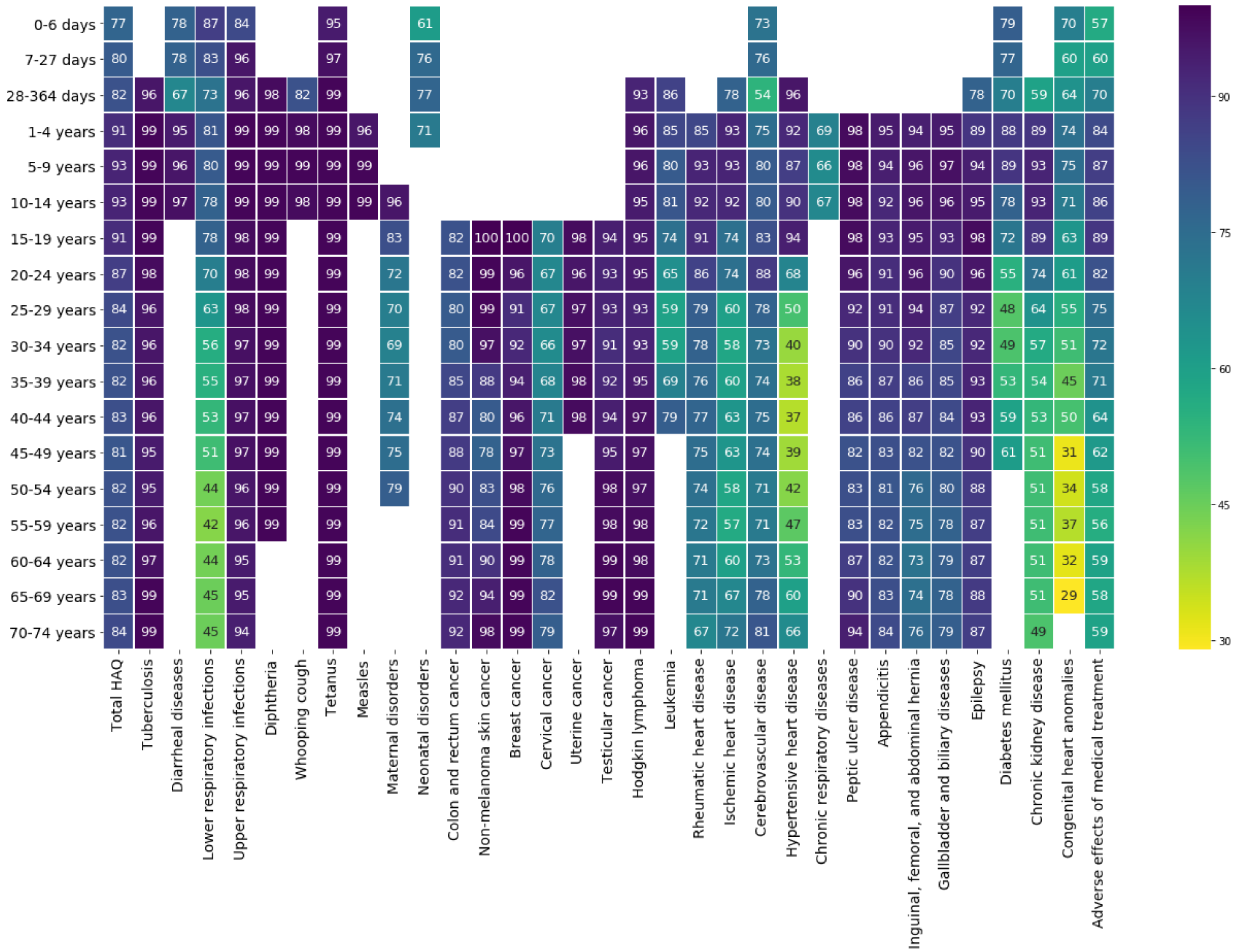

© 2021 Weaver MR et al. *JAMA Network Open*.

eFigure 2. Range in Age-Specific HAQ Index and Cause-Specific Mortality Scores Across US States in 2016

Heat map presents the difference between the minimum and maximum state-level results for the age-specific HAQ index by age category in 2016, and for each amenable cause. Cause-specific mortality scores are not included in the age-specific HAQ index when GBD results are not estimated or when the cause is not amenable for an age category. For example, GBD does not estimate the mortality rate for maternal condition for ages 0-9 years. Diabetes mellitus is not considered amenable for ages 50 or more years.

© 2021 Weaver MR et al. *JAMA Network Open*.

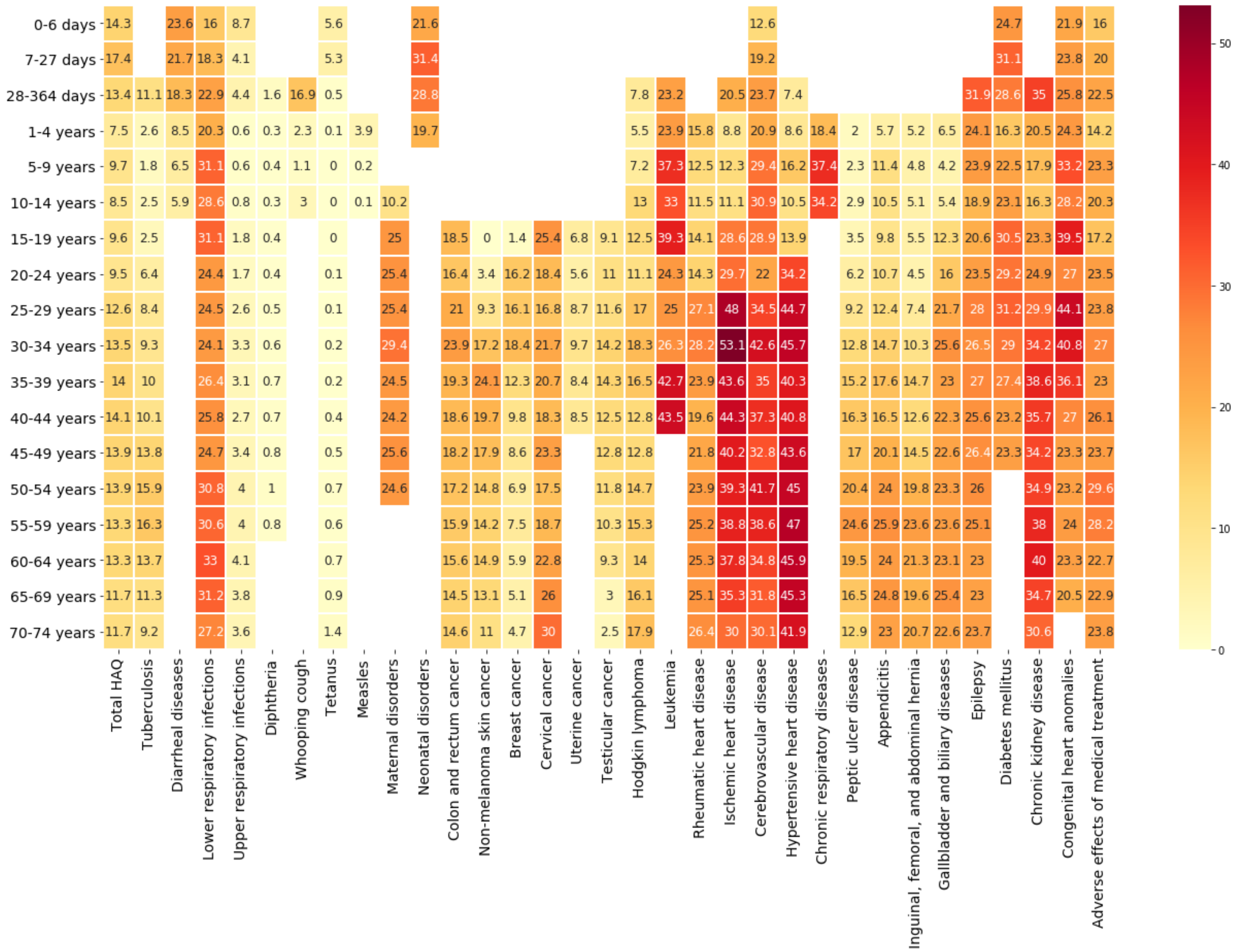

© 2021 Weaver MR et al. *JAMA Network Open*.

eTable 4. Comparison of Estimated Coefficients Multiplied by SD for Age-Specific Total Insurance Coverage and Median Income Per Capita by Age Category in the United States, 2016

Age category (1)	Age-specific total insurance coverage			Age-specific median income per capita in thousands of US dollars			
	Coeffi-cient (2)	Standard Deviation (3)	Coefficient * Standard Deviation (4)	Coeffi-cient (5)	Standard Deviation (6)	Coefficient * Standard Deviation (7)	Ratio of column 4 to column 7 (8)
**(a) Model with age fixed effects**							
0-11 months	-0.049	2.092	-0.103	0.535	3.719	1.991	-0.052
1-4 years	0.094	2.959	0.280	0.425	3.422	1.453	0.192
5-9 years	-0.015	3.083	-0.045	0.421	2.889	1.215	-0.037
10-14 years	-0.132	3.382	-0.445	0.453	3.221	1.460	-0.305
15-19 years	0.188	5.550	1.045	0.317	3.066	0.972	1.075
20-24 years	0.143	11.024	1.575	0.233	3.288	0.767	2.054
25-29 years	0.104	9.183	0.960	0.352	4.868	1.715	0.559
30-34 years	0.197	7.916	1.561	0.225	6.196	1.394	1.120
35-39 years	0.250	6.623	1.659	0.155	5.953	0.920	1.803
40-44 years	0.264	6.262	1.654	0.236	4.536	1.068	1.548
45-49 years	0.295	6.012	1.776	0.219	4.771	1.044	1.702
50-54 years	0.251	5.236	1.312	0.369	4.583	1.693	0.775
55-59 years	0.125	4.343	0.542	0.479	4.788	2.291	0.237
60-64 years	0.226	3.726	0.843	0.401	4.750	1.903	0.443
65-69 years	0.372	0.867	0.322	0.451	4.565	2.057	0.157
70-74 years	-0.401	0.627	-0.251	0.273	4.502	1.228	-0.204
**(b) Models with age and state fixed effects**							
0-11 months	-0.008	2.092	-0.017	0.089	3.719	0.332	-0.052
1-4 years	0.080	2.959	0.235	-0.085	3.422	-0.289	-0.814
5-9 years	0.021	3.083	0.064	-0.235	2.889	-0.679	-0.094
10-14 years	-0.069	3.382	-0.233	-0.160	3.221	-0.516	0.451
15-19 years	0.034	5.550	0.188	-0.154	3.066	-0.471	-0.400
20-24 years	0.059	11.024	0.648	-0.241	3.288	-0.792	-0.818
25-29 years	0.005	9.183	0.046	0.160	4.868	0.779	0.059
30-34 years	0.004	7.916	0.028	0.214	6.196	1.324	0.021
35-39 years	0.026	6.623	0.169	0.167	5.953	0.991	0.171
40-44 years	0.057	6.262	0.360	0.184	4.536	0.835	0.431
45-49 years	0.088	6.012	0.528	0.112	4.771	0.536	0.986
50-54 years	0.101	5.236	0.529	0.117	4.583	0.537	0.985
55-59 years	0.039	4.343	0.168	0.154	4.788	0.736	0.228
60-64 years	0.113	3.726	0.422	0.095	4.750	0.449	0.940
65-69 years	0.123	0.867	0.107	0.071	4.565	0.322	0.331
70-74 years	-0.635	0.627	-0.398	0.020	4.502	0.089	-4.451

© 2021 Weaver MR et al. *JAMA Network Open*.

The coefficients are also reported in Table 1 of the main text with the 99% confidence intervals and p-values. All regression models have age fixed effects, and age interaction for insurance coverage, median household income per capita, and year.

© 2021 Weaver MR et al. *JAMA Network Open*.

eTable 5. Association Between HAQ Scores and Total Health Insurance Coverage by Age Category in the United States, 2010 and 2016; Sensitivity Analysis With State-Level Variable for Hospital Beds Per 1000 Population

Age category	Age-specific total insurance coverage		Age-specific median income per capita in thousands of US dollars		Year = 2016	
	Coefficient (99% CI)	p-value	Coefficient (99% CI)	p-value	Coefficient (99% CI)	p-value
**(a) Model with age fixed effects**						
0-11 months	0.0 (-0.266 to 0.267)	0.997	0.513 (0.352 to 0.674)	<0.001	-0.564 (-1.698 to 0.57)	0.202
1-4 years	0.145 (-0.049 to 0.339)	0.056	0.384 (0.21 to 0.559)	<0.001	-0.997 (-2.183 to 0.19)	0.031
5-9 years	0.064 (-0.126 to 0.254)	0.390	0.266 (0.067 to 0.465)	0.001	0.123 (-1.014 to 1.26)	0.781
10-14 years	-0.008 (-0.197 to 0.18)	0.912	0.306 (0.119 to 0.493)	<0.001	-0.124 (-1.318 to 1.071)	0.791
15-19 years	0.241 (0.12 to 0.361)	<0.001	0.109 (-0.08 to 0.298)	0.139	-0.659 (-1.989 to 0.671)	0.204
20-24 years	0.191 (0.11 to 0.272)	<0.001	0.022 (-0.186 to 0.23)	0.783	-3.068 (-4.696 to -1.441)	<0.001
25-29 years	0.127 (0.024 to 0.23)	0.002	0.344 (0.189 to 0.5)	<0.001	-3.286 (-4.714 to -1.859)	<0.001
30-34 years	0.184 (0.08 to 0.288)	<0.001	0.27 (0.16 to 0.381)	<0.001	-3.655 (-4.956 to -2.354)	<0.001
35-39 years	0.237 (0.13 to 0.344)	<0.001	0.21 (0.105 to 0.315)	<0.001	-2.84 (-4.053 to -1.627)	<0.001
40-44 years	0.266 (0.149 to 0.383)	<0.001	0.262 (0.114 to 0.411)	<0.001	-2.352 (-3.54 to -1.163)	<0.001
45-49 years	0.297 (0.173 to 0.42)	<0.001	0.236 (0.09 to 0.382)	<0.001	-2.968 (-4.223 to -1.713)	<0.001
50-54 years	0.277 (0.14 to 0.415)	<0.001	0.339 (0.197 to 0.481)	<0.001	-3.388 (-4.62 to -2.157)	<0.001
55-59 years	0.189 (0.03 to 0.348)	0.002	0.409 (0.281 to 0.538)	<0.001	-2.371 (-3.57 to -1.172)	<0.001
60-64 years	0.332 (0.152 to 0.512)	<0.001	0.324 (0.199 to 0.449)	<0.001	-2.632 (-3.845 to -1.419)	<0.001
65-69 years	0.692 (0.072 to 1.313)	0.004	0.408 (0.274 to 0.541)	<0.001	-1.899 (-3.149 to -0.648)	<0.001
70-74 years	-0.138 (-0.993 to 0.716)	0.678	0.282 (0.138 to 0.426)	<0.001	-1.144 (-2.448 to 0.16)	0.025
**(b) Model with age and state fixed-effects**						
0-11 months	0.014 (-0.15 to 0.178)	0.825	0.087 (-0.019 to 0.193)	0.036	0.908 (0.182 to 1.635)	0.001
1-4 years	0.101 (-0.02 to 0.222)	0.032	-0.09 (-0.206 to 0.025)	0.045	0.715 (-0.046 to 1.476)	0.016
5-9 years	0.043 (-0.076 to 0.162)	0.352	-0.24 (-0.37 to -0.109)	0.000	1.288 (0.568 to 2.008)	0.000
10-14 years	-0.051 (-0.169 to 0.067)	0.264	-0.163 (-0.285 to -0.041)	0.001	1.314 (0.555 to 2.074)	0.000
15-19 years	0.044 (-0.034 to 0.122)	0.149	-0.154 (-0.273 to -0.036)	0.001	1.468 (0.616 to 2.319)	0.000
20-24 years	0.066 (0.014 to 0.118)	0.001	-0.245 (-0.376 to -0.113)	<0.001	0.166 (-0.89 to 1.222)	0.687

© 2021 Weaver MR et al. *JAMA Network Open*.

25-29 years	0.014 (-0.05 to 0.079)	0.567	0.153 (0.054 to 0.252)	<0.001	-0.953 (-1.859 to -0.048)	0.007
30-34 years	0.011 (-0.055 to 0.077)	0.675	0.21 (0.14 to 0.28)	<0.001	-1.635 (-2.464 to -0.805)	<0.001
35-39 years	0.034 (-0.035 to 0.103)	0.203	0.163 (0.097 to 0.229)	<0.001	-1.111 (-1.884 to -0.337)	<0.001
40-44 years	0.067 (-0.008 to 0.141)	0.021	0.179 (0.086 to 0.273)	<0.001	-0.688 (-1.447 to 0.072)	0.020
45-49 years	0.098 (0.02 to 0.177)	0.001	0.103 (0.01 to 0.195)	0.004	-0.896 (-1.708 to -0.084)	0.005
50-54 years	0.112 (0.024 to 0.199)	0.001	0.111 (0.019 to 0.202)	0.002	-1.477 (-2.268 to -0.687)	0.000
55-59 years	0.05 (-0.051 to 0.151)	0.203	0.152 (0.07 to 0.235)	<0.001	-0.897 (-1.66 to -0.134)	0.003
60-64 years	0.127 (0.013 to 0.242)	0.004	0.092 (0.011 to 0.172)	0.004	-1.009 (-1.781 to -0.236)	0.001
65-69 years	0.152 (-0.232 to 0.537)	0.309	0.072 (-0.018 to 0.161)	0.040	0.034 (-0.771 to 0.838)	0.914
70-74 years	-0.632 (-1.156 to -0.109)	0.002	0.017 (-0.076 to 0.11)	0.642	0.469 (-0.368 to 1.305)	0.150
All regression models have age fixed effects, and age interaction for insurance coverage, median household income per capita, and year. To address multiple testing, results presented with 99% confidence intervals (CI). For model (a), the coefficient for hospital beds per 1,000 population is -1.229 (99% CI -1.365, -1.094), p-value = <0.001 for all age categories. The mean square error (MSE) is 4.36 and square root of MSE (RMSE) is 2.09. For model (b), the coefficient for hospital beds per 1,000 population is -1.900 (99% CI -3.134, 0.666), p-value <0.001 for all age categories. The MSE is 1.61 and RMSE is 1.27.						

© 2021 Weaver MR et al. *JAMA Network Open*.

eTable 6. Association between HAQ scores and total health insurance coverage by age category in the United States, 2016; sensitivity analysis with state-level variable for physicians per 1000 population

Age category	Age-specific total insurance coverage		Age-specific median income per capita in thousands of US dollars		Year = 2016	
	Coefficient (99% CI)	p-value	Coefficient (99% CI)	p-value	Coefficient (99% CI)	p-value
**(a) Model with age fixed effects**						
0-11 months	-0.046 (-0.355 to 0.263)	0.702	0.541 (0.351 to 0.73)	<0.001	-0.83 (-2.151 to 0.492)	0.107
1-4 years	0.097 (-0.129 to 0.322)	0.272	0.431 (0.225 to 0.636)	<0.001	-1.255 (-2.639 to 0.129)	0.020
5-9 years	-0.013 (-0.233 to 0.208)	0.882	0.426 (0.194 to 0.658)	<0.001	-0.229 (-1.55 to 1.093)	0.657
10-14 years	-0.129 (-0.348 to 0.089)	0.129	0.457 (0.24 to 0.675)	<0.001	-0.32 (-1.71 to 1.07)	0.555
15-19 years	0.192 (0.051 to 0.333)	<0.001	0.316 (0.098 to 0.533)	<0.001	-1.066 (-2.614 to 0.481)	0.077
20-24 years	0.145 (0.05 to 0.24)	<0.001	0.235 (-0.005 to 0.475)	0.012	-3.292 (-5.198 to -1.385)	<0.001
25-29 years	0.104 (-0.015 to 0.224)	0.026	0.359 (0.175 to 0.543)	<0.001	-3.279 (-4.941 to -1.617)	<0.001
30-34 years	0.197 (0.076 to 0.317)	<0.001	0.23 (0.098 to 0.363)	<0.001	-3.849 (-5.362 to -2.336)	<0.001
35-39 years	0.251 (0.126 to 0.375)	<0.001	0.16 (0.035 to 0.285)	0.001	-2.988 (-4.4 to -1.576)	<0.001
40-44 years	0.264 (0.129 to 0.399)	<0.001	0.243 (0.066 to 0.419)	<0.001	-2.497 (-3.883 to -1.112)	<0.001
45-49 years	0.296 (0.153 to 0.439)	<0.001	0.224 (0.052 to 0.396)	0.001	-3.133 (-4.6 to -1.667)	<0.001
50-54 years	0.252 (0.092 to 0.411)	<0.001	0.373 (0.207 to 0.54)	<0.001	-3.587 (-5.021 to -2.153)	<0.001
55-59 years	0.127 (-0.058 to 0.312)	0.077	0.481 (0.332 to 0.63)	<0.001	-2.512 (-3.907 to -1.116)	<0.001
60-64 years	0.229 (0.02 to 0.437)	0.005	0.404 (0.258 to 0.549)	<0.001	-2.675 (-4.087 to -1.263)	<0.001
65-69 years	0.368 (-0.352 to 1.087)	0.190	0.456 (0.298 to 0.614)	<0.001	-2.211 (-3.669 to -0.754)	<0.001
70-74 years	-0.418 (-1.413 to 0.578)	0.282	0.279 (0.108 to 0.45)	<0.001	-1.289 (-2.812 to 0.235)	0.030
**(b) Model with age and state fixed-effects**						
0-11 months	-0.007 (-0.171 to 0.158)	0.918	0.088 (-0.018 to 0.195)	0.033	0.556 (-0.144 to 1.255)	0.042
1-4 years	0.083 (-0.038 to 0.204)	0.079	-0.086 (-0.202 to 0.03)	0.058	0.375 (-0.364 to 1.115)	0.193
5-9 years	0.024 (-0.095 to 0.142)	0.608	-0.236 (-0.368 to -0.105)	<0.001	0.96 (0.26 to 1.66)	<0.001
10-14 years	-0.065 (-0.184 to 0.053)	0.155	-0.162 (-0.285 to -0.04)	0.001	0.993 (0.251 to 1.735)	0.001
15-19 years	0.035 (-0.043 to 0.114)	0.246	-0.154 (-0.273 to -0.035)	0.001	1.159 (0.319 to 1.998)	<0.001
20-24 years	0.06 (0.008 to 0.112)	0.003	-0.243 (-0.374 to -0.111)	<0.001	-0.108 (-1.161 to 0.944)	0.792

© 2021 Weaver MR et al. *JAMA Network Open*.

25-29 years	0.006 (-0.058 to 0.071)	0.802	0.158 (0.059 to 0.258)	<0.001	-1.241 (-2.139 to -0.343)	<0.001
30-34 years	0.005 (-0.062 to 0.071)	0.856	0.213 (0.142 to 0.283)	<0.001	-1.953 (-2.768 to -1.138)	<0.001
35-39 years	0.027 (-0.042 to 0.096)	0.316	0.165 (0.099 to 0.232)	<0.001	-1.435 (-2.192 to -0.679)	<0.001
40-44 years	0.059 (-0.015 to 0.134)	0.042	0.182 (0.088 to 0.277)	<0.001	-1.017 (-1.758 to -0.275)	<0.001
45-49 years	0.089 (0.01 to 0.168)	0.004	0.111 (0.018 to 0.203)	0.002	-1.239 (-2.031 to -0.446)	<0.001
50-54 years	0.102 (0.014 to 0.19)	0.003	0.116 (0.024 to 0.208)	0.001	-1.809 (-2.58 to -1.038)	<0.001
55-59 years	0.04 (-0.061 to 0.142)	0.305	0.153 (0.07 to 0.236)	<0.001	-1.224 (-1.969 to -0.479)	<0.001
60-64 years	0.115 (0.001 to 0.23)	0.010	0.094 (0.013 to 0.175)	0.003	-1.335 (-2.09 to -0.579)	<0.001
65-69 years	0.124 (-0.262 to 0.51)	0.411	0.07 (-0.02 to 0.16)	0.045	-0.315 (-1.096 to 0.466)	0.301
70-74 years	-0.638 (-1.164 to -0.112)	0.002	0.019 (-0.075 to 0.113)	0.606	0.092 (-0.717 to 0.901)	0.770

All regression models have age fixed effects, and age interaction for insurance coverage, median household income per capita, and year. To address multiple testing, results presented with 99% confidence intervals (CI). For model (a), the coefficient for physicians per 1,000 population is -0.004 (99% CI -0.029, 0.020), p-value = 0.651 for all age categories. The mean square error (MSE) is 6.07 and square root of MSE (RMSE) is 2.46. For model (b), the coefficient for physicians per 1,000 population is -0.074 (99% CI -0.226, 0.078), p-value 0.213 for all age categories. The MSE is 1.63 and RMSE is 1.28.

© 2021 Weaver MR et al. *JAMA Network Open*.

eTable 7. Detailed Counterfactual Estimates of the Increase in HAQ Score With Universal Health Insurance Coverage by State and Age Category, 2010 and 2016

		Increase in HAQ score (99% confidence interval)			
State	Age category	2010		2016	
		Model with age fixed effects	Model with age and state fixed effects	Model with age fixed effects	Model with age and state fixed effects
Alabama	0 to 11 months	-0.2 (-1.4 to 1.0)	0.0 (-0.7 to 0.6)	-0.1 (-0.6 to 0.5)	0.0 (-0.3 to 0.3)
Alabama	1 to 4 years	0.4 (-0.6 to 1.4)	0.3 (-0.2 to 0.9)	0.2 (-0.3 to 0.8)	0.2 (-0.1 to 0.5)
Alabama	10 to 14 years	-0.8 (-2.0 to 0.5)	-0.4 (-1.1 to 0.3)	-0.2 (-0.6 to 0.2)	-0.1 (-0.3 to 0.1)
Alabama	15 to 19 years	2.6 (0.7 to 4.5)	0.5 (-0.6 to 1.5)	1.3 (0.3 to 2.3)	0.2 (-0.3 to 0.8)
Alabama	20 to 24 years	4.7 (1.6 to 7.9)	1.9 (0.2 to 3.7)	2.4 (0.8 to 4.0)	1.0 (0.1 to 1.9)
Alabama	25 to 29 years	3.6 (-0.5 to 7.8)	0.2 (-2.1 to 2.4)	2.4 (-0.4 to 5.2)	0.1 (-1.4 to 1.6)
Alabama	30 to 34 years	5.2 (2.0 to 8.3)	0.1 (-1.6 to 1.8)	4.0 (1.5 to 6.4)	0.1 (-1.3 to 1.4)
Alabama	35 to 39 years	5.9 (3.0 to 8.9)	0.6 (-1.0 to 2.2)	4.4 (2.2 to 6.6)	0.4 (-0.8 to 1.7)
Alabama	40 to 44 years	5.3 (2.6 to 8.0)	1.2 (-0.4 to 2.7)	4.1 (2.0 to 6.2)	0.9 (-0.3 to 2.1)
Alabama	45 to 49 years	5.7 (2.9 to 8.5)	1.7 (0.2 to 3.2)	3.8 (1.9 to 5.6)	1.1 (0.1 to 2.1)
Alabama	5 to 9 years	-0.1 (-1.6 to 1.4)	0.1 (-0.7 to 0.9)	0.0 (-0.5 to 0.4)	0.0 (-0.2 to 0.3)
Alabama	50 to 54 years	4.2 (1.5 to 6.9)	1.7 (0.2 to 3.2)	3.1 (1.1 to 5.0)	1.2 (0.2 to 2.3)
Alabama	55 to 59 years	1.4 (-0.7 to 3.4)	0.4 (-0.7 to 1.6)	1.2 (-0.6 to 2.9)	0.4 (-0.6 to 1.3)
Alabama	60 to 64 years	2.4 (0.2 to 4.7)	1.2 (0.0 to 2.5)	1.7 (0.1 to 3.4)	0.9 (0.0 to 1.8)
Alabama	65 to 69 years	0.2 (-0.2 to 0.5)	0.1 (-0.1 to 0.2)	0.1 (-0.1 to 0.4)	0.0 (-0.1 to 0.2)
Alabama	70 to 74 years	-0.1 (-0.3 to 0.1)	-0.1 (-0.2 to 0.0)	-0.4 (-1.3 to 0.5)	-0.6 (-1.1 to -0.1)
Alaska	0 to 11 months	-0.1 (-0.5 to 0.4)	0.0 (-0.3 to 0.2)	-0.4 (-2.9 to 2.1)	-0.1 (-1.4 to 1.2)
Alaska	1 to 4 years	0.7 (-0.9 to 2.3)	0.6 (-0.3 to 1.4)	1.0 (-1.4 to 3.4)	0.8 (-0.4 to 2.1)
Alaska	10 to 14 years	-1.3 (-3.5 to 0.9)	-0.7 (-1.9 to 0.5)	-1.4 (-3.8 to 1.0)	-0.7 (-2.0 to 0.5)
Alaska	15 to 19 years	3.7 (1.0 to 6.5)	0.7 (-0.9 to 2.2)	2.4 (0.6 to 4.2)	0.4 (-0.6 to 1.5)
Alaska	20 to 24 years	4.1 (1.4 to 6.8)	1.7 (0.2 to 3.2)	3.0 (1.0 to 4.9)	1.2 (0.1 to 2.3)
Alaska	25 to 29 years	3.2 (-0.5 to 7.0)	0.2 (-1.9 to 2.2)	2.7 (-0.4 to 5.8)	0.1 (-1.6 to 1.8)
Alaska	30 to 34 years	4.8 (1.9 to 7.8)	0.1 (-1.5 to 1.7)	3.7 (1.4 to 6.0)	0.1 (-1.2 to 1.3)

© 2021 Weaver MR et al. *JAMA Network Open*.

Alaska	35 to 39 years	5.6 (2.8 to 8.4)	0.6 (-1.0 to 2.1)	4.0 (2.0 to 6.0)	0.4 (-0.7 to 1.5)
Alaska	40 to 44 years	4.8 (2.3 to 7.3)	1.0 (-0.3 to 2.4)	4.6 (2.2 to 7.0)	1.0 (-0.3 to 2.3)
Alaska	45 to 49 years	5.7 (2.9 to 8.5)	1.7 (0.2 to 3.2)	4.5 (2.3 to 6.6)	1.3 (0.1 to 2.5)
Alaska	5 to 9 years	-0.1 (-2.3 to 2.0)	0.2 (-0.9 to 1.3)	-0.1 (-2.4 to 2.1)	0.2 (-1.0 to 1.4)
Alaska	50 to 54 years	5.4 (1.9 to 8.8)	2.2 (0.3 to 4.0)	4.5 (1.6 to 7.4)	1.8 (0.2 to 3.4)
Alaska	55 to 59 years	2.3 (-1.1 to 5.6)	0.7 (-1.1 to 2.5)	2.0 (-1.0 to 4.9)	0.6 (-1.0 to 2.2)
Alaska	60 to 64 years	4.5 (0.3 to 8.7)	2.3 (0.0 to 4.6)	2.3 (0.2 to 4.4)	1.1 (0.0 to 2.3)
Alaska	65 to 69 years	2.0 (-1.9 to 5.9)	0.7 (-1.4 to 2.8)	0.1 (-0.1 to 0.3)	0.0 (-0.1 to 0.2)
Alaska	70 to 74 years	-0.5 (-1.7 to 0.7)	-0.8 (-1.4 to -0.1)	-0.1 (-0.5 to 0.2)	-0.2 (-0.4 to 0.0)
Arizona	0 to 11 months	-0.3 (-2.6 to 1.9)	-0.1 (-1.2 to 1.1)	-0.2 (-1.3 to 1.0)	0.0 (-0.6 to 0.6)
Arizona	1 to 4 years	1.1 (-1.5 to 3.6)	0.9 (-0.5 to 2.2)	0.6 (-0.9 to 2.2)	0.5 (-0.3 to 1.4)
Arizona	10 to 14 years	-1.9 (-5.1 to 1.3)	-1.0 (-2.7 to 0.7)	-1.0 (-2.6 to 0.6)	-0.5 (-1.4 to 0.4)
Arizona	15 to 19 years	3.9 (1.0 to 6.8)	0.7 (-0.9 to 2.3)	2.3 (0.6 to 4.0)	0.4 (-0.5 to 1.4)
Arizona	20 to 24 years	5.0 (1.7 to 8.4)	2.1 (0.2 to 3.9)	2.4 (0.8 to 4.0)	1.0 (0.1 to 1.9)
Arizona	25 to 29 years	3.0 (-0.5 to 6.5)	0.1 (-1.7 to 2.0)	2.0 (-0.3 to 4.2)	0.1 (-1.1 to 1.3)
Arizona	30 to 34 years	5.3 (2.0 to 8.5)	0.1 (-1.7 to 1.9)	3.4 (1.3 to 5.6)	0.1 (-1.1 to 1.2)
Arizona	35 to 39 years	6.2 (3.1 to 9.3)	0.6 (-1.1 to 2.4)	4.1 (2.1 to 6.2)	0.4 (-0.7 to 1.6)
Arizona	40 to 44 years	6.1 (3.0 to 9.3)	1.3 (-0.4 to 3.1)	4.0 (1.9 to 6.0)	0.9 (-0.3 to 2.0)
Arizona	45 to 49 years	6.0 (3.1 to 8.9)	1.8 (0.2 to 3.4)	4.1 (2.1 to 6.1)	1.2 (0.1 to 2.3)
Arizona	5 to 9 years	-0.2 (-2.7 to 2.4)	0.2 (-1.1 to 1.6)	-0.1 (-1.7 to 1.5)	0.1 (-0.7 to 1.0)
Arizona	50 to 54 years	4.5 (1.6 to 7.4)	1.8 (0.2 to 3.4)	3.2 (1.1 to 5.2)	1.3 (0.2 to 2.4)
Arizona	55 to 59 years	1.9 (-0.9 to 4.6)	0.6 (-0.9 to 2.1)	1.2 (-0.6 to 3.0)	0.4 (-0.6 to 1.4)
Arizona	60 to 64 years	2.8 (0.2 to 5.4)	1.4 (0.0 to 2.8)	1.9 (0.1 to 3.6)	0.9 (0.0 to 1.9)
Arizona	65 to 69 years	0.6 (-0.6 to 1.8)	0.2 (-0.4 to 0.8)	0.5 (-0.4 to 1.4)	0.2 (-0.3 to 0.6)
Arizona	70 to 74 years	-0.5 (-1.9 to 0.8)	-0.8 (-1.5 to -0.1)	-0.2 (-0.5 to 0.2)	-0.2 (-0.5 to 0.0)
Arkansas	0 to 11 months	-0.1 (-0.4 to 0.3)	0.0 (-0.2 to 0.2)	-0.3 (-2.1 to 1.5)	0.0 (-1.0 to 0.9)
Arkansas	1 to 4 years	0.5 (-0.7 to 1.6)	0.4 (-0.2 to 1.0)	0.4 (-0.5 to 1.3)	0.3 (-0.2 to 0.8)
Arkansas	10 to 14 years	-1.0 (-2.8 to 0.7)	-0.5 (-1.5 to 0.4)	-0.5 (-1.4 to 0.4)	-0.3 (-0.8 to 0.2)
Arkansas	15 to 19 years	3.4 (0.9 to 6.0)	0.6 (-0.8 to 2.1)	1.4 (0.4 to 2.4)	0.3 (-0.3 to 0.8)

© 2021 Weaver MR et al. *JAMA Network Open*.

Arkansas	20 to 24 years	5.8 (2.0 to 9.7)	2.4 (0.3 to 4.5)	2.5 (0.9 to 4.2)	1.0 (0.1 to 2.0)
Arkansas	25 to 29 years	3.7 (-0.5 to 7.9)	0.2 (-2.1 to 2.4)	1.7 (-0.3 to 3.7)	0.1 (-1.0 to 1.1)
Arkansas	30 to 34 years	6.1 (2.3 to 9.8)	0.1 (-1.9 to 2.2)	3.4 (1.3 to 5.5)	0.1 (-1.1 to 1.2)
Arkansas	35 to 39 years	7.0 (3.5 to 10.5)	0.7 (-1.2 to 2.7)	3.8 (1.9 to 5.7)	0.4 (-0.7 to 1.4)
Arkansas	40 to 44 years	7.4 (3.6 to 11.2)	1.6 (-0.5 to 3.7)	3.9 (1.9 to 6.0)	0.9 (-0.3 to 2.0)
Arkansas	45 to 49 years	7.0 (3.6 to 10.4)	2.1 (0.2 to 4.0)	3.3 (1.7 to 4.9)	1.0 (0.1 to 1.9)
Arkansas	5 to 9 years	-0.1 (-1.1 to 0.9)	0.1 (-0.4 to 0.6)	0.0 (-0.7 to 0.6)	0.1 (-0.3 to 0.4)
Arkansas	50 to 54 years	4.6 (1.7 to 7.5)	1.9 (0.2 to 3.5)	2.3 (0.8 to 3.8)	0.9 (0.1 to 1.7)
Arkansas	55 to 59 years	2.1 (-1.0 to 5.3)	0.7 (-1.1 to 2.4)	0.9 (-0.4 to 2.2)	0.3 (-0.4 to 1.0)
Arkansas	60 to 64 years	3.2 (0.2 to 6.2)	1.6 (0.0 to 3.2)	1.0 (0.1 to 2.0)	0.5 (0.0 to 1.1)
Arkansas	65 to 69 years	0.1 (-0.1 to 0.3)	0.0 (-0.1 to 0.2)	0.3 (-0.3 to 0.9)	0.1 (-0.2 to 0.4)
Arkansas	70 to 74 years	0.0 (-0.1 to 0.0)	0.0 (-0.1 to 0.0)	-0.3 (-0.9 to 0.4)	-0.4 (-0.8 to -0.1)
California	0 to 11 months	-0.2 (-1.5 to 1.1)	0.0 (-0.7 to 0.7)	-0.1 (-0.8 to 0.6)	0.0 (-0.4 to 0.3)
California	1 to 4 years	0.6 (-0.9 to 2.1)	0.5 (-0.3 to 1.3)	0.2 (-0.3 to 0.8)	0.2 (-0.1 to 0.5)
California	10 to 14 years	-1.4 (-3.6 to 0.9)	-0.7 (-1.9 to 0.5)	-0.4 (-1.2 to 0.3)	-0.2 (-0.6 to 0.2)
California	15 to 19 years	3.4 (0.9 to 5.9)	0.6 (-0.8 to 2.0)	1.1 (0.3 to 1.9)	0.2 (-0.3 to 0.7)
California	20 to 24 years	5.3 (1.8 to 8.8)	2.2 (0.2 to 4.1)	1.6 (0.6 to 2.7)	0.7 (0.1 to 1.3)
California	25 to 29 years	3.5 (-0.5 to 7.5)	0.2 (-2.0 to 2.3)	1.3 (-0.2 to 2.8)	0.1 (-0.8 to 0.9)
California	30 to 34 years	5.5 (2.1 to 8.9)	0.1 (-1.8 to 2.0)	2.6 (1.0 to 4.1)	0.0 (-0.8 to 0.9)
California	35 to 39 years	6.6 (3.3 to 9.9)	0.7 (-1.2 to 2.5)	3.3 (1.6 to 4.9)	0.3 (-0.6 to 1.2)
California	40 to 44 years	6.4 (3.1 to 9.6)	1.4 (-0.4 to 3.2)	3.1 (1.5 to 4.8)	0.7 (-0.2 to 1.6)
California	45 to 49 years	6.5 (3.3 to 9.7)	1.9 (0.2 to 3.7)	3.0 (1.6 to 4.5)	0.9 (0.1 to 1.7)
California	5 to 9 years	-0.1 (-1.9 to 1.7)	0.2 (-0.8 to 1.1)	0.0 (-0.6 to 0.5)	0.0 (-0.2 to 0.3)
California	50 to 54 years	5.1 (1.8 to 8.4)	2.1 (0.3 to 3.8)	2.3 (0.8 to 3.8)	0.9 (0.1 to 1.8)
California	55 to 59 years	2.2 (-1.1 to 5.6)	0.7 (-1.1 to 2.5)	0.9 (-0.4 to 2.2)	0.3 (-0.5 to 1.0)
California	60 to 64 years	3.5 (0.3 to 6.8)	1.8 (0.0 to 3.6)	1.4 (0.1 to 2.7)	0.7 (0.0 to 1.4)
California	65 to 69 years	1.1 (-1.0 to 3.1)	0.3 (-0.8 to 1.5)	0.6 (-0.6 to 1.7)	0.2 (-0.4 to 0.8)
California	70 to 74 years	-0.6 (-2.1 to 0.9)	-1.0 (-1.8 to -0.2)	-0.4 (-1.5 to 0.7)	-0.7 (-1.3 to -0.1)
Colorado	0 to 11 months	-0.2 (-1.6 to 1.2)	0.0 (-0.8 to 0.7)	-0.1 (-0.9 to 0.6)	0.0 (-0.4 to 0.4)

© 2021 Weaver MR et al. *JAMA Network Open*.

Colorado	1 to 4 years	0.8 (-1.1 to 2.6)	0.7 (-0.3 to 1.7)	0.3 (-0.5 to 1.1)	0.3 (-0.1 to 0.7)
Colorado	10 to 14 years	-1.4 (-3.8 to 0.9)	-0.7 (-2.0 to 0.5)	-0.6 (-1.6 to 0.4)	-0.3 (-0.8 to 0.2)
Colorado	15 to 19 years	3.0 (0.8 to 5.2)	0.5 (-0.7 to 1.8)	1.2 (0.3 to 2.1)	0.2 (-0.3 to 0.7)
Colorado	20 to 24 years	4.6 (1.6 to 7.7)	1.9 (0.2 to 3.6)	1.6 (0.6 to 2.7)	0.7 (0.1 to 1.3)
Colorado	25 to 29 years	3.2 (-0.5 to 6.8)	0.2 (-1.8 to 2.1)	1.5 (-0.2 to 3.2)	0.1 (-0.8 to 1.0)
Colorado	30 to 34 years	5.0 (1.9 to 8.2)	0.1 (-1.6 to 1.8)	2.6 (1.0 to 4.2)	0.0 (-0.8 to 0.9)
Colorado	35 to 39 years	5.1 (2.6 to 7.7)	0.5 (-0.9 to 1.9)	3.3 (1.6 to 4.9)	0.3 (-0.6 to 1.2)
Colorado	40 to 44 years	4.4 (2.1 to 6.6)	1.0 (-0.3 to 2.2)	3.7 (1.8 to 5.5)	0.8 (-0.2 to 1.8)
Colorado	45 to 49 years	5.4 (2.8 to 8.1)	1.6 (0.2 to 3.1)	2.9 (1.5 to 4.3)	0.9 (0.1 to 1.6)
Colorado	5 to 9 years	-0.1 (-2.3 to 2.0)	0.2 (-1.0 to 1.3)	-0.1 (-0.9 to 0.8)	0.1 (-0.4 to 0.6)
Colorado	50 to 54 years	3.8 (1.4 to 6.3)	1.5 (0.2 to 2.9)	2.0 (0.7 to 3.3)	0.8 (0.1 to 1.5)
Colorado	55 to 59 years	1.6 (-0.8 to 4.0)	0.5 (-0.8 to 1.8)	0.9 (-0.4 to 2.2)	0.3 (-0.4 to 1.0)
Colorado	60 to 64 years	2.5 (0.2 to 4.9)	1.3 (0.0 to 2.5)	1.5 (0.1 to 2.9)	0.8 (0.0 to 1.5)
Colorado	65 to 69 years	0.2 (-0.2 to 0.7)	0.1 (-0.2 to 0.3)	0.3 (-0.3 to 1.0)	0.1 (-0.2 to 0.4)
Colorado	70 to 74 years	-0.4 (-1.4 to 0.6)	-0.6 (-1.2 to -0.1)	-0.5 (-1.6 to 0.7)	-0.7 (-1.4 to -0.1)
Connecticut	0 to 11 months	-0.1 (-0.5 to 0.4)	0.0 (-0.3 to 0.2)	0.0 (-0.1 to 0.1)	0.0 (0.0 to 0.0)
Connecticut	1 to 4 years	0.3 (-0.4 to 0.9)	0.2 (-0.1 to 0.6)	0.3 (-0.4 to 1.0)	0.2 (-0.1 to 0.6)
Connecticut	10 to 14 years	-0.3 (-0.8 to 0.2)	-0.1 (-0.4 to 0.1)	-0.3 (-0.7 to 0.2)	-0.1 (-0.4 to 0.1)
Connecticut	15 to 19 years	1.5 (0.4 to 2.5)	0.3 (-0.3 to 0.9)	0.7 (0.2 to 1.2)	0.1 (-0.2 to 0.4)
Connecticut	20 to 24 years	3.0 (1.0 to 5.0)	1.2 (0.1 to 2.4)	1.0 (0.3 to 1.6)	0.4 (0.0 to 0.8)
Connecticut	25 to 29 years	2.3 (-0.3 to 5.0)	0.1 (-1.3 to 1.5)	0.9 (-0.1 to 1.9)	0.0 (-0.5 to 0.6)
Connecticut	30 to 34 years	3.5 (1.3 to 5.6)	0.1 (-1.1 to 1.2)	2.1 (0.8 to 3.3)	0.0 (-0.7 to 0.7)
Connecticut	35 to 39 years	3.5 (1.7 to 5.2)	0.4 (-0.6 to 1.3)	2.1 (1.0 to 3.1)	0.2 (-0.4 to 0.8)
Connecticut	40 to 44 years	3.1 (1.5 to 4.7)	0.7 (-0.2 to 1.5)	2.2 (1.0 to 3.3)	0.5 (-0.1 to 1.1)
Connecticut	45 to 49 years	3.2 (1.6 to 4.7)	0.9 (0.1 to 1.8)	1.9 (1.0 to 2.9)	0.6 (0.1 to 1.1)
Connecticut	5 to 9 years	0.0 (-0.7 to 0.6)	0.1 (-0.3 to 0.4)	0.0 (-0.6 to 0.6)	0.1 (-0.3 to 0.4)
Connecticut	50 to 54 years	2.4 (0.9 to 4.0)	1.0 (0.1 to 1.8)	1.2 (0.4 to 1.9)	0.5 (0.1 to 0.9)
Connecticut	55 to 59 years	1.0 (-0.5 to 2.5)	0.3 (-0.5 to 1.2)	0.6 (-0.3 to 1.4)	0.2 (-0.3 to 0.6)
Connecticut	60 to 64 years	1.4 (0.1 to 2.6)	0.7 (0.0 to 1.4)	1.1 (0.1 to 2.1)	0.5 (0.0 to 1.1)

© 2021 Weaver MR et al. *JAMA Network Open*.

Connecticut	65 to 69 years	0.6 (-0.6 to 1.7)	0.2 (-0.4 to 0.8)	0.2 (-0.2 to 0.5)	0.1 (-0.1 to 0.2)
Connecticut	70 to 74 years	-0.5 (-1.9 to 0.8)	-0.9 (-1.6 to -0.1)	-0.3 (-1.2 to 0.5)	-0.5 (-1.0 to -0.1)
Delaware	0 to 11 months	-0.2 (-1.2 to 0.9)	0.0 (-0.6 to 0.5)	-0.2 (-1.3 to 1.0)	0.0 (-0.6 to 0.6)
Delaware	1 to 4 years	0.4 (-0.5 to 1.3)	0.3 (-0.2 to 0.8)	0.3 (-0.4 to 1.0)	0.2 (-0.1 to 0.6)
Delaware	10 to 14 years	-1.2 (-3.1 to 0.8)	-0.6 (-1.7 to 0.4)	-0.7 (-2.0 to 0.5)	-0.4 (-1.0 to 0.3)
Delaware	15 to 19 years	1.3 (0.3 to 2.3)	0.2 (-0.3 to 0.8)	0.7 (0.2 to 1.2)	0.1 (-0.2 to 0.4)
Delaware	20 to 24 years	3.7 (1.2 to 6.1)	1.5 (0.2 to 2.8)	1.4 (0.5 to 2.4)	0.6 (0.1 to 1.1)
Delaware	25 to 29 years	2.6 (-0.4 to 5.7)	0.1 (-1.5 to 1.8)	1.3 (-0.2 to 2.8)	0.1 (-0.7 to 0.9)
Delaware	30 to 34 years	3.1 (1.2 to 5.0)	0.1 (-1.0 to 1.1)	2.5 (1.0 to 4.1)	0.0 (-0.8 to 0.9)
Delaware	35 to 39 years	3.3 (1.6 to 4.9)	0.3 (-0.6 to 1.2)	2.8 (1.4 to 4.3)	0.3 (-0.5 to 1.1)
Delaware	40 to 44 years	3.5 (1.7 to 5.4)	0.8 (-0.2 to 1.8)	2.6 (1.3 to 4.0)	0.6 (-0.2 to 1.3)
Delaware	45 to 49 years	3.3 (1.7 to 4.9)	1.0 (0.1 to 1.9)	1.7 (0.9 to 2.5)	0.5 (0.0 to 1.0)
Delaware	5 to 9 years	-0.1 (-1.1 to 1.0)	0.1 (-0.5 to 0.6)	0.0 (-0.7 to 0.6)	0.1 (-0.3 to 0.4)
Delaware	50 to 54 years	3.2 (1.2 to 5.3)	1.3 (0.2 to 2.4)	1.8 (0.7 to 3.0)	0.7 (0.1 to 1.4)
Delaware	55 to 59 years	1.2 (-0.6 to 2.9)	0.4 (-0.6 to 1.3)	0.6 (-0.3 to 1.6)	0.2 (-0.3 to 0.7)
Delaware	60 to 64 years	1.3 (0.1 to 2.5)	0.6 (0.0 to 1.3)	1.1 (0.1 to 2.0)	0.5 (0.0 to 1.1)
Delaware	65 to 69 years	0.0 (0.0 to 0.0)	0.0 (0.0 to 0.0)	0.2 (-0.2 to 0.5)	0.1 (-0.1 to 0.2)
Delaware	70 to 74 years	-0.1 (-0.3 to 0.1)	-0.1 (-0.2 to 0.0)	-0.6 (-2.2 to 0.9)	-1.0 (-1.8 to -0.2)
District of Columbia	0 to 11 months	-0.1 (-0.4 to 0.3)	0.0 (-0.2 to 0.2)	0.0 (-0.3 to 0.2)	0.0 (-0.1 to 0.1)
District of Columbia	1 to 4 years	0.1 (-0.1 to 0.3)	0.1 (0.0 to 0.2)	0.2 (-0.3 to 0.7)	0.2 (-0.1 to 0.4)
District of Columbia	10 to 14 years	-0.3 (-0.7 to 0.2)	-0.1 (-0.4 to 0.1)	-0.5 (-1.2 to 0.3)	-0.2 (-0.7 to 0.2)
District of Columbia	15 to 19 years	1.1 (0.3 to 1.9)	0.2 (-0.3 to 0.7)	0.7 (0.2 to 1.2)	0.1 (-0.2 to 0.4)
District of Columbia	20 to 24 years	1.4 (0.5 to 2.4)	0.6 (0.1 to 1.1)	0.8 (0.3 to 1.4)	0.3 (0.0 to 0.6)
District of Columbia	25 to 29 years	1.4 (-0.2 to 3.1)	0.1 (-0.8 to 1.0)	0.6 (-0.1 to 1.3)	0.0 (-0.4 to 0.4)
District of Columbia	30 to 34 years	2.0 (0.8 to 3.3)	0.0 (-0.6 to 0.7)	0.7 (0.3 to 1.1)	0.0 (-0.2 to 0.2)
District of Columbia	35 to 39 years	2.6 (1.3 to 3.8)	0.3 (-0.4 to 1.0)	1.2 (0.6 to 1.8)	0.1 (-0.2 to 0.5)
District of Columbia	40 to 44 years	2.2 (1.1 to 3.4)	0.5 (-0.1 to 1.1)	1.6 (0.8 to 2.5)	0.4 (-0.1 to 0.8)
District of Columbia	45 to 49 years	2.8 (1.4 to 4.1)	0.8 (0.1 to 1.6)	2.2 (1.1 to 3.2)	0.6 (0.1 to 1.2)
District of Columbia	5 to 9 years	0.0 (-0.3 to 0.3)	0.0 (-0.1 to 0.2)	-0.1 (-1.1 to 0.9)	0.1 (-0.4 to 0.6)

© 2021 Weaver MR et al. *JAMA Network Open*.

District of Columbia	50 to 54 years	2.6 (1.0 to 4.3)	1.1 (0.1 to 2.0)	1.2 (0.4 to 1.9)	0.5 (0.1 to 0.9)
District of Columbia	55 to 59 years	1.1 (-0.5 to 2.7)	0.3 (-0.5 to 1.2)	0.4 (-0.2 to 0.9)	0.1 (-0.2 to 0.4)
District of Columbia	60 to 64 years	1.2 (0.1 to 2.4)	0.6 (0.0 to 1.3)	0.6 (0.0 to 1.2)	0.3 (0.0 to 0.6)
District of Columbia	65 to 69 years	0.8 (-0.7 to 2.3)	0.3 (-0.5 to 1.1)	0.4 (-0.3 to 1.0)	0.1 (-0.3 to 0.5)
District of Columbia	70 to 74 years	-1.4 (-4.9 to 2.1)	-2.2 (-4.1 to -0.4)	-0.3 (-1.2 to 0.5)	-0.6 (-1.0 to -0.1)
Florida	0 to 11 months	-0.4 (-2.8 to 2.1)	-0.1 (-1.4 to 1.2)	-0.2 (-1.3 to 1.0)	0.0 (-0.6 to 0.6)
Florida	1 to 4 years	0.9 (-1.3 to 3.2)	0.8 (-0.4 to 2.0)	0.5 (-0.6 to 1.6)	0.4 (-0.2 to 1.0)
Florida	10 to 14 years	-1.8 (-4.9 to 1.2)	-1.0 (-2.6 to 0.7)	-0.8 (-2.1 to 0.5)	-0.4 (-1.1 to 0.3)
Florida	15 to 19 years	4.3 (1.1 to 7.4)	0.8 (-1.0 to 2.6)	2.4 (0.6 to 4.2)	0.4 (-0.6 to 1.4)
Florida	20 to 24 years	6.1 (2.1 to 10.2)	2.5 (0.3 to 4.8)	3.2 (1.1 to 5.3)	1.3 (0.1 to 2.5)
Florida	25 to 29 years	4.3 (-0.6 to 9.3)	0.2 (-2.5 to 2.9)	2.6 (-0.4 to 5.5)	0.1 (-1.5 to 1.7)
Florida	30 to 34 years	7.2 (2.8 to 11.6)	0.1 (-2.3 to 2.6)	4.7 (1.8 to 7.5)	0.1 (-1.5 to 1.7)
Florida	35 to 39 years	8.2 (4.1 to 12.2)	0.8 (-1.4 to 3.1)	5.5 (2.8 to 8.2)	0.6 (-1.0 to 2.1)
Florida	40 to 44 years	8.2 (4.0 to 12.4)	1.8 (-0.5 to 4.1)	5.2 (2.5 to 7.8)	1.1 (-0.3 to 2.6)
Florida	45 to 49 years	8.4 (4.3 to 12.6)	2.5 (0.2 to 4.8)	5.5 (2.8 to 8.1)	1.6 (0.2 to 3.1)
Florida	5 to 9 years	-0.2 (-2.9 to 2.5)	0.3 (-1.2 to 1.7)	-0.1 (-1.3 to 1.1)	0.1 (-0.5 to 0.8)
Florida	50 to 54 years	6.3 (2.3 to 10.3)	2.5 (0.3 to 4.7)	4.3 (1.5 to 7.0)	1.7 (0.2 to 3.2)
Florida	55 to 59 years	2.6 (-1.3 to 6.6)	0.8 (-1.3 to 3.0)	1.8 (-0.9 to 4.4)	0.5 (-0.9 to 2.0)
Florida	60 to 64 years	3.7 (0.3 to 7.2)	1.9 (0.0 to 3.8)	2.7 (0.2 to 5.1)	1.3 (0.0 to 2.7)
Florida	65 to 69 years	0.8 (-0.7 to 2.3)	0.3 (-0.6 to 1.1)	0.8 (-0.7 to 2.3)	0.3 (-0.6 to 1.1)
Florida	70 to 74 years	-0.7 (-2.4 to 1.0)	-1.1 (-2.0 to -0.2)	-0.6 (-2.1 to 0.9)	-1.0 (-1.7 to -0.2)
Georgia	0 to 11 months	-0.3 (-1.9 to 1.4)	0.0 (-0.9 to 0.8)	-0.3 (-1.9 to 1.4)	0.0 (-0.9 to 0.8)
Georgia	1 to 4 years	0.8 (-1.1 to 2.7)	0.7 (-0.4 to 1.7)	0.6 (-0.8 to 1.9)	0.5 (-0.3 to 1.2)
Georgia	10 to 14 years	-1.4 (-3.7 to 0.9)	-0.7 (-2.0 to 0.5)	-0.9 (-2.3 to 0.6)	-0.4 (-1.2 to 0.3)
Georgia	15 to 19 years	3.7 (0.9 to 6.4)	0.7 (-0.9 to 2.2)	2.2 (0.6 to 3.8)	0.4 (-0.5 to 1.3)
Georgia	20 to 24 years	6.0 (2.0 to 10.0)	2.5 (0.3 to 4.7)	3.3 (1.1 to 5.5)	1.4 (0.1 to 2.6)
Georgia	25 to 29 years	4.1 (-0.6 to 8.8)	0.2 (-2.3 to 2.7)	2.7 (-0.4 to 5.8)	0.1 (-1.5 to 1.8)
Georgia	30 to 34 years	6.8 (2.6 to 10.9)	0.1 (-2.2 to 2.4)	4.8 (1.9 to 7.8)	0.1 (-1.5 to 1.7)
Georgia	35 to 39 years	7.0 (3.5 to 10.5)	0.7 (-1.2 to 2.7)	5.7 (2.8 to 8.5)	0.6 (-1.0 to 2.2)

© 2021 Weaver MR et al. *JAMA Network Open*.

Georgia	40 to 44 years	6.6 (3.2 to 10.0)	1.4 (-0.4 to 3.3)	5.2 (2.5 to 7.8)	1.1 (-0.3 to 2.6)
Georgia	45 to 49 years	6.9 (3.5 to 10.3)	2.1 (0.2 to 3.9)	4.4 (2.3 to 6.6)	1.3 (0.1 to 2.5)
Georgia	5 to 9 years	-0.1 (-2.1 to 1.9)	0.2 (-0.9 to 1.3)	-0.1 (-1.4 to 1.2)	0.1 (-0.6 to 0.8)
Georgia	50 to 54 years	5.3 (1.9 to 8.6)	2.1 (0.3 to 4.0)	3.6 (1.3 to 5.9)	1.5 (0.2 to 2.7)
Georgia	55 to 59 years	2.2 (-1.1 to 5.4)	0.7 (-1.1 to 2.5)	1.6 (-0.8 to 4.1)	0.5 (-0.8 to 1.8)
Georgia	60 to 64 years	3.2 (0.2 to 6.2)	1.6 (0.0 to 3.3)	2.3 (0.2 to 4.5)	1.2 (0.0 to 2.3)
Georgia	65 to 69 years	0.4 (-0.4 to 1.2)	0.1 (-0.3 to 0.6)	0.5 (-0.5 to 1.6)	0.2 (-0.4 to 0.7)
Georgia	70 to 74 years	-0.5 (-1.7 to 0.7)	-0.8 (-1.4 to -0.1)	-0.2 (-0.7 to 0.3)	-0.3 (-0.6 to -0.1)
Hawaii	0 to 11 months	-0.2 (-1.7 to 1.2)	0.0 (-0.8 to 0.7)	-0.2 (-1.1 to 0.8)	0.0 (-0.6 to 0.5)
Hawaii	1 to 4 years	0.2 (-0.3 to 0.7)	0.2 (-0.1 to 0.4)	0.1 (-0.1 to 0.2)	0.1 (0.0 to 0.1)
Hawaii	10 to 14 years	-0.5 (-1.3 to 0.3)	-0.3 (-0.7 to 0.2)	-0.3 (-0.8 to 0.2)	-0.2 (-0.4 to 0.1)
Hawaii	15 to 19 years	1.8 (0.5 to 3.2)	0.3 (-0.4 to 1.1)	0.9 (0.2 to 1.6)	0.2 (-0.2 to 0.6)
Hawaii	20 to 24 years	1.7 (0.6 to 2.8)	0.7 (0.1 to 1.3)	0.7 (0.2 to 1.1)	0.3 (0.0 to 0.5)
Hawaii	25 to 29 years	1.6 (-0.2 to 3.4)	0.1 (-0.9 to 1.1)	0.7 (-0.1 to 1.5)	0.0 (-0.4 to 0.5)
Hawaii	30 to 34 years	1.8 (0.7 to 2.9)	0.0 (-0.6 to 0.6)	0.8 (0.3 to 1.3)	0.0 (-0.3 to 0.3)
Hawaii	35 to 39 years	2.4 (1.2 to 3.6)	0.2 (-0.4 to 0.9)	1.2 (0.6 to 1.8)	0.1 (-0.2 to 0.5)
Hawaii	40 to 44 years	2.7 (1.3 to 4.1)	0.6 (-0.2 to 1.4)	1.5 (0.7 to 2.2)	0.3 (-0.1 to 0.7)
Hawaii	45 to 49 years	2.6 (1.3 to 3.9)	0.8 (0.1 to 1.5)	1.1 (0.6 to 1.6)	0.3 (0.0 to 0.6)
Hawaii	5 to 9 years	-0.1 (-0.9 to 0.8)	0.1 (-0.4 to 0.5)	0.0 (-0.5 to 0.4)	0.0 (-0.2 to 0.3)
Hawaii	50 to 54 years	2.7 (1.0 to 4.4)	1.1 (0.1 to 2.0)	0.8 (0.3 to 1.4)	0.3 (0.0 to 0.6)
Hawaii	55 to 59 years	0.8 (-0.4 to 2.0)	0.3 (-0.4 to 0.9)	0.8 (-0.4 to 1.9)	0.2 (-0.4 to 0.8)
Hawaii	60 to 64 years	1.9 (0.1 to 3.7)	1.0 (0.0 to 1.9)	1.2 (0.1 to 2.2)	0.6 (0.0 to 1.2)
Hawaii	65 to 69 years	1.1 (-1.0 to 3.2)	0.4 (-0.8 to 1.5)	0.2 (-0.2 to 0.7)	0.1 (-0.2 to 0.3)
Hawaii	70 to 74 years	-1.0 (-3.6 to 1.5)	-1.6 (-3.0 to -0.3)	-0.1 (-0.5 to 0.2)	-0.2 (-0.4 to 0.0)
Idaho	0 to 11 months	-0.6 (-4.1 to 3.0)	-0.1 (-2.0 to 1.8)	-0.3 (-2.2 to 1.6)	-0.1 (-1.1 to 1.0)
Idaho	1 to 4 years	1.0 (-1.4 to 3.3)	0.8 (-0.4 to 2.1)	0.3 (-0.4 to 0.9)	0.2 (-0.1 to 0.5)
Idaho	10 to 14 years	-1.3 (-3.5 to 0.9)	-0.7 (-1.9 to 0.5)	-1.0 (-2.7 to 0.7)	-0.5 (-1.4 to 0.4)
Idaho	15 to 19 years	3.5 (0.9 to 6.1)	0.6 (-0.8 to 2.1)	1.5 (0.4 to 2.5)	0.3 (-0.3 to 0.9)
Idaho	20 to 24 years	5.2 (1.8 to 8.7)	2.1 (0.2 to 4.1)	2.5 (0.8 to 4.1)	1.0 (0.1 to 1.9)

© 2021 Weaver MR et al. *JAMA Network Open*.

Idaho	25 to 29 years	3.8 (-0.6 to 8.2)	0.2 (-2.2 to 2.6)	2.0 (-0.3 to 4.3)	0.1 (-1.2 to 1.3)
Idaho	30 to 34 years	5.3 (2.0 to 8.5)	0.1 (-1.7 to 1.9)	4.0 (1.5 to 6.5)	0.1 (-1.3 to 1.4)
Idaho	35 to 39 years	6.0 (3.0 to 8.9)	0.6 (-1.0 to 2.3)	4.3 (2.2 to 6.5)	0.4 (-0.8 to 1.6)
Idaho	40 to 44 years	6.7 (3.3 to 10.2)	1.5 (-0.4 to 3.4)	3.9 (1.9 to 5.9)	0.8 (-0.3 to 1.9)
Idaho	45 to 49 years	7.0 (3.6 to 10.5)	2.1 (0.2 to 4.0)	4.8 (2.5 to 7.2)	1.4 (0.1 to 2.7)
Idaho	5 to 9 years	-0.1 (-2.4 to 2.1)	0.2 (-1.0 to 1.4)	-0.1 (-1.2 to 1.0)	0.1 (-0.5 to 0.7)
Idaho	50 to 54 years	4.4 (1.6 to 7.2)	1.8 (0.2 to 3.3)	3.0 (1.1 to 5.0)	1.2 (0.2 to 2.3)
Idaho	55 to 59 years	2.0 (-1.0 to 5.0)	0.6 (-1.0 to 2.3)	1.5 (-0.7 to 3.7)	0.5 (-0.8 to 1.7)
Idaho	60 to 64 years	3.8 (0.3 to 7.2)	1.9 (0.0 to 3.8)	2.7 (0.2 to 5.3)	1.4 (0.0 to 2.8)
Idaho	65 to 69 years	0.1 (-0.1 to 0.4)	0.0 (-0.1 to 0.2)	0.2 (-0.2 to 0.6)	0.1 (-0.2 to 0.3)
Idaho	70 to 74 years	-0.2 (-0.8 to 0.3)	-0.3 (-0.6 to -0.1)	0.0 (0.0 to 0.0)	0.0 (0.0 to 0.0)
Illinois	0 to 11 months	-0.1 (-1.0 to 0.7)	0.0 (-0.5 to 0.4)	-0.1 (-0.7 to 0.5)	0.0 (-0.4 to 0.3)
Illinois	1 to 4 years	0.4 (-0.5 to 1.2)	0.3 (-0.2 to 0.8)	0.2 (-0.3 to 0.7)	0.2 (-0.1 to 0.4)
Illinois	10 to 14 years	-0.7 (-1.8 to 0.4)	-0.3 (-0.9 to 0.2)	-0.4 (-1.0 to 0.2)	-0.2 (-0.5 to 0.1)
Illinois	15 to 19 years	2.3 (0.6 to 4.0)	0.4 (-0.5 to 1.4)	0.9 (0.2 to 1.6)	0.2 (-0.2 to 0.6)
Illinois	20 to 24 years	4.4 (1.5 to 7.3)	1.8 (0.2 to 3.4)	1.6 (0.5 to 2.6)	0.6 (0.1 to 1.2)
Illinois	25 to 29 years	3.0 (-0.4 to 6.4)	0.1 (-1.7 to 2.0)	1.3 (-0.2 to 2.7)	0.1 (-0.7 to 0.8)
Illinois	30 to 34 years	4.8 (1.8 to 7.7)	0.1 (-1.5 to 1.7)	2.4 (0.9 to 3.8)	0.0 (-0.8 to 0.8)
Illinois	35 to 39 years	5.3 (2.7 to 7.9)	0.5 (-0.9 to 2.0)	2.8 (1.4 to 4.2)	0.3 (-0.5 to 1.1)
Illinois	40 to 44 years	4.7 (2.3 to 7.1)	1.0 (-0.3 to 2.3)	3.0 (1.4 to 4.5)	0.7 (-0.2 to 1.5)
Illinois	45 to 49 years	5.1 (2.6 to 7.5)	1.5 (0.1 to 2.9)	2.6 (1.3 to 3.8)	0.8 (0.1 to 1.5)
Illinois	5 to 9 years	-0.1 (-0.9 to 0.8)	0.1 (-0.4 to 0.5)	0.0 (-0.5 to 0.4)	0.0 (-0.2 to 0.3)
Illinois	50 to 54 years	4.1 (1.5 to 6.7)	1.7 (0.2 to 3.1)	1.9 (0.7 to 3.1)	0.8 (0.1 to 1.4)
Illinois	55 to 59 years	1.7 (-0.8 to 4.1)	0.5 (-0.8 to 1.9)	0.8 (-0.4 to 1.9)	0.2 (-0.4 to 0.9)
Illinois	60 to 64 years	2.6 (0.2 to 5.0)	1.3 (0.0 to 2.6)	1.2 (0.1 to 2.4)	0.6 (0.0 to 1.2)
Illinois	65 to 69 years	0.9 (-0.8 to 2.6)	0.3 (-0.6 to 1.2)	0.6 (-0.6 to 1.7)	0.2 (-0.4 to 0.8)
Illinois	70 to 74 years	-0.5 (-1.8 to 0.8)	-0.8 (-1.5 to -0.1)	-0.3 (-1.1 to 0.5)	-0.5 (-1.0 to -0.1)
Indiana	0 to 11 months	-0.3 (-1.9 to 1.3)	0.0 (-0.9 to 0.8)	-0.3 (-1.9 to 1.4)	0.0 (-0.9 to 0.8)
Indiana	1 to 4 years	0.9 (-1.2 to 2.9)	0.7 (-0.4 to 1.8)	0.5 (-0.7 to 1.7)	0.4 (-0.2 to 1.0)

© 2021 Weaver MR et al. *JAMA Network Open*.

Indiana	10 to 14 years	-1.1 (-3.0 to 0.8)	-0.6 (-1.6 to 0.4)	-0.7 (-1.8 to 0.5)	-0.4 (-1.0 to 0.3)
Indiana	15 to 19 years	2.7 (0.7 to 4.8)	0.5 (-0.7 to 1.6)	1.5 (0.4 to 2.6)	0.3 (-0.3 to 0.9)
Indiana	20 to 24 years	4.7 (1.6 to 7.8)	1.9 (0.2 to 3.6)	1.6 (0.6 to 2.7)	0.7 (0.1 to 1.3)
Indiana	25 to 29 years	3.4 (-0.5 to 7.4)	0.2 (-2.0 to 2.3)	1.7 (-0.3 to 3.7)	0.1 (-1.0 to 1.2)
Indiana	30 to 34 years	4.9 (1.9 to 7.8)	0.1 (-1.6 to 1.7)	3.2 (1.2 to 5.2)	0.1 (-1.0 to 1.1)
Indiana	35 to 39 years	5.7 (2.9 to 8.6)	0.6 (-1.0 to 2.2)	3.3 (1.6 to 4.9)	0.3 (-0.6 to 1.2)
Indiana	40 to 44 years	5.1 (2.5 to 7.7)	1.1 (-0.3 to 2.6)	3.2 (1.6 to 4.8)	0.7 (-0.2 to 1.6)
Indiana	45 to 49 years	5.3 (2.7 to 7.8)	1.6 (0.2 to 3.0)	3.0 (1.5 to 4.5)	0.9 (0.1 to 1.7)
Indiana	5 to 9 years	-0.1 (-2.0 to 1.7)	0.2 (-0.8 to 1.2)	-0.1 (-1.3 to 1.2)	0.1 (-0.6 to 0.8)
Indiana	50 to 54 years	3.6 (1.3 to 5.9)	1.5 (0.2 to 2.7)	2.3 (0.8 to 3.8)	0.9 (0.1 to 1.7)
Indiana	55 to 59 years	1.5 (-0.7 to 3.7)	0.5 (-0.8 to 1.7)	0.9 (-0.4 to 2.2)	0.3 (-0.4 to 1.0)
Indiana	60 to 64 years	2.4 (0.2 to 4.7)	1.2 (0.0 to 2.4)	1.4 (0.1 to 2.8)	0.7 (0.0 to 1.5)
Indiana	65 to 69 years	0.2 (-0.2 to 0.6)	0.1 (-0.2 to 0.3)	0.3 (-0.3 to 1.0)	0.1 (-0.2 to 0.5)
Indiana	70 to 74 years	-0.1 (-0.2 to 0.1)	-0.1 (-0.1 to 0.0)	-0.3 (-1.0 to 0.4)	-0.5 (-0.9 to -0.1)
Iowa	0 to 11 months	-0.1 (-0.9 to 0.7)	0.0 (-0.5 to 0.4)	-0.1 (-0.9 to 0.6)	0.0 (-0.4 to 0.4)
Iowa	1 to 4 years	0.3 (-0.4 to 1.0)	0.2 (-0.1 to 0.6)	0.2 (-0.2 to 0.6)	0.1 (-0.1 to 0.4)
Iowa	10 to 14 years	-0.8 (-2.0 to 0.5)	-0.4 (-1.1 to 0.3)	-0.3 (-0.7 to 0.2)	-0.1 (-0.4 to 0.1)
Iowa	15 to 19 years	1.8 (0.5 to 3.2)	0.3 (-0.4 to 1.1)	0.7 (0.2 to 1.2)	0.1 (-0.2 to 0.4)
Iowa	20 to 24 years	3.4 (1.1 to 5.6)	1.4 (0.1 to 2.6)	1.1 (0.4 to 1.8)	0.4 (0.0 to 0.8)
Iowa	25 to 29 years	2.2 (-0.3 to 4.8)	0.1 (-1.3 to 1.5)	0.8 (-0.1 to 1.6)	0.0 (-0.4 to 0.5)
Iowa	30 to 34 years	3.1 (1.2 to 5.0)	0.1 (-1.0 to 1.1)	1.6 (0.6 to 2.6)	0.0 (-0.5 to 0.6)
Iowa	35 to 39 years	3.1 (1.5 to 4.6)	0.3 (-0.5 to 1.2)	1.9 (1.0 to 2.9)	0.2 (-0.3 to 0.7)
Iowa	40 to 44 years	3.1 (1.5 to 4.7)	0.7 (-0.2 to 1.5)	1.6 (0.8 to 2.4)	0.3 (-0.1 to 0.8)
Iowa	45 to 49 years	3.9 (2.0 to 5.7)	1.1 (0.1 to 2.2)	1.9 (1.0 to 2.8)	0.6 (0.1 to 1.0)
Iowa	5 to 9 years	0.0 (-0.7 to 0.7)	0.1 (-0.3 to 0.4)	0.0 (-0.5 to 0.4)	0.0 (-0.2 to 0.3)
Iowa	50 to 54 years	2.6 (1.0 to 4.3)	1.1 (0.1 to 2.0)	1.1 (0.4 to 1.7)	0.4 (0.1 to 0.8)
Iowa	55 to 59 years	1.0 (-0.5 to 2.5)	0.3 (-0.5 to 1.2)	0.6 (-0.3 to 1.4)	0.2 (-0.3 to 0.6)
Iowa	60 to 64 years	1.2 (0.1 to 2.3)	0.6 (0.0 to 1.2)	0.7 (0.1 to 1.4)	0.4 (0.0 to 0.7)
Iowa	65 to 69 years	0.3 (-0.3 to 0.8)	0.1 (-0.2 to 0.4)	0.1 (-0.1 to 0.2)	0.0 (0.0 to 0.1)

© 2021 Weaver MR et al. *JAMA Network Open*.

Iowa	70 to 74 years	0.0 (0.0 to 0.0)	0.0 (0.0 to 0.0)	0.0 (-0.2 to 0.1)	-0.1 (-0.1 to 0.0)
Kansas	0 to 11 months	-0.3 (-2.0 to 1.5)	0.0 (-1.0 to 0.9)	-0.1 (-1.0 to 0.7)	0.0 (-0.5 to 0.4)
Kansas	1 to 4 years	0.6 (-0.9 to 2.1)	0.5 (-0.3 to 1.3)	0.4 (-0.5 to 1.3)	0.3 (-0.2 to 0.8)
Kansas	10 to 14 years	-1.1 (-3.0 to 0.7)	-0.6 (-1.6 to 0.4)	-0.6 (-1.5 to 0.4)	-0.3 (-0.8 to 0.2)
Kansas	15 to 19 years	2.5 (0.6 to 4.3)	0.4 (-0.6 to 1.5)	1.5 (0.4 to 2.7)	0.3 (-0.4 to 0.9)
Kansas	20 to 24 years	4.3 (1.5 to 7.2)	1.8 (0.2 to 3.4)	2.2 (0.7 to 3.7)	0.9 (0.1 to 1.7)
Kansas	25 to 29 years	3.1 (-0.5 to 6.6)	0.1 (-1.8 to 2.0)	1.7 (-0.3 to 3.7)	0.1 (-1.0 to 1.2)
Kansas	30 to 34 years	4.9 (1.9 to 8.0)	0.1 (-1.6 to 1.8)	3.3 (1.3 to 5.3)	0.1 (-1.0 to 1.2)
Kansas	35 to 39 years	5.6 (2.8 to 8.3)	0.6 (-1.0 to 2.1)	3.5 (1.7 to 5.2)	0.4 (-0.6 to 1.3)
Kansas	40 to 44 years	4.9 (2.4 to 7.5)	1.1 (-0.3 to 2.5)	3.8 (1.8 to 5.7)	0.8 (-0.2 to 1.9)
Kansas	45 to 49 years	4.7 (2.4 to 7.0)	1.4 (0.1 to 2.7)	2.9 (1.5 to 4.4)	0.9 (0.1 to 1.7)
Kansas	5 to 9 years	-0.1 (-1.6 to 1.4)	0.1 (-0.7 to 0.9)	-0.1 (-1.2 to 1.0)	0.1 (-0.5 to 0.7)
Kansas	50 to 54 years	3.2 (1.2 to 5.3)	1.3 (0.2 to 2.5)	2.5 (0.9 to 4.1)	1.0 (0.1 to 1.9)
Kansas	55 to 59 years	1.2 (-0.6 to 2.9)	0.4 (-0.6 to 1.3)	0.8 (-0.4 to 2.0)	0.2 (-0.4 to 0.9)
Kansas	60 to 64 years	2.6 (0.2 to 5.0)	1.3 (0.0 to 2.6)	1.4 (0.1 to 2.6)	0.7 (0.0 to 1.4)
Kansas	65 to 69 years	0.2 (-0.2 to 0.6)	0.1 (-0.2 to 0.3)	0.3 (-0.3 to 0.8)	0.1 (-0.2 to 0.4)
Kansas	70 to 74 years	0.0 (-0.1 to 0.0)	0.0 (0.0 to 0.0)	-0.1 (-0.2 to 0.1)	-0.1 (-0.2 to 0.0)
Kentucky	0 to 11 months	-0.2 (-1.4 to 1.0)	0.0 (-0.7 to 0.6)	-0.1 (-0.8 to 0.6)	0.0 (-0.4 to 0.3)
Kentucky	1 to 4 years	0.5 (-0.8 to 1.8)	0.5 (-0.2 to 1.1)	0.2 (-0.3 to 0.7)	0.2 (-0.1 to 0.5)
Kentucky	10 to 14 years	-0.8 (-2.2 to 0.5)	-0.4 (-1.2 to 0.3)	-0.5 (-1.3 to 0.3)	-0.2 (-0.7 to 0.2)
Kentucky	15 to 19 years	2.8 (0.7 to 4.9)	0.5 (-0.7 to 1.7)	0.8 (0.2 to 1.4)	0.1 (-0.2 to 0.5)
Kentucky	20 to 24 years	4.9 (1.7 to 8.2)	2.0 (0.2 to 3.8)	1.3 (0.4 to 2.2)	0.5 (0.1 to 1.0)
Kentucky	25 to 29 years	3.4 (-0.5 to 7.3)	0.2 (-2.0 to 2.3)	1.3 (-0.2 to 2.9)	0.1 (-0.8 to 0.9)
Kentucky	30 to 34 years	5.5 (2.1 to 8.9)	0.1 (-1.8 to 2.0)	2.0 (0.8 to 3.3)	0.0 (-0.6 to 0.7)
Kentucky	35 to 39 years	6.3 (3.1 to 9.4)	0.6 (-1.1 to 2.4)	2.3 (1.2 to 3.5)	0.2 (-0.4 to 0.9)
Kentucky	40 to 44 years	5.9 (2.9 to 9.0)	1.3 (-0.4 to 3.0)	2.0 (1.0 to 3.1)	0.4 (-0.1 to 1.0)
Kentucky	45 to 49 years	5.6 (2.9 to 8.4)	1.7 (0.2 to 3.2)	2.1 (1.1 to 3.2)	0.6 (0.1 to 1.2)
Kentucky	5 to 9 years	-0.1 (-1.2 to 1.0)	0.1 (-0.5 to 0.7)	-0.1 (-0.8 to 0.7)	0.1 (-0.3 to 0.5)
Kentucky	50 to 54 years	4.1 (1.5 to 6.8)	1.7 (0.2 to 3.1)	1.4 (0.5 to 2.3)	0.6 (0.1 to 1.1)

© 2021 Weaver MR et al. *JAMA Network Open*.

Kentucky	55 to 59 years	1.7 (-0.8 to 4.2)	0.5 (-0.8 to 1.9)	0.6 (-0.3 to 1.4)	0.2 (-0.3 to 0.7)
Kentucky	60 to 64 years	2.3 (0.2 to 4.5)	1.2 (0.0 to 2.4)	0.9 (0.1 to 1.7)	0.4 (0.0 to 0.9)
Kentucky	65 to 69 years	0.2 (-0.2 to 0.6)	0.1 (-0.1 to 0.3)	0.2 (-0.2 to 0.5)	0.1 (-0.1 to 0.2)
Kentucky	70 to 74 years	0.0 (-0.1 to 0.1)	-0.1 (-0.1 to 0.0)	-0.1 (-0.5 to 0.2)	-0.2 (-0.4 to 0.0)
Louisiana	0 to 11 months	-0.2 (-1.2 to 0.9)	0.0 (-0.6 to 0.5)	-0.1 (-0.7 to 0.5)	0.0 (-0.3 to 0.3)
Louisiana	1 to 4 years	0.4 (-0.6 to 1.4)	0.4 (-0.2 to 0.9)	0.2 (-0.3 to 0.8)	0.2 (-0.1 to 0.5)
Louisiana	10 to 14 years	-0.8 (-2.2 to 0.5)	-0.4 (-1.2 to 0.3)	-0.5 (-1.3 to 0.3)	-0.3 (-0.7 to 0.2)
Louisiana	15 to 19 years	3.2 (0.8 to 5.5)	0.6 (-0.8 to 1.9)	1.6 (0.4 to 2.8)	0.3 (-0.4 to 0.9)
Louisiana	20 to 24 years	5.2 (1.8 to 8.7)	2.2 (0.2 to 4.1)	2.7 (0.9 to 4.6)	1.1 (0.1 to 2.1)
Louisiana	25 to 29 years	3.6 (-0.5 to 7.8)	0.2 (-2.1 to 2.4)	2.2 (-0.3 to 4.7)	0.1 (-1.2 to 1.5)
Louisiana	30 to 34 years	6.4 (2.5 to 10.4)	0.1 (-2.1 to 2.3)	4.1 (1.6 to 6.7)	0.1 (-1.3 to 1.5)
Louisiana	35 to 39 years	7.2 (3.6 to 10.9)	0.7 (-1.3 to 2.7)	4.9 (2.5 to 7.4)	0.5 (-0.9 to 1.9)
Louisiana	40 to 44 years	6.9 (3.4 to 10.5)	1.5 (-0.5 to 3.5)	4.5 (2.2 to 6.8)	1.0 (-0.3 to 2.2)
Louisiana	45 to 49 years	7.5 (3.8 to 11.1)	2.2 (0.2 to 4.2)	4.8 (2.5 to 7.2)	1.4 (0.1 to 2.7)
Louisiana	5 to 9 years	-0.1 (-1.2 to 1.1)	0.1 (-0.5 to 0.7)	0.0 (-0.7 to 0.6)	0.1 (-0.3 to 0.4)
Louisiana	50 to 54 years	5.5 (2.0 to 9.0)	2.2 (0.3 to 4.2)	3.6 (1.3 to 5.9)	1.5 (0.2 to 2.7)
Louisiana	55 to 59 years	2.2 (-1.0 to 5.3)	0.7 (-1.1 to 2.4)	1.5 (-0.7 to 3.8)	0.5 (-0.8 to 1.7)
Louisiana	60 to 64 years	3.1 (0.2 to 5.9)	1.5 (0.0 to 3.1)	1.7 (0.1 to 3.3)	0.9 (0.0 to 1.7)
Louisiana	65 to 69 years	0.6 (-0.6 to 1.7)	0.2 (-0.4 to 0.8)	0.2 (-0.2 to 0.5)	0.1 (-0.1 to 0.2)
Louisiana	70 to 74 years	-0.1 (-0.3 to 0.1)	-0.1 (-0.3 to 0.0)	-0.2 (-0.6 to 0.2)	-0.3 (-0.5 to 0.0)
Maine	0 to 11 months	0.0 (-0.3 to 0.2)	0.0 (-0.1 to 0.1)	0.0 (-0.2 to 0.2)	0.0 (-0.1 to 0.1)
Maine	1 to 4 years	0.2 (-0.3 to 0.8)	0.2 (-0.1 to 0.5)	0.4 (-0.6 to 1.4)	0.4 (-0.2 to 0.9)
Maine	10 to 14 years	-0.6 (-1.5 to 0.4)	-0.3 (-0.8 to 0.2)	-0.6 (-1.7 to 0.4)	-0.3 (-0.9 to 0.2)
Maine	15 to 19 years	1.8 (0.5 to 3.1)	0.3 (-0.4 to 1.1)	1.2 (0.3 to 2.2)	0.2 (-0.3 to 0.7)
Maine	20 to 24 years	3.5 (1.2 to 5.8)	1.4 (0.2 to 2.7)	1.9 (0.6 to 3.1)	0.8 (0.1 to 1.4)
Maine	25 to 29 years	2.4 (-0.4 to 5.2)	0.1 (-1.4 to 1.6)	1.6 (-0.2 to 3.5)	0.1 (-0.9 to 1.1)
Maine	30 to 34 years	4.2 (1.6 to 6.7)	0.1 (-1.3 to 1.5)	2.5 (1.0 to 4.0)	0.0 (-0.8 to 0.9)
Maine	35 to 39 years	3.7 (1.8 to 5.5)	0.4 (-0.6 to 1.4)	3.3 (1.7 to 5.0)	0.3 (-0.6 to 1.3)
Maine	40 to 44 years	4.0 (1.9 to 6.1)	0.9 (-0.3 to 2.0)	3.7 (1.8 to 5.7)	0.8 (-0.2 to 1.9)

© 2021 Weaver MR et al. *JAMA Network Open*.

Maine	45 to 49 years	4.0 (2.1 to 6.0)	1.2 (0.1 to 2.3)	3.8 (1.9 to 5.6)	1.1 (0.1 to 2.1)
Maine	5 to 9 years	0.0 (-0.7 to 0.6)	0.1 (-0.3 to 0.4)	-0.1 (-0.9 to 0.8)	0.1 (-0.4 to 0.5)
Maine	50 to 54 years	3.3 (1.2 to 5.4)	1.3 (0.2 to 2.5)	2.6 (0.9 to 4.3)	1.1 (0.1 to 2.0)
Maine	55 to 59 years	1.3 (-0.6 to 3.3)	0.4 (-0.7 to 1.5)	1.2 (-0.6 to 2.9)	0.4 (-0.6 to 1.3)
Maine	60 to 64 years	2.0 (0.2 to 3.9)	1.0 (0.0 to 2.1)	1.4 (0.1 to 2.6)	0.7 (0.0 to 1.4)
Maine	65 to 69 years	0.1 (-0.1 to 0.2)	0.0 (0.0 to 0.1)	0.1 (-0.1 to 0.2)	0.0 (-0.1 to 0.1)
Maine	70 to 74 years	-0.1 (-0.3 to 0.1)	-0.2 (-0.3 to 0.0)	-0.1 (-0.2 to 0.1)	-0.1 (-0.2 to 0.0)
Maryland	0 to 11 months	-0.1 (-0.9 to 0.7)	0.0 (-0.5 to 0.4)	-0.1 (-0.9 to 0.7)	0.0 (-0.4 to 0.4)
Maryland	1 to 4 years	0.4 (-0.6 to 1.5)	0.4 (-0.2 to 0.9)	0.3 (-0.4 to 1.0)	0.3 (-0.1 to 0.6)
Maryland	10 to 14 years	-0.7 (-1.9 to 0.5)	-0.4 (-1.0 to 0.3)	-0.4 (-1.0 to 0.3)	-0.2 (-0.6 to 0.1)
Maryland	15 to 19 years	1.9 (0.5 to 3.2)	0.3 (-0.4 to 1.1)	1.0 (0.3 to 1.8)	0.2 (-0.2 to 0.6)
Maryland	20 to 24 years	3.9 (1.3 to 6.5)	1.6 (0.2 to 3.0)	1.4 (0.5 to 2.4)	0.6 (0.1 to 1.1)
Maryland	25 to 29 years	2.5 (-0.4 to 5.4)	0.1 (-1.4 to 1.7)	1.3 (-0.2 to 2.7)	0.1 (-0.7 to 0.9)
Maryland	30 to 34 years	4.1 (1.6 to 6.6)	0.1 (-1.3 to 1.4)	2.4 (0.9 to 3.9)	0.0 (-0.8 to 0.9)
Maryland	35 to 39 years	4.4 (2.2 to 6.6)	0.4 (-0.8 to 1.7)	2.9 (1.5 to 4.4)	0.3 (-0.5 to 1.1)
Maryland	40 to 44 years	3.8 (1.8 to 5.8)	0.8 (-0.3 to 1.9)	2.3 (1.1 to 3.5)	0.5 (-0.2 to 1.2)
Maryland	45 to 49 years	3.8 (2.0 to 5.7)	1.1 (0.1 to 2.2)	2.1 (1.1 to 3.1)	0.6 (0.1 to 1.2)
Maryland	5 to 9 years	-0.1 (-1.0 to 0.9)	0.1 (-0.4 to 0.6)	0.0 (-0.7 to 0.6)	0.1 (-0.3 to 0.4)
Maryland	50 to 54 years	2.6 (0.9 to 4.2)	1.0 (0.1 to 1.9)	1.5 (0.6 to 2.5)	0.6 (0.1 to 1.2)
Maryland	55 to 59 years	1.2 (-0.6 to 2.9)	0.4 (-0.6 to 1.3)	0.6 (-0.3 to 1.6)	0.2 (-0.3 to 0.7)
Maryland	60 to 64 years	1.6 (0.1 to 3.1)	0.8 (0.0 to 1.6)	1.2 (0.1 to 2.2)	0.6 (0.0 to 1.2)
Maryland	65 to 69 years	0.9 (-0.8 to 2.6)	0.3 (-0.6 to 1.2)	0.4 (-0.4 to 1.1)	0.1 (-0.3 to 0.5)
Maryland	70 to 74 years	-0.9 (-3.1 to 1.3)	-1.4 (-2.6 to -0.2)	-0.5 (-1.6 to 0.7)	-0.7 (-1.3 to -0.1)
Massachusetts	0 to 11 months	-0.1 (-0.5 to 0.4)	0.0 (-0.3 to 0.2)	-0.1 (-0.4 to 0.3)	0.0 (-0.2 to 0.2)
Massachusetts	1 to 4 years	0.1 (-0.1 to 0.3)	0.1 (0.0 to 0.2)	0.1 (-0.2 to 0.4)	0.1 (-0.1 to 0.3)
Massachusetts	10 to 14 years	-0.2 (-0.5 to 0.1)	-0.1 (-0.3 to 0.1)	-0.1 (-0.3 to 0.1)	-0.1 (-0.2 to 0.0)
Massachusetts	15 to 19 years	0.6 (0.2 to 1.1)	0.1 (-0.2 to 0.4)	0.3 (0.1 to 0.6)	0.1 (-0.1 to 0.2)
Massachusetts	20 to 24 years	1.4 (0.5 to 2.4)	0.6 (0.1 to 1.1)	0.6 (0.2 to 1.0)	0.3 (0.0 to 0.5)
Massachusetts	25 to 29 years	1.1 (-0.2 to 2.4)	0.1 (-0.6 to 0.8)	0.5 (-0.1 to 1.1)	0.0 (-0.3 to 0.3)

© 2021 Weaver MR et al. *JAMA Network Open*.

Massachusetts	30 to 34 years	1.5 (0.6 to 2.4)	0.0 (-0.5 to 0.5)	0.9 (0.4 to 1.5)	0.0 (-0.3 to 0.3)
Massachusetts	35 to 39 years	1.7 (0.8 to 2.5)	0.2 (-0.3 to 0.6)	1.0 (0.5 to 1.4)	0.1 (-0.2 to 0.4)
Massachusetts	40 to 44 years	1.4 (0.7 to 2.2)	0.3 (-0.1 to 0.7)	0.9 (0.4 to 1.4)	0.2 (-0.1 to 0.5)
Massachusetts	45 to 49 years	1.6 (0.8 to 2.4)	0.5 (0.0 to 0.9)	1.0 (0.5 to 1.5)	0.3 (0.0 to 0.6)
Massachusetts	5 to 9 years	0.0 (-0.3 to 0.2)	0.0 (-0.1 to 0.2)	0.0 (-0.2 to 0.2)	0.0 (-0.1 to 0.1)
Massachusetts	50 to 54 years	1.3 (0.5 to 2.1)	0.5 (0.1 to 1.0)	0.8 (0.3 to 1.2)	0.3 (0.0 to 0.6)
Massachusetts	55 to 59 years	0.5 (-0.2 to 1.2)	0.2 (-0.2 to 0.6)	0.4 (-0.2 to 0.9)	0.1 (-0.2 to 0.4)
Massachusetts	60 to 64 years	0.5 (0.0 to 1.0)	0.2 (0.0 to 0.5)	0.4 (0.0 to 0.8)	0.2 (0.0 to 0.4)
Massachusetts	65 to 69 years	0.1 (-0.1 to 0.3)	0.0 (-0.1 to 0.1)	0.3 (-0.3 to 0.9)	0.1 (-0.2 to 0.4)
Massachusetts	70 to 74 years	-0.2 (-0.7 to 0.3)	-0.3 (-0.6 to -0.1)	-0.1 (-0.2 to 0.1)	-0.1 (-0.2 to 0.0)
Michigan	0 to 11 months	-0.2 (-1.2 to 0.8)	0.0 (-0.6 to 0.5)	-0.1 (-0.6 to 0.4)	0.0 (-0.3 to 0.3)
Michigan	1 to 4 years	0.4 (-0.6 to 1.5)	0.4 (-0.2 to 0.9)	0.3 (-0.4 to 1.0)	0.3 (-0.1 to 0.6)
Michigan	10 to 14 years	-0.5 (-1.4 to 0.3)	-0.3 (-0.7 to 0.2)	-0.3 (-0.9 to 0.2)	-0.2 (-0.5 to 0.1)
Michigan	15 to 19 years	1.7 (0.4 to 3.0)	0.3 (-0.4 to 1.0)	0.8 (0.2 to 1.3)	0.1 (-0.2 to 0.5)
Michigan	20 to 24 years	4.5 (1.5 to 7.4)	1.8 (0.2 to 3.5)	1.3 (0.5 to 2.2)	0.6 (0.1 to 1.0)
Michigan	25 to 29 years	3.2 (-0.5 to 6.8)	0.2 (-1.8 to 2.1)	1.3 (-0.2 to 2.7)	0.1 (-0.7 to 0.9)
Michigan	30 to 34 years	4.6 (1.8 to 7.4)	0.1 (-1.5 to 1.6)	2.2 (0.8 to 3.5)	0.0 (-0.7 to 0.8)
Michigan	35 to 39 years	4.6 (2.3 to 6.9)	0.5 (-0.8 to 1.8)	2.4 (1.2 to 3.6)	0.2 (-0.4 to 0.9)
Michigan	40 to 44 years	4.5 (2.2 to 6.9)	1.0 (-0.3 to 2.3)	2.3 (1.1 to 3.4)	0.5 (-0.1 to 1.1)
Michigan	45 to 49 years	5.0 (2.5 to 7.4)	1.5 (0.1 to 2.8)	2.0 (1.0 to 2.9)	0.6 (0.1 to 1.1)
Michigan	5 to 9 years	0.0 (-0.8 to 0.7)	0.1 (-0.3 to 0.5)	0.0 (-0.8 to 0.7)	0.1 (-0.3 to 0.5)
Michigan	50 to 54 years	3.6 (1.3 to 5.9)	1.5 (0.2 to 2.7)	1.5 (0.6 to 2.5)	0.6 (0.1 to 1.2)
Michigan	55 to 59 years	1.4 (-0.7 to 3.4)	0.4 (-0.7 to 1.5)	0.6 (-0.3 to 1.6)	0.2 (-0.3 to 0.7)
Michigan	60 to 64 years	1.9 (0.1 to 3.6)	0.9 (0.0 to 1.9)	0.9 (0.1 to 1.7)	0.5 (0.0 to 0.9)
Michigan	65 to 69 years	0.2 (-0.2 to 0.5)	0.1 (-0.1 to 0.3)	0.2 (-0.2 to 0.5)	0.1 (-0.1 to 0.3)
Michigan	70 to 74 years	-0.1 (-0.4 to 0.2)	-0.2 (-0.3 to 0.0)	-0.2 (-0.7 to 0.3)	-0.3 (-0.6 to -0.1)
Minnesota	0 to 11 months	-0.2 (-1.1 to 0.8)	0.0 (-0.6 to 0.5)	-0.2 (-1.6 to 1.1)	0.0 (-0.8 to 0.7)
Minnesota	1 to 4 years	0.6 (-0.9 to 2.1)	0.5 (-0.3 to 1.3)	0.2 (-0.3 to 0.8)	0.2 (-0.1 to 0.5)
Minnesota	10 to 14 years	-0.8 (-2.1 to 0.5)	-0.4 (-1.1 to 0.3)	-0.4 (-1.1 to 0.3)	-0.2 (-0.6 to 0.2)

© 2021 Weaver MR et al. *JAMA Network Open*.

Minnesota	15 to 19 years	1.7 (0.4 to 2.9)	0.3 (-0.4 to 1.0)	0.7 (0.2 to 1.2)	0.1 (-0.2 to 0.4)
Minnesota	20 to 24 years	3.4 (1.1 to 5.6)	1.4 (0.1 to 2.6)	1.0 (0.3 to 1.7)	0.4 (0.0 to 0.8)
Minnesota	25 to 29 years	1.9 (-0.3 to 4.1)	0.1 (-1.1 to 1.3)	0.8 (-0.1 to 1.6)	0.0 (-0.4 to 0.5)
Minnesota	30 to 34 years	2.5 (1.0 to 4.1)	0.0 (-0.8 to 0.9)	1.4 (0.5 to 2.3)	0.0 (-0.4 to 0.5)
Minnesota	35 to 39 years	3.1 (1.5 to 4.6)	0.3 (-0.5 to 1.2)	2.2 (1.1 to 3.2)	0.2 (-0.4 to 0.8)
Minnesota	40 to 44 years	2.5 (1.2 to 3.8)	0.6 (-0.2 to 1.3)	1.8 (0.9 to 2.7)	0.4 (-0.1 to 0.9)
Minnesota	45 to 49 years	2.7 (1.4 to 4.0)	0.8 (0.1 to 1.5)	1.4 (0.7 to 2.0)	0.4 (0.0 to 0.8)
Minnesota	5 to 9 years	-0.1 (-1.6 to 1.4)	0.1 (-0.7 to 1.0)	0.0 (-0.7 to 0.6)	0.1 (-0.3 to 0.4)
Minnesota	50 to 54 years	2.1 (0.8 to 3.5)	0.9 (0.1 to 1.6)	1.1 (0.4 to 1.8)	0.4 (0.1 to 0.8)
Minnesota	55 to 59 years	0.8 (-0.4 to 2.1)	0.3 (-0.4 to 0.9)	0.5 (-0.3 to 1.3)	0.2 (-0.3 to 0.6)
Minnesota	60 to 64 years	1.1 (0.1 to 2.2)	0.6 (0.0 to 1.1)	0.8 (0.1 to 1.5)	0.4 (0.0 to 0.8)
Minnesota	65 to 69 years	0.2 (-0.2 to 0.7)	0.1 (-0.2 to 0.3)	0.0 (0.0 to 0.1)	0.0 (0.0 to 0.1)
Minnesota	70 to 74 years	0.0 (-0.1 to 0.0)	-0.1 (-0.1 to 0.0)	0.0 (0.0 to 0.0)	0.0 (0.0 to 0.0)
Mississippi	0 to 11 months	-0.2 (-1.4 to 1.0)	0.0 (-0.7 to 0.6)	-0.2 (-1.2 to 0.9)	0.0 (-0.6 to 0.5)
Mississippi	1 to 4 years	0.7 (-1.0 to 2.4)	0.6 (-0.3 to 1.5)	0.3 (-0.5 to 1.2)	0.3 (-0.2 to 0.7)
Mississippi	10 to 14 years	-1.1 (-3.0 to 0.8)	-0.6 (-1.6 to 0.4)	-0.6 (-1.5 to 0.4)	-0.3 (-0.8 to 0.2)
Mississippi	15 to 19 years	3.5 (0.9 to 6.1)	0.6 (-0.8 to 2.1)	1.9 (0.5 to 3.4)	0.4 (-0.5 to 1.2)
Mississippi	20 to 24 years	6.0 (2.0 to 9.9)	2.5 (0.3 to 4.7)	3.3 (1.1 to 5.5)	1.4 (0.1 to 2.6)
Mississippi	25 to 29 years	3.7 (-0.6 to 8.1)	0.2 (-2.2 to 2.5)	2.7 (-0.4 to 5.9)	0.1 (-1.6 to 1.8)
Mississippi	30 to 34 years	6.6 (2.5 to 10.6)	0.1 (-2.1 to 2.3)	4.7 (1.8 to 7.5)	0.1 (-1.5 to 1.7)
Mississippi	35 to 39 years	7.0 (3.5 to 10.5)	0.7 (-1.2 to 2.6)	5.1 (2.6 to 7.7)	0.5 (-0.9 to 1.9)
Mississippi	40 to 44 years	6.7 (3.3 to 10.2)	1.5 (-0.4 to 3.4)	5.3 (2.5 to 8.0)	1.1 (-0.3 to 2.6)
Mississippi	45 to 49 years	7.1 (3.7 to 10.6)	2.1 (0.2 to 4.0)	5.0 (2.6 to 7.4)	1.5 (0.1 to 2.8)
Mississippi	5 to 9 years	-0.1 (-1.6 to 1.4)	0.1 (-0.7 to 1.0)	-0.1 (-1.0 to 0.9)	0.1 (-0.4 to 0.6)
Mississippi	50 to 54 years	4.9 (1.8 to 8.0)	2.0 (0.3 to 3.7)	4.3 (1.6 to 7.1)	1.7 (0.2 to 3.2)
Mississippi	55 to 59 years	2.0 (-1.0 to 5.0)	0.6 (-1.0 to 2.3)	1.5 (-0.7 to 3.7)	0.5 (-0.7 to 1.7)
Mississippi	60 to 64 years	2.8 (0.2 to 5.5)	1.4 (0.0 to 2.9)	2.5 (0.2 to 4.9)	1.3 (0.0 to 2.6)
Mississippi	65 to 69 years	0.2 (-0.2 to 0.7)	0.1 (-0.2 to 0.3)	0.2 (-0.2 to 0.6)	0.1 (-0.1 to 0.3)
Mississippi	70 to 74 years	-0.1 (-0.2 to 0.1)	-0.1 (-0.2 to 0.0)	-0.3 (-0.9 to 0.4)	-0.4 (-0.8 to -0.1)

© 2021 Weaver MR et al. *JAMA Network Open*.

Missouri	0 to 11 months	-0.2 (-1.6 to 1.1)	0.0 (-0.8 to 0.7)	-0.1 (-1.1 to 0.8)	0.0 (-0.5 to 0.5)
Missouri	1 to 4 years	0.5 (-0.7 to 1.8)	0.4 (-0.2 to 1.1)	0.4 (-0.6 to 1.4)	0.3 (-0.2 to 0.9)
Missouri	10 to 14 years	-0.9 (-2.4 to 0.6)	-0.5 (-1.3 to 0.3)	-0.7 (-1.8 to 0.5)	-0.4 (-1.0 to 0.3)
Missouri	15 to 19 years	2.4 (0.6 to 4.1)	0.4 (-0.6 to 1.4)	1.6 (0.4 to 2.8)	0.3 (-0.4 to 1.0)
Missouri	20 to 24 years	4.3 (1.4 to 7.1)	1.8 (0.2 to 3.3)	2.5 (0.8 to 4.1)	1.0 (0.1 to 1.9)
Missouri	25 to 29 years	2.9 (-0.4 to 6.3)	0.1 (-1.7 to 2.0)	2.0 (-0.3 to 4.2)	0.1 (-1.1 to 1.3)
Missouri	30 to 34 years	4.4 (1.7 to 7.1)	0.1 (-1.4 to 1.6)	3.3 (1.3 to 5.3)	0.1 (-1.0 to 1.2)
Missouri	35 to 39 years	5.3 (2.7 to 8.0)	0.5 (-0.9 to 2.0)	3.6 (1.8 to 5.4)	0.4 (-0.6 to 1.4)
Missouri	40 to 44 years	4.7 (2.3 to 7.2)	1.0 (-0.3 to 2.4)	3.6 (1.8 to 5.5)	0.8 (-0.2 to 1.8)
Missouri	45 to 49 years	4.9 (2.5 to 7.3)	1.5 (0.1 to 2.8)	3.6 (1.9 to 5.4)	1.1 (0.1 to 2.0)
Missouri	5 to 9 years	-0.1 (-1.2 to 1.0)	0.1 (-0.5 to 0.7)	-0.1 (-1.0 to 0.9)	0.1 (-0.4 to 0.6)
Missouri	50 to 54 years	3.4 (1.2 to 5.5)	1.4 (0.2 to 2.6)	2.8 (1.0 to 4.7)	1.1 (0.1 to 2.1)
Missouri	55 to 59 years	1.6 (-0.8 to 4.0)	0.5 (-0.8 to 1.8)	1.1 (-0.5 to 2.8)	0.3 (-0.6 to 1.3)
Missouri	60 to 64 years	2.4 (0.2 to 4.6)	1.2 (0.0 to 2.4)	1.7 (0.1 to 3.2)	0.8 (0.0 to 1.7)
Missouri	65 to 69 years	0.3 (-0.3 to 0.9)	0.1 (-0.2 to 0.4)	0.2 (-0.2 to 0.5)	0.1 (-0.1 to 0.2)
Missouri	70 to 74 years	-0.3 (-1.1 to 0.5)	-0.5 (-0.9 to -0.1)	-0.2 (-0.5 to 0.2)	-0.2 (-0.4 to 0.0)
Montana	0 to 11 months	-0.3 (-1.9 to 1.4)	0.0 (-0.9 to 0.8)	-0.3 (-2.5 to 1.8)	-0.1 (-1.2 to 1.1)
Montana	1 to 4 years	1.4 (-1.9 to 4.6)	1.1 (-0.6 to 2.9)	0.3 (-0.4 to 0.9)	0.2 (-0.1 to 0.5)
Montana	10 to 14 years	-1.4 (-3.8 to 1.0)	-0.8 (-2.0 to 0.5)	-0.6 (-1.5 to 0.4)	-0.3 (-0.8 to 0.2)
Montana	15 to 19 years	3.3 (0.8 to 5.7)	0.6 (-0.8 to 1.9)	1.6 (0.4 to 2.8)	0.3 (-0.4 to 1.0)
Montana	20 to 24 years	4.7 (1.6 to 7.7)	1.9 (0.2 to 3.6)	2.4 (0.8 to 3.9)	1.0 (0.1 to 1.8)
Montana	25 to 29 years	2.8 (-0.4 to 6.1)	0.1 (-1.6 to 1.9)	1.2 (-0.2 to 2.7)	0.1 (-0.7 to 0.8)
Montana	30 to 34 years	4.8 (1.8 to 7.7)	0.1 (-1.5 to 1.7)	2.4 (0.9 to 3.9)	0.0 (-0.8 to 0.9)
Montana	35 to 39 years	5.8 (2.9 to 8.7)	0.6 (-1.0 to 2.2)	3.5 (1.8 to 5.3)	0.4 (-0.6 to 1.3)
Montana	40 to 44 years	6.8 (3.3 to 10.3)	1.5 (-0.4 to 3.4)	3.4 (1.6 to 5.1)	0.7 (-0.2 to 1.7)
Montana	45 to 49 years	6.7 (3.5 to 10.0)	2.0 (0.2 to 3.8)	4.0 (2.0 to 5.9)	1.2 (0.1 to 2.2)
Montana	5 to 9 years	-0.2 (-3.0 to 2.7)	0.3 (-1.3 to 1.8)	-0.1 (-1.0 to 0.9)	0.1 (-0.4 to 0.6)
Montana	50 to 54 years	5.2 (1.9 to 8.6)	2.1 (0.3 to 3.9)	2.8 (1.0 to 4.6)	1.1 (0.1 to 2.1)
Montana	55 to 59 years	2.0 (-1.0 to 4.9)	0.6 (-1.0 to 2.2)	1.5 (-0.7 to 3.6)	0.5 (-0.7 to 1.6)

© 2021 Weaver MR et al. *JAMA Network Open*.

Montana	60 to 64 years	2.9 (0.2 to 5.5)	1.4 (0.0 to 2.9)	1.5 (0.1 to 3.0)	0.8 (0.0 to 1.6)
Montana	65 to 69 years	0.2 (-0.2 to 0.7)	0.1 (-0.2 to 0.3)	0.2 (-0.1 to 0.5)	0.1 (-0.1 to 0.2)
Montana	70 to 74 years	0.0 (0.0 to 0.0)	0.0 (0.0 to 0.0)	0.0 (-0.1 to 0.0)	0.0 (-0.1 to 0.0)
Nebraska	0 to 11 months	-0.2 (-1.2 to 0.9)	0.0 (-0.6 to 0.5)	-0.3 (-2.4 to 1.8)	-0.1 (-1.2 to 1.1)
Nebraska	1 to 4 years	0.4 (-0.5 to 1.2)	0.3 (-0.2 to 0.7)	0.5 (-0.6 to 1.6)	0.4 (-0.2 to 1.0)
Nebraska	10 to 14 years	-0.8 (-2.2 to 0.5)	-0.4 (-1.2 to 0.3)	-0.7 (-2.0 to 0.5)	-0.4 (-1.1 to 0.3)
Nebraska	15 to 19 years	1.7 (0.4 to 3.0)	0.3 (-0.4 to 1.0)	1.6 (0.4 to 2.9)	0.3 (-0.4 to 1.0)
Nebraska	20 to 24 years	4.5 (1.5 to 7.5)	1.9 (0.2 to 3.5)	2.2 (0.7 to 3.6)	0.9 (0.1 to 1.7)
Nebraska	25 to 29 years	2.6 (-0.4 to 5.6)	0.1 (-1.5 to 1.7)	1.8 (-0.3 to 4.0)	0.1 (-1.1 to 1.2)
Nebraska	30 to 34 years	3.8 (1.5 to 6.2)	0.1 (-1.2 to 1.4)	2.8 (1.1 to 4.6)	0.1 (-0.9 to 1.0)
Nebraska	35 to 39 years	4.0 (2.0 to 5.9)	0.4 (-0.7 to 1.5)	3.4 (1.7 to 5.2)	0.4 (-0.6 to 1.3)
Nebraska	40 to 44 years	4.1 (2.0 to 6.2)	0.9 (-0.3 to 2.1)	3.6 (1.7 to 5.5)	0.8 (-0.2 to 1.8)
Nebraska	45 to 49 years	4.6 (2.3 to 6.8)	1.4 (0.1 to 2.6)	2.8 (1.4 to 4.1)	0.8 (0.1 to 1.6)
Nebraska	5 to 9 years	-0.1 (-1.1 to 1.0)	0.1 (-0.5 to 0.7)	-0.1 (-1.1 to 0.9)	0.1 (-0.4 to 0.6)
Nebraska	50 to 54 years	2.5 (0.9 to 4.2)	1.0 (0.1 to 1.9)	2.5 (0.9 to 4.1)	1.0 (0.1 to 1.9)
Nebraska	55 to 59 years	1.1 (-0.5 to 2.8)	0.4 (-0.6 to 1.3)	1.3 (-0.6 to 3.2)	0.4 (-0.6 to 1.4)
Nebraska	60 to 64 years	2.0 (0.2 to 3.8)	1.0 (0.0 to 2.0)	1.0 (0.1 to 2.0)	0.5 (0.0 to 1.0)
Nebraska	65 to 69 years	0.2 (-0.2 to 0.5)	0.1 (-0.1 to 0.2)	0.4 (-0.3 to 1.0)	0.1 (-0.2 to 0.5)
Nebraska	70 to 74 years	-0.1 (-0.5 to 0.2)	-0.2 (-0.4 to 0.0)	-0.1 (-0.4 to 0.2)	-0.2 (-0.4 to 0.0)
Nevada	0 to 11 months	-0.5 (-3.6 to 2.6)	-0.1 (-1.7 to 1.6)	-0.1 (-0.9 to 0.6)	0.0 (-0.4 to 0.4)
Nevada	1 to 4 years	1.6 (-2.2 to 5.3)	1.3 (-0.7 to 3.3)	0.5 (-0.7 to 1.6)	0.4 (-0.2 to 1.0)
Nevada	10 to 14 years	-2.4 (-6.5 to 1.6)	-1.3 (-3.5 to 0.9)	-0.7 (-1.9 to 0.5)	-0.4 (-1.0 to 0.3)
Nevada	15 to 19 years	5.1 (1.3 to 8.9)	0.9 (-1.2 to 3.1)	2.2 (0.6 to 3.9)	0.4 (-0.5 to 1.3)
Nevada	20 to 24 years	6.4 (2.2 to 10.6)	2.6 (0.3 to 5.0)	2.6 (0.9 to 4.3)	1.1 (0.1 to 2.0)
Nevada	25 to 29 years	4.0 (-0.6 to 8.6)	0.2 (-2.3 to 2.7)	2.2 (-0.3 to 4.7)	0.1 (-1.2 to 1.5)
Nevada	30 to 34 years	6.5 (2.5 to 10.5)	0.1 (-2.1 to 2.3)	3.5 (1.3 to 5.6)	0.1 (-1.1 to 1.2)
Nevada	35 to 39 years	7.4 (3.7 to 11.1)	0.8 (-1.3 to 2.8)	4.5 (2.3 to 6.8)	0.5 (-0.8 to 1.7)
Nevada	40 to 44 years	6.8 (3.3 to 10.3)	1.5 (-0.4 to 3.4)	4.6 (2.2 to 6.9)	1.0 (-0.3 to 2.3)
Nevada	45 to 49 years	7.1 (3.7 to 10.6)	2.1 (0.2 to 4.0)	4.5 (2.3 to 6.7)	1.3 (0.1 to 2.5)

© 2021 Weaver MR et al. *JAMA Network Open*.

Nevada	5 to 9 years	-0.3 (-4.3 to 3.8)	0.4 (-1.8 to 2.6)	-0.1 (-1.2 to 1.1)	0.1 (-0.5 to 0.7)
Nevada	50 to 54 years	5.6 (2.0 to 9.3)	2.3 (0.3 to 4.3)	3.1 (1.1 to 5.2)	1.3 (0.2 to 2.4)
Nevada	55 to 59 years	2.1 (-1.0 to 5.3)	0.7 (-1.1 to 2.4)	1.3 (-0.6 to 3.2)	0.4 (-0.7 to 1.5)
Nevada	60 to 64 years	3.4 (0.3 to 6.5)	1.7 (0.0 to 3.4)	2.2 (0.2 to 4.3)	1.1 (0.0 to 2.3)
Nevada	65 to 69 years	1.1 (-1.0 to 3.2)	0.4 (-0.8 to 1.5)	0.9 (-0.9 to 2.7)	0.3 (-0.6 to 1.2)
Nevada	70 to 74 years	-0.4 (-1.2 to 0.5)	-0.6 (-1.0 to -0.1)	-0.6 (-2.3 to 1.0)	-1.0 (-1.9 to -0.2)
New Hampshire	0 to 11 months	-0.2 (-1.7 to 1.2)	0.0 (-0.8 to 0.7)	0.0 (0.0 to 0.0)	0.0 (0.0 to 0.0)
New Hampshire	1 to 4 years	0.3 (-0.4 to 1.0)	0.2 (-0.1 to 0.6)	0.2 (-0.3 to 0.7)	0.2 (-0.1 to 0.4)
New Hampshire	10 to 14 years	-0.9 (-2.4 to 0.6)	-0.5 (-1.3 to 0.3)	-0.4 (-1.0 to 0.2)	-0.2 (-0.5 to 0.1)
New Hampshire	15 to 19 years	1.8 (0.5 to 3.1)	0.3 (-0.4 to 1.1)	1.1 (0.3 to 2.0)	0.2 (-0.3 to 0.7)
New Hampshire	20 to 24 years	3.6 (1.2 to 6.0)	1.5 (0.2 to 2.8)	1.5 (0.5 to 2.4)	0.6 (0.1 to 1.1)
New Hampshire	25 to 29 years	2.4 (-0.4 to 5.2)	0.1 (-1.4 to 1.6)	1.9 (-0.3 to 4.0)	0.1 (-1.1 to 1.2)
New Hampshire	30 to 34 years	4.0 (1.5 to 6.5)	0.1 (-1.3 to 1.4)	2.0 (0.8 to 3.3)	0.0 (-0.7 to 0.7)
New Hampshire	35 to 39 years	4.2 (2.1 to 6.3)	0.4 (-0.7 to 1.6)	2.9 (1.4 to 4.3)	0.3 (-0.5 to 1.1)
New Hampshire	40 to 44 years	3.8 (1.9 to 5.8)	0.8 (-0.3 to 1.9)	1.8 (0.9 to 2.7)	0.4 (-0.1 to 0.9)
New Hampshire	45 to 49 years	3.9 (2.0 to 5.8)	1.2 (0.1 to 2.2)	2.7 (1.4 to 4.0)	0.8 (0.1 to 1.5)
New Hampshire	5 to 9 years	0.0 (-0.8 to 0.7)	0.1 (-0.3 to 0.5)	0.0 (-0.7 to 0.6)	0.1 (-0.3 to 0.4)
New Hampshire	50 to 54 years	2.9 (1.0 to 4.7)	1.2 (0.1 to 2.2)	1.8 (0.7 to 3.0)	0.7 (0.1 to 1.4)
New Hampshire	55 to 59 years	1.3 (-0.6 to 3.3)	0.4 (-0.7 to 1.5)	0.8 (-0.4 to 2.1)	0.3 (-0.4 to 0.9)
New Hampshire	60 to 64 years	2.4 (0.2 to 4.6)	1.2 (0.0 to 2.4)	1.2 (0.1 to 2.2)	0.6 (0.0 to 1.2)
New Hampshire	65 to 69 years	0.3 (-0.3 to 0.8)	0.1 (-0.2 to 0.4)	0.0 (0.0 to 0.0)	0.0 (0.0 to 0.0)
New Hampshire	70 to 74 years	-0.1 (-0.3 to 0.1)	-0.2 (-0.3 to 0.0)	0.0 (0.0 to 0.0)	0.0 (0.0 to 0.0)
New Jersey	0 to 11 months	-0.3 (-1.9 to 1.4)	0.0 (-0.9 to 0.8)	0.0 (-0.3 to 0.2)	0.0 (-0.2 to 0.1)
New Jersey	1 to 4 years	0.4 (-0.6 to 1.4)	0.4 (-0.2 to 0.9)	0.3 (-0.4 to 1.1)	0.3 (-0.1 to 0.7)
New Jersey	10 to 14 years	-0.8 (-2.3 to 0.6)	-0.4 (-1.2 to 0.3)	-0.4 (-1.0 to 0.2)	-0.2 (-0.5 to 0.1)
New Jersey	15 to 19 years	2.3 (0.6 to 4.0)	0.4 (-0.5 to 1.4)	1.1 (0.3 to 2.0)	0.2 (-0.3 to 0.7)
New Jersey	20 to 24 years	4.5 (1.5 to 7.4)	1.8 (0.2 to 3.5)	1.9 (0.6 to 3.1)	0.8 (0.1 to 1.5)
New Jersey	25 to 29 years	2.9 (-0.4 to 6.3)	0.1 (-1.7 to 1.9)	1.8 (-0.3 to 3.9)	0.1 (-1.0 to 1.2)
New Jersey	30 to 34 years	4.5 (1.7 to 7.2)	0.1 (-1.4 to 1.6)	2.9 (1.1 to 4.6)	0.1 (-0.9 to 1.0)

© 2021 Weaver MR et al. *JAMA Network Open*.

New Jersey	35 to 39 years	4.9 (2.4 to 7.3)	0.5 (-0.9 to 1.8)	3.6 (1.8 to 5.4)	0.4 (-0.6 to 1.4)
New Jersey	40 to 44 years	4.5 (2.2 to 6.9)	1.0 (-0.3 to 2.3)	3.2 (1.5 to 4.8)	0.7 (-0.2 to 1.6)
New Jersey	45 to 49 years	4.3 (2.2 to 6.4)	1.3 (0.1 to 2.4)	3.0 (1.6 to 4.5)	0.9 (0.1 to 1.7)
New Jersey	5 to 9 years	-0.1 (-1.2 to 1.1)	0.1 (-0.5 to 0.7)	0.0 (-0.7 to 0.7)	0.1 (-0.3 to 0.4)
New Jersey	50 to 54 years	3.4 (1.2 to 5.6)	1.4 (0.2 to 2.6)	2.1 (0.8 to 3.4)	0.8 (0.1 to 1.6)
New Jersey	55 to 59 years	1.5 (-0.7 to 3.8)	0.5 (-0.8 to 1.7)	0.8 (-0.4 to 2.0)	0.3 (-0.4 to 0.9)
New Jersey	60 to 64 years	2.6 (0.2 to 5.0)	1.3 (0.0 to 2.6)	1.5 (0.1 to 2.8)	0.7 (0.0 to 1.5)
New Jersey	65 to 69 years	1.2 (-1.1 to 3.5)	0.4 (-0.9 to 1.7)	0.8 (-0.7 to 2.2)	0.3 (-0.5 to 1.0)
New Jersey	70 to 74 years	-0.8 (-3.0 to 1.3)	-1.3 (-2.5 to -0.2)	-0.5 (-1.6 to 0.7)	-0.7 (-1.3 to -0.1)
New Mexico	0 to 11 months	-0.3 (-2.1 to 1.6)	0.0 (-1.0 to 0.9)	-0.2 (-1.6 to 1.2)	0.0 (-0.8 to 0.7)
New Mexico	1 to 4 years	0.7 (-1.0 to 2.4)	0.6 (-0.3 to 1.5)	0.4 (-0.5 to 1.3)	0.3 (-0.2 to 0.8)
New Mexico	10 to 14 years	-1.4 (-3.7 to 0.9)	-0.7 (-2.0 to 0.5)	-1.0 (-2.6 to 0.6)	-0.5 (-1.4 to 0.4)
New Mexico	15 to 19 years	4.2 (1.1 to 7.2)	0.7 (-1.0 to 2.5)	1.4 (0.4 to 2.4)	0.3 (-0.3 to 0.8)
New Mexico	20 to 24 years	5.5 (1.9 to 9.2)	2.3 (0.2 to 4.3)	2.1 (0.7 to 3.5)	0.9 (0.1 to 1.6)
New Mexico	25 to 29 years	4.3 (-0.6 to 9.3)	0.2 (-2.5 to 2.9)	1.6 (-0.2 to 3.4)	0.1 (-0.9 to 1.1)
New Mexico	30 to 34 years	6.8 (2.6 to 11.0)	0.1 (-2.2 to 2.4)	3.4 (1.3 to 5.5)	0.1 (-1.1 to 1.2)
New Mexico	35 to 39 years	7.2 (3.6 to 10.8)	0.7 (-1.3 to 2.7)	4.0 (2.0 to 6.0)	0.4 (-0.7 to 1.5)
New Mexico	40 to 44 years	7.9 (3.8 to 11.9)	1.7 (-0.5 to 4.0)	4.1 (2.0 to 6.2)	0.9 (-0.3 to 2.1)
New Mexico	45 to 49 years	8.4 (4.3 to 12.5)	2.5 (0.2 to 4.8)	4.2 (2.2 to 6.3)	1.3 (0.1 to 2.4)
New Mexico	5 to 9 years	-0.1 (-1.9 to 1.7)	0.2 (-0.8 to 1.2)	-0.1 (-1.0 to 0.8)	0.1 (-0.4 to 0.6)
New Mexico	50 to 54 years	5.4 (1.9 to 8.8)	2.2 (0.3 to 4.1)	2.6 (0.9 to 4.2)	1.0 (0.1 to 1.9)
New Mexico	55 to 59 years	2.3 (-1.1 to 5.6)	0.7 (-1.1 to 2.5)	1.2 (-0.6 to 2.9)	0.4 (-0.6 to 1.3)
New Mexico	60 to 64 years	2.9 (0.2 to 5.7)	1.5 (0.0 to 3.0)	1.7 (0.1 to 3.3)	0.9 (0.0 to 1.7)
New Mexico	65 to 69 years	1.0 (-1.0 to 3.0)	0.3 (-0.7 to 1.4)	0.4 (-0.4 to 1.3)	0.1 (-0.3 to 0.6)
New Mexico	70 to 74 years	-0.4 (-1.4 to 0.6)	-0.6 (-1.2 to -0.1)	-0.2 (-0.8 to 0.3)	-0.3 (-0.6 to -0.1)
New York	0 to 11 months	-0.2 (-1.3 to 1.0)	0.0 (-0.6 to 0.6)	-0.1 (-0.7 to 0.5)	0.0 (-0.3 to 0.3)
New York	1 to 4 years	0.4 (-0.6 to 1.5)	0.4 (-0.2 to 0.9)	0.2 (-0.2 to 0.6)	0.2 (-0.1 to 0.4)
New York	10 to 14 years	-0.6 (-1.7 to 0.4)	-0.3 (-0.9 to 0.2)	-0.3 (-0.9 to 0.2)	-0.2 (-0.5 to 0.1)
New York	15 to 19 years	1.8 (0.4 to 3.1)	0.3 (-0.4 to 1.1)	0.8 (0.2 to 1.4)	0.1 (-0.2 to 0.5)

© 2021 Weaver MR et al. *JAMA Network Open*.

New York	20 to 24 years	3.7 (1.3 to 6.2)	1.5 (0.2 to 2.9)	1.2 (0.4 to 2.0)	0.5 (0.1 to 0.9)
New York	25 to 29 years	2.7 (-0.4 to 5.8)	0.1 (-1.6 to 1.8)	1.2 (-0.2 to 2.7)	0.1 (-0.7 to 0.8)
New York	30 to 34 years	4.3 (1.7 to 7.0)	0.1 (-1.4 to 1.5)	2.3 (0.9 to 3.8)	0.0 (-0.8 to 0.8)
New York	35 to 39 years	4.7 (2.3 to 7.0)	0.5 (-0.8 to 1.8)	2.8 (1.4 to 4.2)	0.3 (-0.5 to 1.1)
New York	40 to 44 years	4.3 (2.1 to 6.6)	0.9 (-0.3 to 2.2)	2.9 (1.4 to 4.3)	0.6 (-0.2 to 1.4)
New York	45 to 49 years	3.8 (2.0 to 5.7)	1.1 (0.1 to 2.2)	2.5 (1.3 to 3.7)	0.7 (0.1 to 1.4)
New York	5 to 9 years	-0.1 (-1.0 to 0.9)	0.1 (-0.4 to 0.6)	0.0 (-0.6 to 0.5)	0.0 (-0.2 to 0.3)
New York	50 to 54 years	2.9 (1.1 to 4.8)	1.2 (0.2 to 2.2)	1.6 (0.6 to 2.7)	0.7 (0.1 to 1.2)
New York	55 to 59 years	1.2 (-0.6 to 3.0)	0.4 (-0.6 to 1.3)	0.6 (-0.3 to 1.5)	0.2 (-0.3 to 0.7)
New York	60 to 64 years	1.9 (0.1 to 3.6)	0.9 (0.0 to 1.9)	1.1 (0.1 to 2.1)	0.5 (0.0 to 1.1)
New York	65 to 69 years	0.6 (-0.5 to 1.7)	0.2 (-0.4 to 0.8)	0.5 (-0.5 to 1.5)	0.2 (-0.4 to 0.7)
New York	70 to 74 years	-0.4 (-1.3 to 0.6)	-0.6 (-1.1 to -0.1)	-0.4 (-1.3 to 0.6)	-0.6 (-1.1 to -0.1)
North Carolina	0 to 11 months	-0.2 (-1.6 to 1.2)	0.0 (-0.8 to 0.7)	-0.2 (-1.2 to 0.9)	0.0 (-0.6 to 0.5)
North Carolina	1 to 4 years	0.6 (-0.8 to 2.0)	0.5 (-0.3 to 1.2)	0.3 (-0.4 to 1.0)	0.3 (-0.1 to 0.6)
North Carolina	10 to 14 years	-1.2 (-3.3 to 0.8)	-0.6 (-1.8 to 0.5)	-0.6 (-1.5 to 0.4)	-0.3 (-0.8 to 0.2)
North Carolina	15 to 19 years	3.1 (0.8 to 5.5)	0.6 (-0.7 to 1.9)	1.5 (0.4 to 2.7)	0.3 (-0.4 to 0.9)
North Carolina	20 to 24 years	4.9 (1.7 to 8.2)	2.0 (0.2 to 3.8)	2.4 (0.8 to 4.0)	1.0 (0.1 to 1.9)
North Carolina	25 to 29 years	3.3 (-0.5 to 7.1)	0.2 (-1.9 to 2.2)	2.2 (-0.3 to 4.8)	0.1 (-1.3 to 1.5)
North Carolina	30 to 34 years	5.9 (2.3 to 9.6)	0.1 (-1.9 to 2.1)	3.9 (1.5 to 6.3)	0.1 (-1.2 to 1.4)
North Carolina	35 to 39 years	6.4 (3.2 to 9.6)	0.7 (-1.1 to 2.4)	4.6 (2.3 to 6.9)	0.5 (-0.8 to 1.7)
North Carolina	40 to 44 years	6.1 (3.0 to 9.2)	1.3 (-0.4 to 3.1)	4.6 (2.2 to 7.0)	1.0 (-0.3 to 2.3)
North Carolina	45 to 49 years	6.1 (3.1 to 9.1)	1.8 (0.2 to 3.5)	4.2 (2.2 to 6.3)	1.3 (0.1 to 2.4)
North Carolina	5 to 9 years	-0.1 (-1.6 to 1.4)	0.1 (-0.7 to 1.0)	-0.1 (-1.0 to 0.9)	0.1 (-0.4 to 0.6)
North Carolina	50 to 54 years	4.5 (1.6 to 7.4)	1.8 (0.2 to 3.4)	3.4 (1.2 to 5.6)	1.4 (0.2 to 2.6)
North Carolina	55 to 59 years	1.8 (-0.9 to 4.4)	0.6 (-0.9 to 2.0)	1.2 (-0.6 to 3.0)	0.4 (-0.6 to 1.4)
North Carolina	60 to 64 years	2.7 (0.2 to 5.3)	1.4 (0.0 to 2.8)	1.7 (0.1 to 3.3)	0.9 (0.0 to 1.7)
North Carolina	65 to 69 years	0.4 (-0.3 to 1.0)	0.1 (-0.3 to 0.5)	0.3 (-0.3 to 0.9)	0.1 (-0.2 to 0.4)
North Carolina	70 to 74 years	-0.2 (-0.7 to 0.3)	-0.3 (-0.6 to -0.1)	-0.2 (-0.7 to 0.3)	-0.3 (-0.6 to -0.1)
North Dakota	0 to 11 months	-0.1 (-0.8 to 0.6)	0.0 (-0.4 to 0.4)	-0.3 (-1.9 to 1.4)	0.0 (-0.9 to 0.8)

© 2021 Weaver MR et al. *JAMA Network Open*.

North Dakota	1 to 4 years	0.4 (-0.5 to 1.2)	0.3 (-0.2 to 0.8)	1.1 (-1.5 to 3.7)	0.9 (-0.5 to 2.3)
North Dakota	10 to 14 years	-1.2 (-3.1 to 0.8)	-0.6 (-1.6 to 0.4)	-1.1 (-3.0 to 0.7)	-0.6 (-1.6 to 0.4)
North Dakota	15 to 19 years	2.0 (0.5 to 3.5)	0.4 (-0.5 to 1.2)	1.7 (0.4 to 3.0)	0.3 (-0.4 to 1.0)
North Dakota	20 to 24 years	2.7 (0.9 to 4.5)	1.1 (0.1 to 2.1)	1.6 (0.6 to 2.7)	0.7 (0.1 to 1.3)
North Dakota	25 to 29 years	1.8 (-0.3 to 3.9)	0.1 (-1.0 to 1.2)	1.1 (-0.2 to 2.3)	0.1 (-0.6 to 0.7)
North Dakota	30 to 34 years	3.4 (1.3 to 5.5)	0.1 (-1.1 to 1.2)	2.1 (0.8 to 3.3)	0.0 (-0.7 to 0.7)
North Dakota	35 to 39 years	3.1 (1.5 to 4.6)	0.3 (-0.5 to 1.2)	3.6 (1.8 to 5.5)	0.4 (-0.6 to 1.4)
North Dakota	40 to 44 years	3.3 (1.6 to 4.9)	0.7 (-0.2 to 1.6)	3.1 (1.5 to 4.7)	0.7 (-0.2 to 1.5)
North Dakota	45 to 49 years	3.7 (1.9 to 5.6)	1.1 (0.1 to 2.1)	1.7 (0.9 to 2.6)	0.5 (0.1 to 1.0)
North Dakota	5 to 9 years	-0.1 (-1.3 to 1.1)	0.1 (-0.5 to 0.7)	-0.2 (-2.8 to 2.5)	0.2 (-1.2 to 1.7)
North Dakota	50 to 54 years	3.3 (1.2 to 5.4)	1.3 (0.2 to 2.5)	1.8 (0.7 to 3.0)	0.7 (0.1 to 1.4)
North Dakota	55 to 59 years	1.2 (-0.6 to 2.9)	0.4 (-0.6 to 1.3)	0.6 (-0.3 to 1.4)	0.2 (-0.3 to 0.7)
North Dakota	60 to 64 years	1.6 (0.1 to 3.1)	0.8 (0.0 to 1.6)	1.2 (0.1 to 2.3)	0.6 (0.0 to 1.2)
North Dakota	65 to 69 years	0.1 (-0.1 to 0.3)	0.0 (-0.1 to 0.1)	0.2 (-0.2 to 0.5)	0.1 (-0.1 to 0.2)
North Dakota	70 to 74 years	0.0 (0.0 to 0.0)	0.0 (0.0 to 0.0)	0.0 (-0.1 to 0.0)	0.0 (-0.1 to 0.0)
Ohio	0 to 11 months	-0.2 (-1.7 to 1.2)	0.0 (-0.8 to 0.7)	-0.2 (-1.2 to 0.9)	0.0 (-0.6 to 0.5)
Ohio	1 to 4 years	0.5 (-0.6 to 1.6)	0.4 (-0.2 to 1.0)	0.3 (-0.5 to 1.1)	0.3 (-0.1 to 0.7)
Ohio	10 to 14 years	-0.8 (-2.1 to 0.5)	-0.4 (-1.1 to 0.3)	-0.4 (-1.1 to 0.3)	-0.2 (-0.6 to 0.2)
Ohio	15 to 19 years	2.2 (0.6 to 3.9)	0.4 (-0.5 to 1.3)	0.9 (0.2 to 1.6)	0.2 (-0.2 to 0.6)
Ohio	20 to 24 years	4.2 (1.4 to 6.9)	1.7 (0.2 to 3.2)	1.5 (0.5 to 2.5)	0.6 (0.1 to 1.2)
Ohio	25 to 29 years	2.6 (-0.4 to 5.5)	0.1 (-1.5 to 1.7)	1.3 (-0.2 to 2.8)	0.1 (-0.7 to 0.9)
Ohio	30 to 34 years	4.0 (1.5 to 6.4)	0.1 (-1.3 to 1.4)	2.0 (0.8 to 3.3)	0.0 (-0.6 to 0.7)
Ohio	35 to 39 years	4.4 (2.2 to 6.6)	0.4 (-0.8 to 1.7)	2.4 (1.2 to 3.6)	0.2 (-0.4 to 0.9)
Ohio	40 to 44 years	4.5 (2.2 to 6.8)	1.0 (-0.3 to 2.3)	2.0 (1.0 to 3.1)	0.4 (-0.1 to 1.0)
Ohio	45 to 49 years	4.7 (2.4 to 7.0)	1.4 (0.1 to 2.6)	2.2 (1.1 to 3.3)	0.7 (0.1 to 1.3)
Ohio	5 to 9 years	-0.1 (-1.4 to 1.2)	0.1 (-0.6 to 0.8)	0.0 (-0.8 to 0.7)	0.1 (-0.3 to 0.4)
Ohio	50 to 54 years	3.5 (1.3 to 5.7)	1.4 (0.2 to 2.6)	1.6 (0.6 to 2.7)	0.7 (0.1 to 1.2)
Ohio	55 to 59 years	1.5 (-0.7 to 3.7)	0.5 (-0.8 to 1.7)	0.7 (-0.3 to 1.8)	0.2 (-0.4 to 0.8)
Ohio	60 to 64 years	2.2 (0.2 to 4.2)	1.1 (0.0 to 2.2)	1.2 (0.1 to 2.3)	0.6 (0.0 to 1.2)

© 2021 Weaver MR et al. *JAMA Network Open*.

Ohio	65 to 69 years	0.3 (-0.2 to 0.7)	0.1 (-0.2 to 0.3)	0.2 (-0.2 to 0.7)	0.1 (-0.2 to 0.3)
Ohio	70 to 74 years	-0.2 (-0.7 to 0.3)	-0.3 (-0.6 to -0.1)	-0.2 (-0.5 to 0.2)	-0.2 (-0.5 to 0.0)
Oklahoma	0 to 11 months	-0.2 (-1.5 to 1.1)	0.0 (-0.7 to 0.6)	-0.4 (-2.6 to 1.9)	-0.1 (-1.2 to 1.1)
Oklahoma	1 to 4 years	0.7 (-1.0 to 2.4)	0.6 (-0.3 to 1.5)	0.6 (-0.8 to 2.0)	0.5 (-0.3 to 1.3)
Oklahoma	10 to 14 years	-1.5 (-3.9 to 1.0)	-0.8 (-2.1 to 0.6)	-0.9 (-2.5 to 0.6)	-0.5 (-1.3 to 0.4)
Oklahoma	15 to 19 years	3.8 (1.0 to 6.5)	0.7 (-0.9 to 2.2)	2.6 (0.7 to 4.5)	0.5 (-0.6 to 1.6)
Oklahoma	20 to 24 years	5.8 (2.0 to 9.6)	2.4 (0.3 to 4.5)	3.5 (1.2 to 5.8)	1.4 (0.2 to 2.7)
Oklahoma	25 to 29 years	3.8 (-0.6 to 8.1)	0.2 (-2.2 to 2.5)	2.7 (-0.4 to 5.9)	0.1 (-1.6 to 1.8)
Oklahoma	30 to 34 years	7.0 (2.7 to 11.3)	0.1 (-2.2 to 2.5)	4.9 (1.9 to 7.8)	0.1 (-1.6 to 1.7)
Oklahoma	35 to 39 years	7.3 (3.7 to 11.0)	0.7 (-1.3 to 2.8)	5.3 (2.7 to 8.0)	0.5 (-0.9 to 2.0)
Oklahoma	40 to 44 years	7.1 (3.4 to 10.7)	1.5 (-0.5 to 3.5)	5.9 (2.9 to 9.0)	1.3 (-0.4 to 3.0)
Oklahoma	45 to 49 years	7.3 (3.8 to 10.9)	2.2 (0.2 to 4.2)	6.1 (3.1 to 9.1)	1.8 (0.2 to 3.5)
Oklahoma	5 to 9 years	-0.2 (-2.5 to 2.2)	0.2 (-1.0 to 1.5)	-0.1 (-1.3 to 1.2)	0.1 (-0.6 to 0.8)
Oklahoma	50 to 54 years	4.8 (1.7 to 7.9)	1.9 (0.2 to 3.6)	4.4 (1.6 to 7.2)	1.8 (0.2 to 3.3)
Oklahoma	55 to 59 years	2.2 (-1.0 to 5.3)	0.7 (-1.1 to 2.4)	1.8 (-0.9 to 4.4)	0.5 (-0.9 to 2.0)
Oklahoma	60 to 64 years	2.8 (0.2 to 5.5)	1.4 (0.0 to 2.9)	2.6 (0.2 to 5.0)	1.3 (0.0 to 2.6)
Oklahoma	65 to 69 years	0.5 (-0.4 to 1.4)	0.2 (-0.3 to 0.6)	0.3 (-0.3 to 1.0)	0.1 (-0.2 to 0.5)
Oklahoma	70 to 74 years	-0.2 (-0.8 to 0.3)	-0.4 (-0.7 to -0.1)	-0.2 (-0.8 to 0.3)	-0.3 (-0.6 to -0.1)
Oregon	0 to 11 months	-0.2 (-1.8 to 1.3)	0.0 (-0.8 to 0.8)	-0.1 (-0.4 to 0.3)	0.0 (-0.2 to 0.2)
Oregon	1 to 4 years	0.6 (-0.8 to 2.1)	0.5 (-0.3 to 1.3)	0.2 (-0.2 to 0.5)	0.1 (-0.1 to 0.3)
Oregon	10 to 14 years	-1.3 (-3.6 to 0.9)	-0.7 (-1.9 to 0.5)	-0.5 (-1.3 to 0.3)	-0.3 (-0.7 to 0.2)
Oregon	15 to 19 years	3.0 (0.8 to 5.3)	0.5 (-0.7 to 1.8)	0.8 (0.2 to 1.5)	0.2 (-0.2 to 0.5)
Oregon	20 to 24 years	5.5 (1.9 to 9.1)	2.3 (0.2 to 4.3)	1.5 (0.5 to 2.4)	0.6 (0.1 to 1.1)
Oregon	25 to 29 years	3.7 (-0.5 to 7.9)	0.2 (-2.1 to 2.4)	1.2 (-0.2 to 2.6)	0.1 (-0.7 to 0.8)
Oregon	30 to 34 years	5.4 (2.1 to 8.7)	0.1 (-1.7 to 1.9)	2.1 (0.8 to 3.5)	0.0 (-0.7 to 0.8)
Oregon	35 to 39 years	6.1 (3.0 to 9.1)	0.6 (-1.1 to 2.3)	2.6 (1.3 to 3.9)	0.3 (-0.5 to 1.0)
Oregon	40 to 44 years	5.8 (2.8 to 8.8)	1.3 (-0.4 to 2.9)	2.6 (1.3 to 3.9)	0.6 (-0.2 to 1.3)
Oregon	45 to 49 years	6.5 (3.3 to 9.7)	1.9 (0.2 to 3.7)	2.8 (1.4 to 4.2)	0.8 (0.1 to 1.6)
Oregon	5 to 9 years	-0.1 (-1.8 to 1.6)	0.2 (-0.8 to 1.1)	-0.1 (-0.8 to 0.7)	0.1 (-0.3 to 0.5)

© 2021 Weaver MR et al. *JAMA Network Open*.

Oregon	50 to 54 years	4.9 (1.8 to 8.0)	2.0 (0.3 to 3.7)	2.2 (0.8 to 3.6)	0.9 (0.1 to 1.6)
Oregon	55 to 59 years	1.9 (-0.9 to 4.8)	0.6 (-1.0 to 2.2)	0.8 (-0.4 to 2.1)	0.3 (-0.4 to 1.0)
Oregon	60 to 64 years	3.0 (0.2 to 5.8)	1.5 (0.0 to 3.0)	1.4 (0.1 to 2.6)	0.7 (0.0 to 1.4)
Oregon	65 to 69 years	0.6 (-0.6 to 1.8)	0.2 (-0.4 to 0.9)	0.4 (-0.4 to 1.3)	0.1 (-0.3 to 0.6)
Oregon	70 to 74 years	-0.2 (-0.8 to 0.4)	-0.4 (-0.7 to -0.1)	-0.4 (-1.5 to 0.7)	-0.7 (-1.3 to -0.1)
Pennsylvania	0 to 11 months	-0.2 (-1.8 to 1.3)	0.0 (-0.9 to 0.8)	-0.2 (-1.7 to 1.2)	0.0 (-0.8 to 0.7)
Pennsylvania	1 to 4 years	0.5 (-0.7 to 1.7)	0.4 (-0.2 to 1.0)	0.5 (-0.6 to 1.6)	0.4 (-0.2 to 1.0)
Pennsylvania	10 to 14 years	-0.7 (-1.8 to 0.4)	-0.3 (-0.9 to 0.2)	-0.6 (-1.5 to 0.4)	-0.3 (-0.8 to 0.2)
Pennsylvania	15 to 19 years	1.6 (0.4 to 2.8)	0.3 (-0.4 to 1.0)	1.0 (0.3 to 1.8)	0.2 (-0.2 to 0.6)
Pennsylvania	20 to 24 years	3.6 (1.2 to 6.0)	1.5 (0.2 to 2.8)	1.5 (0.5 to 2.5)	0.6 (0.1 to 1.2)
Pennsylvania	25 to 29 years	2.5 (-0.4 to 5.3)	0.1 (-1.4 to 1.7)	1.4 (-0.2 to 3.0)	0.1 (-0.8 to 0.9)
Pennsylvania	30 to 34 years	3.7 (1.4 to 6.0)	0.1 (-1.2 to 1.3)	2.1 (0.8 to 3.4)	0.0 (-0.7 to 0.8)
Pennsylvania	35 to 39 years	3.8 (1.9 to 5.7)	0.4 (-0.7 to 1.4)	2.6 (1.3 to 3.8)	0.3 (-0.4 to 1.0)
Pennsylvania	40 to 44 years	3.6 (1.8 to 5.5)	0.8 (-0.2 to 1.8)	2.2 (1.1 to 3.4)	0.5 (-0.1 to 1.1)
Pennsylvania	45 to 49 years	3.8 (1.9 to 5.6)	1.1 (0.1 to 2.1)	2.1 (1.1 to 3.1)	0.6 (0.1 to 1.2)
Pennsylvania	5 to 9 years	-0.1 (-1.3 to 1.1)	0.1 (-0.5 to 0.7)	-0.1 (-1.1 to 1.0)	0.1 (-0.5 to 0.7)
Pennsylvania	50 to 54 years	2.7 (1.0 to 4.4)	1.1 (0.1 to 2.0)	1.6 (0.6 to 2.6)	0.6 (0.1 to 1.2)
Pennsylvania	55 to 59 years	1.1 (-0.5 to 2.6)	0.3 (-0.5 to 1.2)	0.7 (-0.3 to 1.8)	0.2 (-0.4 to 0.8)
Pennsylvania	60 to 64 years	1.7 (0.1 to 3.2)	0.8 (0.0 to 1.7)	1.0 (0.1 to 2.0)	0.5 (0.0 to 1.0)
Pennsylvania	65 to 69 years	0.3 (-0.2 to 0.7)	0.1 (-0.2 to 0.3)	0.3 (-0.3 to 1.0)	0.1 (-0.2 to 0.5)
Pennsylvania	70 to 74 years	-0.2 (-0.6 to 0.2)	-0.3 (-0.5 to 0.0)	-0.3 (-0.9 to 0.4)	-0.4 (-0.7 to -0.1)
Rhode Island	0 to 11 months	-0.2 (-1.7 to 1.2)	0.0 (-0.8 to 0.7)	-0.2 (-1.4 to 1.0)	0.0 (-0.7 to 0.6)
Rhode Island	1 to 4 years	0.6 (-0.8 to 2.0)	0.5 (-0.3 to 1.2)	0.2 (-0.3 to 0.7)	0.2 (-0.1 to 0.4)
Rhode Island	10 to 14 years	-0.6 (-1.7 to 0.4)	-0.3 (-0.9 to 0.2)	-0.2 (-0.6 to 0.1)	-0.1 (-0.3 to 0.1)
Rhode Island	15 to 19 years	2.0 (0.5 to 3.5)	0.4 (-0.5 to 1.2)	0.5 (0.1 to 0.8)	0.1 (-0.1 to 0.3)
Rhode Island	20 to 24 years	4.4 (1.5 to 7.3)	1.8 (0.2 to 3.4)	1.1 (0.4 to 1.9)	0.5 (0.0 to 0.9)
Rhode Island	25 to 29 years	2.6 (-0.4 to 5.5)	0.1 (-1.5 to 1.7)	1.1 (-0.2 to 2.5)	0.1 (-0.7 to 0.8)
Rhode Island	30 to 34 years	4.1 (1.6 to 6.6)	0.1 (-1.3 to 1.5)	1.8 (0.7 to 2.8)	0.0 (-0.6 to 0.6)
Rhode Island	35 to 39 years	3.8 (1.9 to 5.7)	0.4 (-0.7 to 1.4)	2.0 (1.0 to 3.1)	0.2 (-0.4 to 0.8)

© 2021 Weaver MR et al. *JAMA Network Open*.

Rhode Island	40 to 44 years	3.8 (1.8 to 5.7)	0.8 (-0.3 to 1.9)	1.7 (0.8 to 2.5)	0.4 (-0.1 to 0.8)
Rhode Island	45 to 49 years	4.4 (2.2 to 6.5)	1.3 (0.1 to 2.5)	1.5 (0.8 to 2.3)	0.5 (0.0 to 0.9)
Rhode Island	5 to 9 years	0.0 (-0.7 to 0.6)	0.1 (-0.3 to 0.4)	0.0 (-0.5 to 0.4)	0.0 (-0.2 to 0.3)
Rhode Island	50 to 54 years	3.2 (1.2 to 5.3)	1.3 (0.2 to 2.4)	1.0 (0.3 to 1.6)	0.4 (0.0 to 0.7)
Rhode Island	55 to 59 years	1.2 (-0.6 to 3.1)	0.4 (-0.6 to 1.4)	0.2 (-0.1 to 0.6)	0.1 (-0.1 to 0.2)
Rhode Island	60 to 64 years	2.3 (0.2 to 4.3)	1.1 (0.0 to 2.3)	0.5 (0.0 to 1.0)	0.3 (0.0 to 0.5)
Rhode Island	65 to 69 years	0.9 (-0.8 to 2.6)	0.3 (-0.6 to 1.2)	0.3 (-0.3 to 0.8)	0.1 (-0.2 to 0.4)
Rhode Island	70 to 74 years	-0.5 (-1.6 to 0.7)	-0.7 (-1.3 to -0.1)	-0.1 (-0.5 to 0.2)	-0.2 (-0.4 to 0.0)
South Carolina	0 to 11 months	-0.3 (-2.3 to 1.7)	-0.1 (-1.1 to 1.0)	-0.1 (-0.8 to 0.6)	0.0 (-0.4 to 0.3)
South Carolina	1 to 4 years	0.7 (-1.0 to 2.5)	0.6 (-0.3 to 1.6)	0.4 (-0.5 to 1.2)	0.3 (-0.2 to 0.8)
South Carolina	10 to 14 years	-1.4 (-3.8 to 0.9)	-0.7 (-2.0 to 0.5)	-0.5 (-1.3 to 0.3)	-0.3 (-0.7 to 0.2)
South Carolina	15 to 19 years	3.2 (0.8 to 5.6)	0.6 (-0.8 to 1.9)	1.4 (0.4 to 2.4)	0.3 (-0.3 to 0.8)
South Carolina	20 to 24 years	5.1 (1.7 to 8.4)	2.1 (0.2 to 3.9)	2.6 (0.9 to 4.4)	1.1 (0.1 to 2.1)
South Carolina	25 to 29 years	3.5 (-0.5 to 7.6)	0.2 (-2.0 to 2.4)	2.0 (-0.3 to 4.4)	0.1 (-1.2 to 1.4)
South Carolina	30 to 34 years	5.6 (2.2 to 9.1)	0.1 (-1.8 to 2.0)	3.7 (1.4 to 5.9)	0.1 (-1.2 to 1.3)
South Carolina	35 to 39 years	6.2 (3.1 to 9.2)	0.6 (-1.1 to 2.3)	4.5 (2.2 to 6.7)	0.5 (-0.8 to 1.7)
South Carolina	40 to 44 years	6.6 (3.2 to 10.0)	1.4 (-0.4 to 3.3)	4.2 (2.1 to 6.4)	0.9 (-0.3 to 2.1)
South Carolina	45 to 49 years	6.5 (3.4 to 9.7)	1.9 (0.2 to 3.7)	4.3 (2.2 to 6.4)	1.3 (0.1 to 2.4)
South Carolina	5 to 9 years	-0.1 (-2.2 to 1.9)	0.2 (-0.9 to 1.3)	0.0 (-0.8 to 0.7)	0.1 (-0.3 to 0.5)
South Carolina	50 to 54 years	5.0 (1.8 to 8.2)	2.0 (0.3 to 3.8)	3.5 (1.3 to 5.7)	1.4 (0.2 to 2.6)
South Carolina	55 to 59 years	1.9 (-0.9 to 4.7)	0.6 (-1.0 to 2.1)	1.4 (-0.7 to 3.4)	0.4 (-0.7 to 1.5)
South Carolina	60 to 64 years	3.2 (0.2 to 6.2)	1.6 (0.0 to 3.3)	2.1 (0.2 to 4.0)	1.0 (0.0 to 2.1)
South Carolina	65 to 69 years	0.4 (-0.4 to 1.1)	0.1 (-0.3 to 0.5)	0.3 (-0.3 to 1.0)	0.1 (-0.2 to 0.5)
South Carolina	70 to 74 years	-0.3 (-1.1 to 0.5)	-0.5 (-1.0 to -0.1)	-0.1 (-0.2 to 0.1)	-0.1 (-0.2 to 0.0)
South Dakota	0 to 11 months	-0.2 (-1.6 to 1.2)	0.0 (-0.8 to 0.7)	-0.2 (-1.7 to 1.2)	0.0 (-0.8 to 0.7)
South Dakota	1 to 4 years	0.5 (-0.7 to 1.7)	0.4 (-0.2 to 1.1)	0.3 (-0.4 to 1.0)	0.2 (-0.1 to 0.6)
South Dakota	10 to 14 years	-0.9 (-2.4 to 0.6)	-0.5 (-1.3 to 0.3)	-0.5 (-1.4 to 0.3)	-0.3 (-0.7 to 0.2)
South Dakota	15 to 19 years	2.2 (0.6 to 3.9)	0.4 (-0.5 to 1.3)	1.9 (0.5 to 3.2)	0.3 (-0.4 to 1.1)
South Dakota	20 to 24 years	3.6 (1.2 to 6.0)	1.5 (0.2 to 2.8)	2.2 (0.7 to 3.6)	0.9 (0.1 to 1.7)

© 2021 Weaver MR et al. *JAMA Network Open*.

South Dakota	25 to 29 years	2.4 (-0.4 to 5.2)	0.1 (-1.4 to 1.6)	2.1 (-0.3 to 4.4)	0.1 (-1.2 to 1.4)
South Dakota	30 to 34 years	4.1 (1.6 to 6.6)	0.1 (-1.3 to 1.4)	3.2 (1.2 to 5.2)	0.1 (-1.0 to 1.1)
South Dakota	35 to 39 years	5.1 (2.6 to 7.7)	0.5 (-0.9 to 1.9)	2.8 (1.4 to 4.2)	0.3 (-0.5 to 1.1)
South Dakota	40 to 44 years	4.0 (2.0 to 6.1)	0.9 (-0.3 to 2.0)	3.6 (1.8 to 5.5)	0.8 (-0.2 to 1.8)
South Dakota	45 to 49 years	4.0 (2.0 to 5.9)	1.2 (0.1 to 2.2)	3.4 (1.8 to 5.1)	1.0 (0.1 to 1.9)
South Dakota	5 to 9 years	-0.1 (-1.9 to 1.6)	0.2 (-0.8 to 1.1)	-0.1 (-0.9 to 0.8)	0.1 (-0.4 to 0.6)
South Dakota	50 to 54 years	3.2 (1.2 to 5.2)	1.3 (0.2 to 2.4)	1.9 (0.7 to 3.1)	0.8 (0.1 to 1.4)
South Dakota	55 to 59 years	1.5 (-0.7 to 3.7)	0.5 (-0.8 to 1.7)	1.3 (-0.6 to 3.1)	0.4 (-0.6 to 1.4)
South Dakota	60 to 64 years	1.9 (0.1 to 3.7)	1.0 (0.0 to 2.0)	1.7 (0.1 to 3.3)	0.9 (0.0 to 1.7)
South Dakota	65 to 69 years	0.2 (-0.2 to 0.6)	0.1 (-0.2 to 0.3)	0.2 (-0.1 to 0.5)	0.1 (-0.1 to 0.2)
South Dakota	70 to 74 years	-0.4 (-1.5 to 0.6)	-0.7 (-1.2 to -0.1)	-0.1 (-0.2 to 0.1)	-0.1 (-0.2 to 0.0)
Tennessee	0 to 11 months	-0.2 (-1.2 to 0.9)	0.0 (-0.6 to 0.5)	-0.2 (-1.1 to 0.8)	0.0 (-0.5 to 0.5)
Tennessee	1 to 4 years	0.4 (-0.6 to 1.3)	0.3 (-0.2 to 0.8)	0.3 (-0.4 to 0.9)	0.2 (-0.1 to 0.5)
Tennessee	10 to 14 years	-0.7 (-1.9 to 0.5)	-0.4 (-1.0 to 0.3)	-0.6 (-1.5 to 0.4)	-0.3 (-0.8 to 0.2)
Tennessee	15 to 19 years	2.2 (0.6 to 3.9)	0.4 (-0.5 to 1.3)	1.1 (0.3 to 2.0)	0.2 (-0.3 to 0.7)
Tennessee	20 to 24 years	4.6 (1.6 to 7.7)	1.9 (0.2 to 3.6)	2.0 (0.7 to 3.2)	0.8 (0.1 to 1.5)
Tennessee	25 to 29 years	3.2 (-0.5 to 6.9)	0.2 (-1.8 to 2.1)	2.0 (-0.3 to 4.4)	0.1 (-1.2 to 1.4)
Tennessee	30 to 34 years	5.0 (1.9 to 8.0)	0.1 (-1.6 to 1.8)	3.3 (1.3 to 5.4)	0.1 (-1.1 to 1.2)
Tennessee	35 to 39 years	5.3 (2.7 to 8.0)	0.5 (-0.9 to 2.0)	4.4 (2.2 to 6.6)	0.5 (-0.8 to 1.7)
Tennessee	40 to 44 years	5.6 (2.7 to 8.5)	1.2 (-0.4 to 2.8)	4.1 (2.0 to 6.2)	0.9 (-0.3 to 2.1)
Tennessee	45 to 49 years	6.0 (3.1 to 8.9)	1.8 (0.2 to 3.4)	4.4 (2.2 to 6.5)	1.3 (0.1 to 2.5)
Tennessee	5 to 9 years	-0.1 (-1.0 to 0.9)	0.1 (-0.4 to 0.6)	0.0 (-0.7 to 0.6)	0.1 (-0.3 to 0.4)
Tennessee	50 to 54 years	4.2 (1.5 to 6.8)	1.7 (0.2 to 3.1)	2.8 (1.0 to 4.6)	1.1 (0.1 to 2.1)
Tennessee	55 to 59 years	1.8 (-0.9 to 4.5)	0.6 (-0.9 to 2.0)	1.1 (-0.6 to 2.8)	0.4 (-0.6 to 1.3)
Tennessee	60 to 64 years	2.4 (0.2 to 4.7)	1.2 (0.0 to 2.5)	1.8 (0.1 to 3.4)	0.9 (0.0 to 1.8)
Tennessee	65 to 69 years	0.5 (-0.5 to 1.5)	0.2 (-0.4 to 0.7)	0.2 (-0.2 to 0.6)	0.1 (-0.1 to 0.3)
Tennessee	70 to 74 years	-0.3 (-1.1 to 0.5)	-0.5 (-0.9 to -0.1)	-0.2 (-0.7 to 0.3)	-0.3 (-0.6 to -0.1)
Texas	0 to 11 months	-0.4 (-2.6 to 1.9)	-0.1 (-1.2 to 1.1)	-0.2 (-1.7 to 1.2)	0.0 (-0.8 to 0.7)
Texas	1 to 4 years	1.1 (-1.5 to 3.7)	0.9 (-0.5 to 2.3)	0.7 (-1.0 to 2.5)	0.6 (-0.3 to 1.6)

© 2021 Weaver MR et al. *JAMA Network Open*.

Texas	10 to 14 years	-2.2 (-5.9 to 1.5)	-1.2 (-3.1 to 0.8)	-1.3 (-3.4 to 0.8)	-0.7 (-1.8 to 0.5)
Texas	15 to 19 years	4.9 (1.3 to 8.6)	0.9 (-1.2 to 3.0)	3.4 (0.9 to 5.9)	0.6 (-0.8 to 2.0)
Texas	20 to 24 years	6.4 (2.2 to 10.7)	2.6 (0.3 to 5.0)	4.1 (1.4 to 6.8)	1.7 (0.2 to 3.2)
Texas	25 to 29 years	4.4 (-0.7 to 9.4)	0.2 (-2.5 to 2.9)	3.0 (-0.5 to 6.6)	0.1 (-1.8 to 2.0)
Texas	30 to 34 years	7.5 (2.9 to 12.1)	0.1 (-2.4 to 2.7)	5.5 (2.1 to 8.9)	0.1 (-1.8 to 2.0)
Texas	35 to 39 years	8.4 (4.2 to 12.5)	0.9 (-1.5 to 3.2)	6.6 (3.3 to 9.9)	0.7 (-1.2 to 2.5)
Texas	40 to 44 years	8.2 (4.0 to 12.4)	1.8 (-0.5 to 4.1)	6.5 (3.2 to 9.9)	1.4 (-0.4 to 3.3)
Texas	45 to 49 years	8.0 (4.1 to 11.9)	2.4 (0.2 to 4.5)	6.3 (3.2 to 9.4)	1.9 (0.2 to 3.6)
Texas	5 to 9 years	-0.2 (-3.1 to 2.7)	0.3 (-1.3 to 1.9)	-0.1 (-1.9 to 1.7)	0.2 (-0.8 to 1.1)
Texas	50 to 54 years	6.0 (2.2 to 9.8)	2.4 (0.3 to 4.5)	4.8 (1.7 to 7.8)	1.9 (0.2 to 3.6)
Texas	55 to 59 years	2.6 (-1.2 to 6.3)	0.8 (-1.3 to 2.9)	2.0 (-1.0 to 4.9)	0.6 (-1.0 to 2.2)
Texas	60 to 64 years	3.8 (0.3 to 7.3)	1.9 (0.0 to 3.8)	2.9 (0.2 to 5.6)	1.5 (0.0 to 2.9)
Texas	65 to 69 years	1.0 (-0.9 to 2.8)	0.3 (-0.7 to 1.3)	0.9 (-0.8 to 2.6)	0.3 (-0.6 to 1.2)
Texas	70 to 74 years	-0.8 (-2.9 to 1.2)	-1.3 (-2.4 to -0.2)	-0.7 (-2.4 to 1.0)	-1.1 (-2.0 to -0.2)
Utah	0 to 11 months	-0.3 (-2.0 to 1.5)	0.0 (-1.0 to 0.9)	-0.2 (-1.5 to 1.1)	0.0 (-0.7 to 0.6)
Utah	1 to 4 years	1.0 (-1.5 to 3.6)	0.9 (-0.5 to 2.2)	0.5 (-0.7 to 1.6)	0.4 (-0.2 to 1.0)
Utah	10 to 14 years	-1.6 (-4.2 to 1.1)	-0.8 (-2.3 to 0.6)	-0.8 (-2.1 to 0.5)	-0.4 (-1.1 to 0.3)
Utah	15 to 19 years	2.9 (0.7 to 5.0)	0.5 (-0.7 to 1.7)	1.5 (0.4 to 2.6)	0.3 (-0.4 to 0.9)
Utah	20 to 24 years	4.0 (1.4 to 6.7)	1.6 (0.2 to 3.1)	1.4 (0.5 to 2.4)	0.6 (0.1 to 1.1)
Utah	25 to 29 years	2.8 (-0.4 to 6.1)	0.1 (-1.6 to 1.9)	1.4 (-0.2 to 3.0)	0.1 (-0.8 to 0.9)
Utah	30 to 34 years	4.3 (1.6 to 6.9)	0.1 (-1.4 to 1.5)	2.8 (1.1 to 4.5)	0.1 (-0.9 to 1.0)
Utah	35 to 39 years	5.4 (2.7 to 8.1)	0.6 (-0.9 to 2.1)	3.7 (1.8 to 5.5)	0.4 (-0.6 to 1.4)
Utah	40 to 44 years	5.1 (2.5 to 7.7)	1.1 (-0.3 to 2.5)	3.5 (1.7 to 5.4)	0.8 (-0.2 to 1.8)
Utah	45 to 49 years	4.9 (2.5 to 7.3)	1.5 (0.1 to 2.8)	3.6 (1.9 to 5.4)	1.1 (0.1 to 2.1)
Utah	5 to 9 years	-0.2 (-2.4 to 2.1)	0.2 (-1.0 to 1.4)	-0.1 (-1.1 to 1.0)	0.1 (-0.5 to 0.7)
Utah	50 to 54 years	3.8 (1.4 to 6.2)	1.5 (0.2 to 2.9)	2.9 (1.1 to 4.8)	1.2 (0.2 to 2.2)
Utah	55 to 59 years	1.5 (-0.7 to 3.8)	0.5 (-0.8 to 1.7)	0.9 (-0.5 to 2.3)	0.3 (-0.5 to 1.1)
Utah	60 to 64 years	2.4 (0.2 to 4.6)	1.2 (0.0 to 2.4)	1.8 (0.1 to 3.5)	0.9 (0.0 to 1.8)
Utah	65 to 69 years	0.6 (-0.6 to 1.8)	0.2 (-0.4 to 0.8)	0.4 (-0.3 to 1.1)	0.1 (-0.3 to 0.5)

© 2021 Weaver MR et al. *JAMA Network Open*.

Utah	70 to 74 years	-0.3 (-1.0 to 0.4)	-0.4 (-0.8 to -0.1)	-0.2 (-0.8 to 0.4)	-0.4 (-0.7 to -0.1)
Vermont	0 to 11 months	0.0 (-0.2 to 0.2)	0.0 (-0.1 to 0.1)	0.0 (-0.3 to 0.2)	0.0 (-0.1 to 0.1)
Vermont	1 to 4 years	0.1 (-0.1 to 0.3)	0.1 (0.0 to 0.2)	0.1 (-0.2 to 0.4)	0.1 (-0.1 to 0.3)
Vermont	10 to 14 years	-0.5 (-1.3 to 0.3)	-0.3 (-0.7 to 0.2)	-0.1 (-0.3 to 0.1)	-0.1 (-0.2 to 0.0)
Vermont	15 to 19 years	0.9 (0.2 to 1.6)	0.2 (-0.2 to 0.6)	0.3 (0.1 to 0.5)	0.1 (-0.1 to 0.2)
Vermont	20 to 24 years	2.4 (0.8 to 3.9)	1.0 (0.1 to 1.8)	0.5 (0.2 to 0.9)	0.2 (0.0 to 0.4)
Vermont	25 to 29 years	1.8 (-0.3 to 3.9)	0.1 (-1.0 to 1.2)	0.9 (-0.1 to 2.0)	0.0 (-0.5 to 0.6)
Vermont	30 to 34 years	3.5 (1.3 to 5.6)	0.1 (-1.1 to 1.2)	1.1 (0.4 to 1.9)	0.0 (-0.4 to 0.4)
Vermont	35 to 39 years	3.1 (1.6 to 4.7)	0.3 (-0.5 to 1.2)	2.2 (1.1 to 3.3)	0.2 (-0.4 to 0.8)
Vermont	40 to 44 years	3.1 (1.5 to 4.6)	0.7 (-0.2 to 1.5)	1.9 (0.9 to 2.9)	0.4 (-0.1 to 1.0)
Vermont	45 to 49 years	3.4 (1.7 to 5.0)	1.0 (0.1 to 1.9)	1.8 (0.9 to 2.7)	0.5 (0.1 to 1.0)
Vermont	5 to 9 years	0.0 (-0.8 to 0.7)	0.1 (-0.3 to 0.5)	0.0 (-0.2 to 0.2)	0.0 (-0.1 to 0.1)
Vermont	50 to 54 years	2.4 (0.9 to 3.9)	1.0 (0.1 to 1.8)	0.6 (0.2 to 1.0)	0.2 (0.0 to 0.5)
Vermont	55 to 59 years	0.7 (-0.3 to 1.7)	0.2 (-0.3 to 0.8)	0.7 (-0.3 to 1.6)	0.2 (-0.3 to 0.7)
Vermont	60 to 64 years	1.1 (0.1 to 2.1)	0.5 (0.0 to 1.1)	0.6 (0.0 to 1.2)	0.3 (0.0 to 0.6)
Vermont	65 to 69 years	0.1 (-0.1 to 0.2)	0.0 (-0.1 to 0.1)	0.2 (-0.2 to 0.7)	0.1 (-0.2 to 0.3)
Vermont	70 to 74 years	0.0 (0.0 to 0.0)	0.0 (0.0 to 0.0)	0.0 (0.0 to 0.0)	0.0 (0.0 to 0.0)
Virginia	0 to 11 months	-0.2 (-1.4 to 1.0)	0.0 (-0.7 to 0.6)	-0.2 (-1.1 to 0.8)	0.0 (-0.5 to 0.5)
Virginia	1 to 4 years	0.5 (-0.7 to 1.7)	0.4 (-0.2 to 1.1)	0.4 (-0.6 to 1.4)	0.3 (-0.2 to 0.9)
Virginia	10 to 14 years	-1.0 (-2.7 to 0.7)	-0.5 (-1.4 to 0.4)	-0.7 (-1.8 to 0.4)	-0.3 (-0.9 to 0.3)
Virginia	15 to 19 years	2.1 (0.5 to 3.6)	0.4 (-0.5 to 1.3)	1.6 (0.4 to 2.8)	0.3 (-0.4 to 0.9)
Virginia	20 to 24 years	3.8 (1.3 to 6.4)	1.6 (0.2 to 3.0)	2.1 (0.7 to 3.4)	0.9 (0.1 to 1.6)
Virginia	25 to 29 years	2.6 (-0.4 to 5.6)	0.1 (-1.5 to 1.7)	1.7 (-0.2 to 3.6)	0.1 (-1.0 to 1.1)
Virginia	30 to 34 years	4.2 (1.6 to 6.7)	0.1 (-1.3 to 1.5)	2.9 (1.1 to 4.7)	0.1 (-0.9 to 1.0)
Virginia	35 to 39 years	4.1 (2.1 to 6.2)	0.4 (-0.7 to 1.6)	3.8 (1.9 to 5.7)	0.4 (-0.7 to 1.5)
Virginia	40 to 44 years	4.5 (2.2 to 6.8)	1.0 (-0.3 to 2.3)	3.6 (1.7 to 5.4)	0.8 (-0.2 to 1.8)
Virginia	45 to 49 years	4.2 (2.1 to 6.2)	1.2 (0.1 to 2.4)	3.3 (1.7 to 4.9)	1.0 (0.1 to 1.9)
Virginia	5 to 9 years	-0.1 (-1.4 to 1.2)	0.1 (-0.6 to 0.8)	-0.1 (-1.0 to 0.9)	0.1 (-0.4 to 0.6)
Virginia	50 to 54 years	3.2 (1.1 to 5.2)	1.3 (0.2 to 2.4)	2.6 (0.9 to 4.2)	1.0 (0.1 to 2.0)

© 2021 Weaver MR et al. *JAMA Network Open*.

Virginia	55 to 59 years	1.4 (-0.7 to 3.5)	0.4 (-0.7 to 1.6)	1.1 (-0.5 to 2.7)	0.3 (-0.5 to 1.2)
Virginia	60 to 64 years	2.1 (0.2 to 4.1)	1.1 (0.0 to 2.2)	1.6 (0.1 to 3.0)	0.8 (0.0 to 1.6)
Virginia	65 to 69 years	0.4 (-0.4 to 1.3)	0.1 (-0.3 to 0.6)	0.4 (-0.3 to 1.0)	0.1 (-0.3 to 0.5)
Virginia	70 to 74 years	-0.5 (-1.6 to 0.7)	-0.7 (-1.3 to -0.1)	-0.2 (-0.8 to 0.3)	-0.4 (-0.7 to -0.1)
Washington	0 to 11 months	-0.2 (-1.6 to 1.2)	0.0 (-0.8 to 0.7)	-0.1 (-0.5 to 0.4)	0.0 (-0.3 to 0.2)
Washington	1 to 4 years	0.5 (-0.7 to 1.8)	0.4 (-0.2 to 1.1)	0.2 (-0.2 to 0.6)	0.1 (-0.1 to 0.4)
Washington	10 to 14 years	-0.9 (-2.4 to 0.6)	-0.5 (-1.3 to 0.3)	-0.4 (-0.9 to 0.2)	-0.2 (-0.5 to 0.1)
Washington	15 to 19 years	2.5 (0.6 to 4.4)	0.5 (-0.6 to 1.5)	0.9 (0.2 to 1.6)	0.2 (-0.2 to 0.6)
Washington	20 to 24 years	4.6 (1.6 to 7.7)	1.9 (0.2 to 3.6)	1.4 (0.5 to 2.4)	0.6 (0.1 to 1.1)
Washington	25 to 29 years	3.1 (-0.5 to 6.6)	0.1 (-1.8 to 2.1)	1.2 (-0.2 to 2.6)	0.1 (-0.7 to 0.8)
Washington	30 to 34 years	4.9 (1.9 to 7.9)	0.1 (-1.6 to 1.7)	2.1 (0.8 to 3.5)	0.0 (-0.7 to 0.8)
Washington	35 to 39 years	5.0 (2.5 to 7.5)	0.5 (-0.9 to 1.9)	2.5 (1.3 to 3.8)	0.3 (-0.4 to 1.0)
Washington	40 to 44 years	4.7 (2.3 to 7.1)	1.0 (-0.3 to 2.4)	2.6 (1.2 to 3.9)	0.6 (-0.2 to 1.3)
Washington	45 to 49 years	4.8 (2.5 to 7.1)	1.4 (0.1 to 2.7)	2.3 (1.2 to 3.4)	0.7 (0.1 to 1.3)
Washington	5 to 9 years	-0.1 (-1.3 to 1.1)	0.1 (-0.5 to 0.8)	0.0 (-0.5 to 0.5)	0.0 (-0.2 to 0.3)
Washington	50 to 54 years	3.3 (1.2 to 5.5)	1.3 (0.2 to 2.5)	1.7 (0.6 to 2.8)	0.7 (0.1 to 1.3)
Washington	55 to 59 years	1.5 (-0.7 to 3.7)	0.5 (-0.8 to 1.7)	0.7 (-0.3 to 1.7)	0.2 (-0.3 to 0.8)
Washington	60 to 64 years	2.0 (0.2 to 3.9)	1.0 (0.0 to 2.1)	1.1 (0.1 to 2.0)	0.5 (0.0 to 1.1)
Washington	65 to 69 years	0.4 (-0.4 to 1.2)	0.1 (-0.3 to 0.5)	0.3 (-0.2 to 0.7)	0.1 (-0.2 to 0.3)
Washington	70 to 74 years	-0.5 (-1.6 to 0.7)	-0.7 (-1.3 to -0.1)	-0.3 (-1.0 to 0.4)	-0.4 (-0.8 to -0.1)
West Virginia	0 to 11 months	-0.1 (-0.9 to 0.6)	0.0 (-0.4 to 0.4)	-0.1 (-0.6 to 0.5)	0.0 (-0.3 to 0.3)
West Virginia	1 to 4 years	0.4 (-0.6 to 1.4)	0.3 (-0.2 to 0.8)	0.1 (-0.2 to 0.5)	0.1 (-0.1 to 0.3)
West Virginia	10 to 14 years	-0.6 (-1.7 to 0.4)	-0.3 (-0.9 to 0.2)	-0.2 (-0.6 to 0.1)	-0.1 (-0.3 to 0.1)
West Virginia	15 to 19 years	2.8 (0.7 to 4.8)	0.5 (-0.7 to 1.7)	0.7 (0.2 to 1.3)	0.1 (-0.2 to 0.4)
West Virginia	20 to 24 years	4.9 (1.7 to 8.2)	2.0 (0.2 to 3.8)	1.2 (0.4 to 2.0)	0.5 (0.1 to 1.0)
West Virginia	25 to 29 years	3.4 (-0.5 to 7.4)	0.2 (-2.0 to 2.3)	1.4 (-0.2 to 3.0)	0.1 (-0.8 to 0.9)
West Virginia	30 to 34 years	6.0 (2.3 to 9.6)	0.1 (-1.9 to 2.1)	2.2 (0.8 to 3.5)	0.0 (-0.7 to 0.8)
West Virginia	35 to 39 years	6.0 (3.0 to 8.9)	0.6 (-1.0 to 2.3)	2.5 (1.2 to 3.7)	0.3 (-0.4 to 0.9)
West Virginia	40 to 44 years	5.5 (2.7 to 8.4)	1.2 (-0.4 to 2.8)	2.3 (1.1 to 3.5)	0.5 (-0.2 to 1.1)

© 2021 Weaver MR et al. *JAMA Network Open*.

West Virginia	45 to 49 years	6.3 (3.2 to 9.4)	1.9 (0.2 to 3.6)	2.5 (1.3 to 3.7)	0.7 (0.1 to 1.4)
West Virginia	5 to 9 years	0.0 (-0.7 to 0.6)	0.1 (-0.3 to 0.4)	0.0 (-0.3 to 0.3)	0.0 (-0.1 to 0.2)
West Virginia	50 to 54 years	3.9 (1.4 to 6.3)	1.6 (0.2 to 2.9)	1.9 (0.7 to 3.2)	0.8 (0.1 to 1.5)
West Virginia	55 to 59 years	1.6 (-0.8 to 4.0)	0.5 (-0.8 to 1.8)	0.5 (-0.3 to 1.3)	0.2 (-0.3 to 0.6)
West Virginia	60 to 64 years	2.4 (0.2 to 4.6)	1.2 (0.0 to 2.4)	1.1 (0.1 to 2.2)	0.6 (0.0 to 1.2)
West Virginia	65 to 69 years	0.3 (-0.3 to 0.9)	0.1 (-0.2 to 0.4)	0.3 (-0.3 to 0.8)	0.1 (-0.2 to 0.4)
West Virginia	70 to 74 years	-0.3 (-1.0 to 0.4)	-0.5 (-0.8 to -0.1)	-0.1 (-0.3 to 0.1)	-0.1 (-0.2 to 0.0)
Wisconsin	0 to 11 months	-0.2 (-1.2 to 0.9)	0.0 (-0.6 to 0.5)	-0.1 (-0.9 to 0.6)	0.0 (-0.4 to 0.4)
Wisconsin	1 to 4 years	0.5 (-0.7 to 1.6)	0.4 (-0.2 to 1.0)	0.3 (-0.4 to 1.1)	0.3 (-0.1 to 0.7)
Wisconsin	10 to 14 years	-0.8 (-2.1 to 0.5)	-0.4 (-1.1 to 0.3)	-0.4 (-1.1 to 0.3)	-0.2 (-0.6 to 0.2)
Wisconsin	15 to 19 years	2.2 (0.6 to 3.8)	0.4 (-0.5 to 1.3)	0.9 (0.2 to 1.6)	0.2 (-0.2 to 0.6)
Wisconsin	20 to 24 years	3.4 (1.2 to 5.7)	1.4 (0.2 to 2.7)	1.6 (0.5 to 2.6)	0.6 (0.1 to 1.2)
Wisconsin	25 to 29 years	2.0 (-0.3 to 4.4)	0.1 (-1.2 to 1.4)	1.1 (-0.2 to 2.4)	0.1 (-0.6 to 0.7)
Wisconsin	30 to 34 years	3.2 (1.2 to 5.1)	0.1 (-1.0 to 1.1)	2.0 (0.8 to 3.2)	0.0 (-0.6 to 0.7)
Wisconsin	35 to 39 years	2.9 (1.5 to 4.4)	0.3 (-0.5 to 1.1)	2.1 (1.0 to 3.1)	0.2 (-0.4 to 0.8)
Wisconsin	40 to 44 years	3.6 (1.7 to 5.5)	0.8 (-0.2 to 1.8)	2.2 (1.1 to 3.3)	0.5 (-0.1 to 1.1)
Wisconsin	45 to 49 years	3.2 (1.6 to 4.7)	0.9 (0.1 to 1.8)	2.0 (1.0 to 3.0)	0.6 (0.1 to 1.1)
Wisconsin	5 to 9 years	-0.1 (-0.9 to 0.7)	0.1 (-0.4 to 0.5)	0.0 (-0.7 to 0.6)	0.1 (-0.3 to 0.4)
Wisconsin	50 to 54 years	2.5 (0.9 to 4.2)	1.0 (0.1 to 1.9)	1.4 (0.5 to 2.3)	0.6 (0.1 to 1.1)
Wisconsin	55 to 59 years	1.1 (-0.5 to 2.6)	0.3 (-0.5 to 1.2)	0.6 (-0.3 to 1.4)	0.2 (-0.3 to 0.6)
Wisconsin	60 to 64 years	1.6 (0.1 to 3.1)	0.8 (0.0 to 1.6)	1.0 (0.1 to 1.9)	0.5 (0.0 to 1.0)
Wisconsin	65 to 69 years	0.1 (-0.1 to 0.4)	0.0 (-0.1 to 0.2)	0.1 (-0.1 to 0.3)	0.0 (-0.1 to 0.2)
Wisconsin	70 to 74 years	-0.1 (-0.4 to 0.2)	-0.2 (-0.3 to 0.0)	-0.1 (-0.4 to 0.2)	-0.2 (-0.4 to 0.0)
Wyoming	0 to 11 months	-0.4 (-2.7 to 2.0)	-0.1 (-1.3 to 1.2)	-0.3 (-2.4 to 1.7)	-0.1 (-1.1 to 1.0)
Wyoming	1 to 4 years	0.8 (-1.1 to 2.7)	0.7 (-0.4 to 1.7)	0.8 (-1.1 to 2.7)	0.7 (-0.3 to 1.7)
Wyoming	10 to 14 years	-0.8 (-2.2 to 0.6)	-0.4 (-1.2 to 0.3)	-1.1 (-2.9 to 0.7)	-0.6 (-1.5 to 0.4)
Wyoming	15 to 19 years	3.2 (0.8 to 5.6)	0.6 (-0.8 to 1.9)	1.5 (0.4 to 2.5)	0.3 (-0.3 to 0.9)
Wyoming	20 to 24 years	4.3 (1.5 to 7.2)	1.8 (0.2 to 3.4)	2.5 (0.9 to 4.2)	1.0 (0.1 to 2.0)
Wyoming	25 to 29 years	2.7 (-0.4 to 5.8)	0.1 (-1.6 to 1.8)	2.5 (-0.4 to 5.4)	0.1 (-1.5 to 1.7)

© 2021 Weaver MR et al. *JAMA Network Open*.

Wyoming	30 to 34 years	5.5 (2.1 to 8.9)	0.1 (-1.8 to 2.0)	3.6 (1.4 to 5.8)	0.1 (-1.2 to 1.3)
Wyoming	35 to 39 years	5.5 (2.7 to 8.2)	0.6 (-1.0 to 2.1)	3.1 (1.5 to 4.6)	0.3 (-0.5 to 1.2)
Wyoming	40 to 44 years	4.1 (2.0 to 6.1)	0.9 (-0.3 to 2.0)	3.4 (1.6 to 5.1)	0.7 (-0.2 to 1.7)
Wyoming	45 to 49 years	6.1 (3.1 to 9.0)	1.8 (0.2 to 3.4)	4.4 (2.3 to 6.6)	1.3 (0.1 to 2.5)
Wyoming	5 to 9 years	-0.1 (-0.9 to 0.8)	0.1 (-0.4 to 0.5)	-0.1 (-1.8 to 1.5)	0.2 (-0.7 to 1.0)
Wyoming	50 to 54 years	4.5 (1.6 to 7.4)	1.8 (0.2 to 3.4)	4.1 (1.5 to 6.7)	1.7 (0.2 to 3.1)
Wyoming	55 to 59 years	1.3 (-0.6 to 3.2)	0.4 (-0.7 to 1.5)	1.7 (-0.8 to 4.1)	0.5 (-0.8 to 1.9)
Wyoming	60 to 64 years	1.8 (0.1 to 3.4)	0.9 (0.0 to 1.8)	1.8 (0.1 to 3.4)	0.9 (0.0 to 1.8)
Wyoming	65 to 69 years	0.8 (-0.7 to 2.3)	0.3 (-0.5 to 1.1)	0.7 (-0.7 to 2.1)	0.2 (-0.5 to 1.0)
Wyoming	70 to 74 years	0.0 (0.0 to 0.0)	0.0 (0.0 to 0.0)	-0.1 (-0.4 to 0.2)	-0.2 (-0.4 to 0.0)

© 2021 Weaver MR et al. *JAMA Network Open*.
